# Pancreatic Cancer Exposome Profile to Aid Early Detection and Inform Prevention Strategies

**DOI:** 10.3390/jcm10081665

**Published:** 2021-04-13

**Authors:** Maria J. Monroy-Iglesias, Saoirse Dolly, Debashis Sarker, Kiruthikah Thillai, Mieke Van Hemelrijck, Aida Santaolalla

**Affiliations:** 1 Translational Oncology & Urology Research (TOUR), School of Cancer and Pharmaceutical Sciences, King’s College London, London SE1 9RT, UK; maria.j.monroy_iglesias@kcl.ac.uk (M.J.M.-I.); mieke.vanhemelrijck@kcl.ac.uk (M.V.H.); 2 Department of Medical Oncology, Guy’s and St Thomas’ NHS Foundation Trust, London SE1 9RT, UK; Saoirse.Dolly@gstt.nhs.uk (S.D.); Debashis.Sarker@kcl.ac.uk (D.S.); Kiruthikah.Thillai@gstt.nhs.uk (K.T.); 3 School of Cancer and Pharmaceutical Sciences, King’s College London, London SE1 9RT, UK

**Keywords:** pancreatic cancer, exposome, systematic review, early detection, cancer risk factors

## Abstract

Pancreatic cancer (PCa) is associated with a poor prognosis and high mortality rate. The causes of PCa are not fully elucidated yet, although certain exposome factors have been identified. The exposome is defined as the sum of all environmental factors influencing the occurrence of a disease during a life span. The development of an exposome approach for PCa has the potential to discover new disease-associated factors to better understand the carcinogenesis of PCa and help with early detection strategies. Our systematic review of the literature identified several exposome factors that have been associated with PCa alone and in combination with other exposures. A potential inflammatory signature has been observed among the interaction of several exposures (i.e., smoking, alcohol consumption, diabetes mellitus, obesity, and inflammatory markers) that further increases the incidence and progression of PCa. A large number of exposures have been identified such as genetic, hormonal, microorganism infections and immune responses that warrant further investigation. Future early detection strategies should utilize this information to assess individuals’ risk for PCa.



**Summary Box.**

**What is already known about risk factors for PCa**
Cigarette smoking, family history of PCa, diabetes mellitus, cystic lesions of pancreas, and pancreatitis are well-established risk factors for PCa.
**What our systematic review has revealed about the exposome for PCa**
We provided a template for a generalized approach to identify risk factors that contribute to PCa incidence and progression. We identified a list of non-modifiable factors that could alert clinicians of at-risk individuals, and modifiable factors which can be controlled in order to decrease PCa incidence. We observed that several of these factors are associated with the inflammatory pathway which suggest that PCa carcinogenesis is driven by inflammatory processes.
**Non-Modifiable Factors:**
Non-O blood group;Diabetes mellitus;Pancreatitis;Metabolic syndrome;Family history of PCa;Genetic factors;Cystic lesions;Inflammatory markers.

**Modifiable Factors:**
Smoking;Alcohol consumption;Dietary factors;Obesity;Metabolic syndrome;Infectious agents (HBV, pathogenic oral bacteria);Pesticide exposure;Allergies.



## 1. Introduction

Pancreatic cancer (PCa) is one of the leading causes of cancer mortality worldwide, particularly in more developed countries [1]. PCa ranks seventh for cancer mortality globally, accounting for 495,773 new cases and causing 466,003 deaths in 2020 [2]. The lack of specific symptoms means PCa is often diagnosed at an advanced stage and remains one of the most lethal malignancies with a 5-year survival rate of 5–10% [3,4].

Pancreatic ductal adenocarcinoma (PDAC) is an exocrine tumour with no clear cellular origin [5] and accounts for 90% of all pancreatic neoplasms [6,7]. PCa rarely manifests with specific signs or symptoms. Patients with PCa usually develop symptoms such as asthenia, abdominal pain and weight loss, and only seek medical attention if symptoms persist or they develop obstructive symptoms such as jaundice [8]. The majority of cases are often already incurable at the time of diagnosis, and even with palliative chemotherapy have a median overall survival of 6–12 months [9]. Patients with early-stage disease can be offered radical surgical resection and adjuvant chemotherapy. However, the risk of recurrence is high and the majority of patients relapse, with a 5-year survival rate of 29% [10].

Aetiologically, PCa is a multifactorial disease with pathogenesis related to both genetic and environmental factors [11]. Although various exposures such as cigarette smoking, family history of PCa, diabetes mellitus (DM) and cystic lesions of the pancreas have been identified as risk factors, few studies have explored the synergy between these factors and their interactions with common biological pathways in relation to PCa development [7]. Furthermore, new strategies to untangle carcinogenesis are needed to inform with early detection strategies to make a clinically significant impact on prognosis [12].

**Figure 1:**
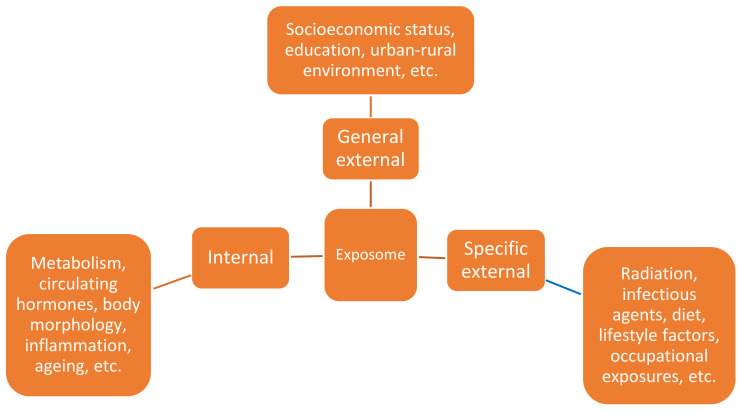
Overview of three different domains of the exposome with examples [17].

The concept of the exposome was first defined by Dr Christopher Wild in 2005, who suggested it as a tool to address the need for a more complete environmental exposure (i.e., non-genetic) assessment in epidemiological studies [13]. The exposome concept refers to the totality of exposures from various external and internal sources (i.e., biological agents, chemical agents, radiation, psychosocial exposures, etc.), to which an individual is subjected from conception to death [14]. Therefore, the exposome complements the genome by providing a comprehensive description of lifelong exposures. A total of three broad categories of non-genetic exposures have been defined: internal, specific external and general external exposures (Figure 1) [15]. Internal exposures comprise of physiologically processes such as metabolism, circulating hormones, inflammation, body morphology, and ageing. Specific external exposures include an extensive range of factors such as infectious agents, diet, lifestyle factors (i.e., tobacco, alcohol), occupational exposures, and radiation. General external exposures include the social, economic and psychological influences of an individual [16]. In the exposome, these domains overlap and can be linked, so one domain may be a response to another [16]. For example, lower socioeconomic status (general external) is often associated with a higher body mass index (BMI) [17], which has been linked to chronic inflammation (internal) [18]. Moreover, practical implementation of the exposome approach has been widely used in several fields [19,20,21].

Under the exposome framework, epidemiological evidence allows investigation of the potential interplay between several exposures to determine the complicated aetiology of pancreatic neoplasms. For instance, chronic pancreatitis resulting in acinar cell injury due to alcohol consumption, is also a complex chronic inflammatory disorder linked to genetic, metabolic, and other environmental factors [22]. Following the exposome approach, the current review aimed to identify a PCa exposure profile (combination and overlap of exposures), which may predict an increased risk of an individual developing PCa—an approach which will inform future strategies for prevention and early detection.

## 2. Materials and Methods

The present study was conducted in accordance with the widely accepted Preferred Reporting Items for Systematic Reviews and Meta-analyses (PRISMA) guidelines [23] to ensure an adequate reporting of the systematic review. No protocol has been registered for the study. A literature search of epidemiological studies was conducted on 3 December 2020 using the search engine in PubMed, a bibliographic database including over 30 million references to journal articles, with the following search terms: (“Pancreatic Neoplasms”[Mesh] OR “cancer of the pancreas” [Title/Abstract] OR “pancreatic cancer” [Title/Abstract] OR “pancreatic tumour” [Title/Abstract]) and (“Risk Factors”[Mesh] OR “Biomarkers”[Mesh] OR “Exposome”[Mesh] OR “Causality”[Mesh] AND “aetiology” [Subheading]). The search was restricted to human studies published from January 1973 to December 2020. The full search strategy is included in Table 1.

The inclusion criteria considered studies on adults only. No restrictions were placed on publication type, with all systematic reviews, narrative reviews, meta-analyses, original research articles (experimental, observational and clinical trials), commentaries, letters, and editorials identified in the PUBMED search being considered eligible. Non-English publications, duplicate studies, preprints, errata and animal studies were excluded. Moreover, only publications with full text available were included.

Publications were initially screened by title and abstract, with potentially relevant studies undergoing a full text review. Following the inclusion and exclusion criteria described above, some studies were further excluded in the full text review. Only publications that fell under one of the three previously mentioned exposome categories (general external, specific external and internal) were included. No publications on survival or diagnostic markers were included. The following study characteristics were extracted for the publications included in the review: name of first author, year of publication, study location, study design, population, exposures, detailed findings, and other observations.

## 3. Results

**Figure 2:**
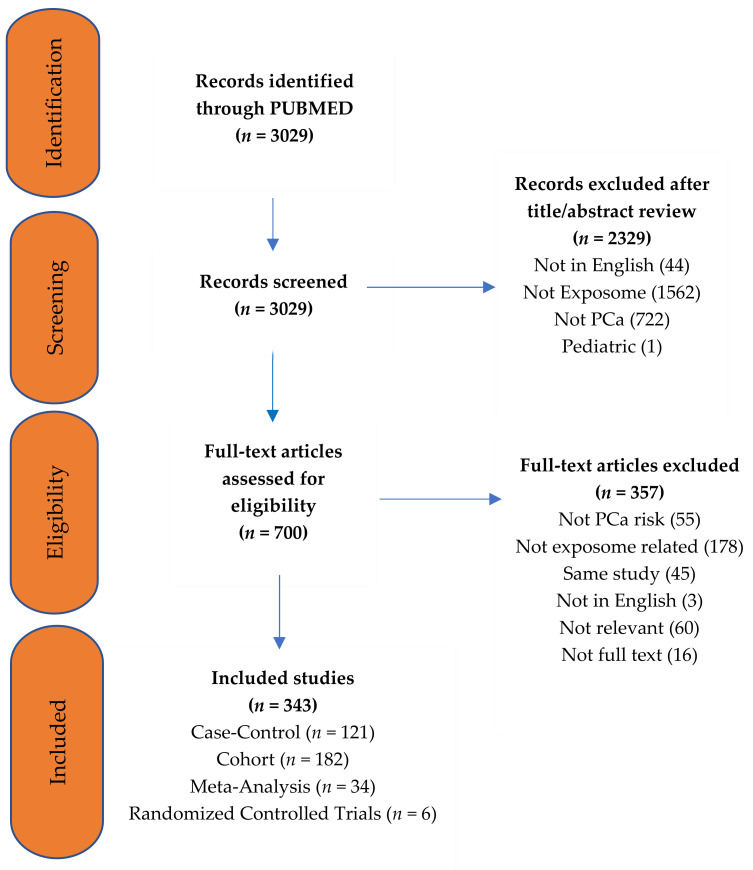
PRISMA diagram representing the review strategy [13].

A total of 3029 studies were identified in the PubMed search. Full text review was undertaken on 700 potentially eligible articles after title and abstract screening. A total of 343 publications were included consisting of: 34 systematic reviews with meta-analyses, 6 randomized trials, and 303 observational studies (Figure 2). The remaining publications were reviews, letters, editorials, commentaries, and position statements.

**Figure 3:**
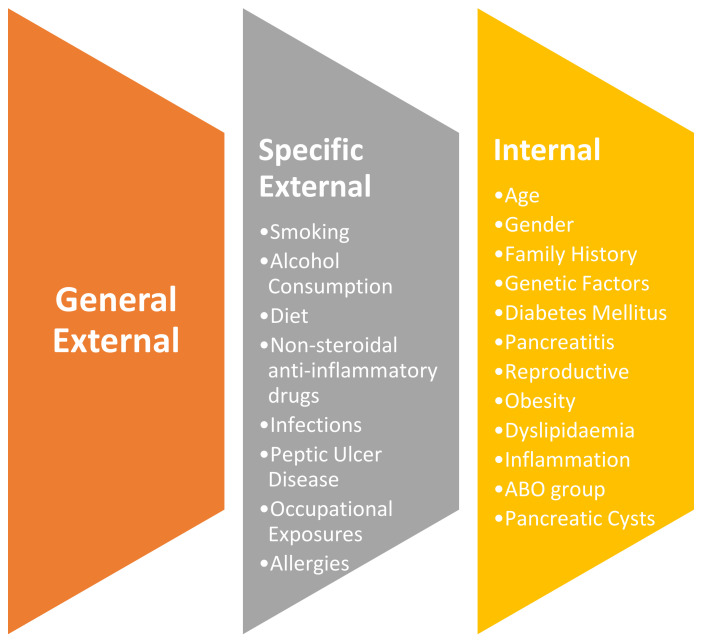
Overview of the internal, specific external and general external exposome factors for pancreatic cancer described in the review.

**Figure 4:**
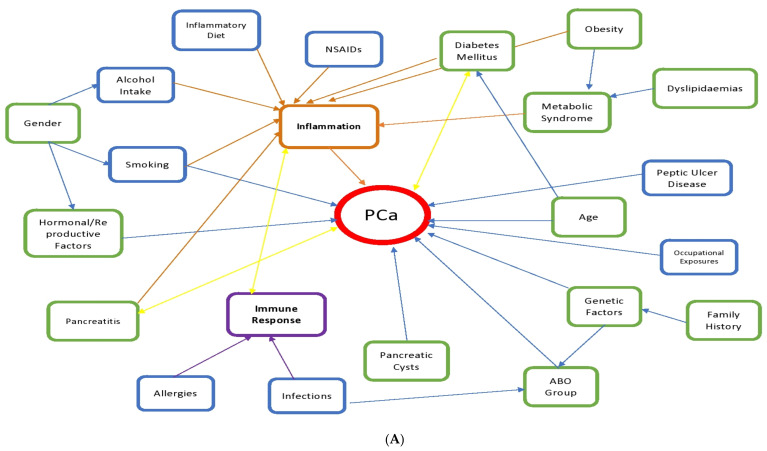

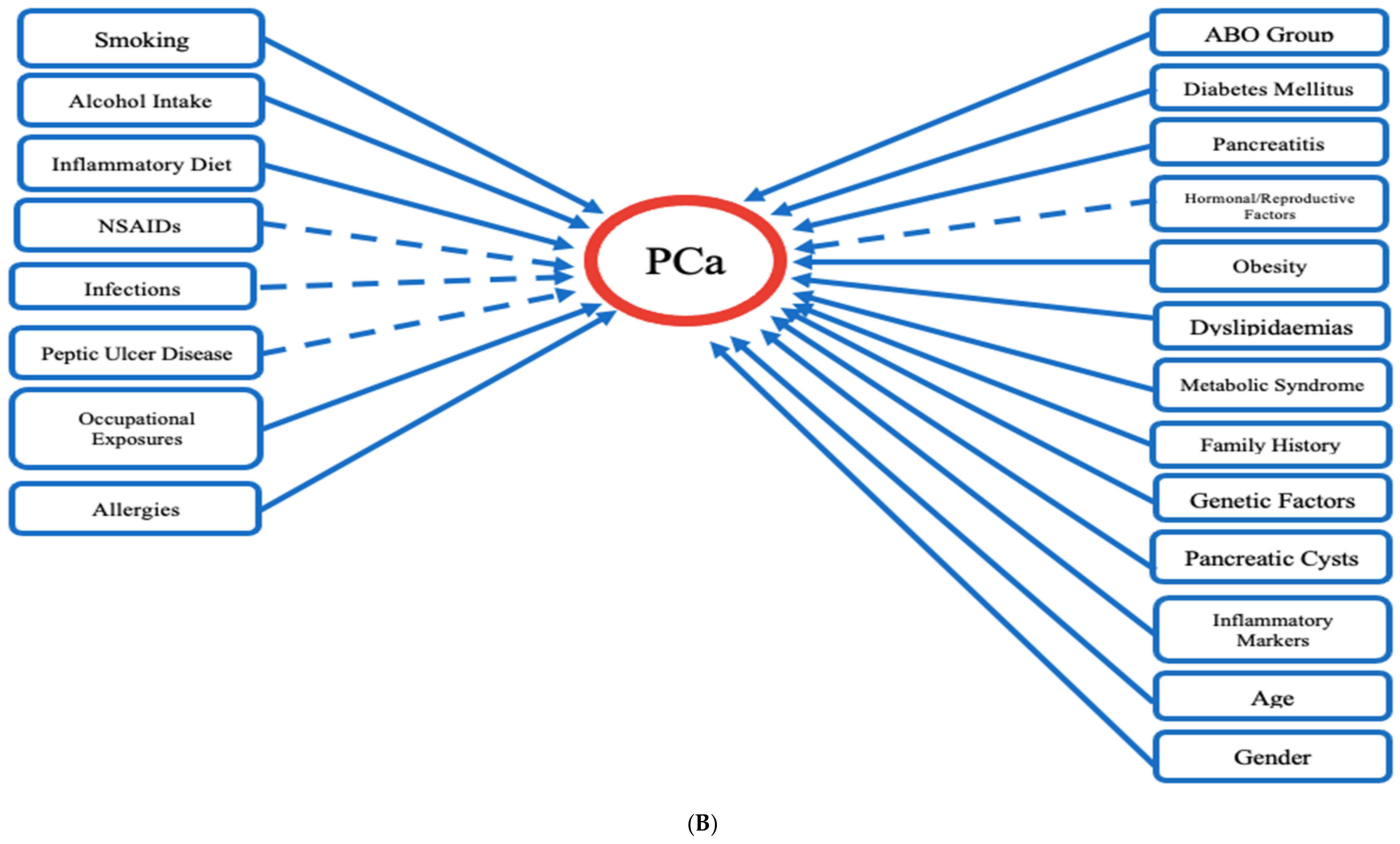
(**A**) Schematic representation of exposure variables included in the current study and their interactions *. * All variables are hypothesized risk factors for pancreatic cancer (PCa) risk (illustrated in red). Blue variables illustrate specific external risk factors; green variables illustrate internal risk factors; orange variable illustrates the inflammation pathway; purple variable illustrate the immune response pathway. The blue arrow illustrates a direct unidirectional association between variables and PCa; the orange arrow illustrates variables associated with the inflammation pathway; the purple arrow variables associated with the immune response; the yellow arrows bidirectional association between variables and PCa. (**B**) Schematic representation of the strength of the association with PCa of the risk factors identified in this systematic review. Strength of the association is defined considering the number of studies investigating the exposure, the range of the hazard ratio/odds ratio/relative risk/standardized incidence ratio reported in each study, and the statistical significance. * All variables are risk factors for pancreatic cancer (PCa) risk (illustrated in red). Bold arrows illustrate a strong association between variables and PCa risk. Large-dotted arrows illustrate variables with intermediate associations. Short-dotted arrows illustrate variables with weak associations.

To characterize the risk profile of PCa using the exposome approach, we have reported studies based on whether they assessed a component of internal, general external or specific external exposure and/or a combination of exposure patterns. No studies were found where general external exposures (i.e., socioeconomic status (SES), education level, ethnicity) were investigated as primary exposure variables in relation to risk of PCa; however, our literature review identified several specific external and internal exposures including the well-established PCa risk factors, such as smoking, alcohol consumption, diet, family history, genetic factors, diabetes mellitus, pancreatitis, together with less established exposures such as medicine use, infections, peptic ulcer disease, occupational exposures, allergies, obesity, dyslipidaemias, inflammation, and ABO blood group (Figure 3). Table 2 summarizes the exposures that define the PCa exposome profile and the strength of the association with PCa risk. A more detailed overview of these studies is given in Supplementary Tables S1 and S2. Both direct and indirect evidence of the interaction of the exposures, specific external and internal, and PCa is discussed below. Moreover, the complex interaction observed between the exposures is illustrated in Figure 4A, and the strength of the association between the different risk factors and PCa risk is illustrated in Figure 4B.

### 3.1. Specific External Exposures

#### 3.1.1. Smoking

Cigarette smoking has been consistently identified as an important environmental risk factor for PCa [1]. However, the effect of non-cigarette and passive smoking on PCa remains unclear. There is reasonable amount of evidence suggesting that former and current smoking increases the risk of PCa [11,24,25,26,27,28,29,30]. A recent meta-analysis reported an increased PCa risk of 66% and 40% in current and former smokers, respectively, compared to never smokers [26]. This risk has been seen to increase with greater intensity and pack-years [31,32,33]. Additionally, a cohort study by Heinen et al. found that quitting smoking gradually reduced PCa risk, approaching unity after ≥ 20 years of quitting [33]; this was supported by one other observational study [34]. Moreover, two cohort studies reported that current smoking present a higher PCa risk in women compared to men [35,36].

Cigarette smoking has been thought to interact with many factors that increase PCa risk. A large case control study reported that current smoking, in addition to an inflammatory diet, has a four-fold increase in PCa risk [24]. Moreover, a case–control study including 808 cases of PCa found synergistic interactions between cigarette smoking and family history (FH) of PCa and DM [37]. This association was supported by another case–control study looking at the same factors combined and found a 10-fold increase in PCa risk [27].

Information on whether environmental tobacco smoke (ETS) and passive smoking increases the risk of PCa remains elusive. A case–control study by Lo et al. reported a positive association between passive smoking and PCa risk (Odds Ratio (OR) 6.0; 95% confidence interval (CI) 2.4–14.8) [38], this result was consistent with three other observational studies [25,30,39]. However, two other studies found no significant association [40,41]. As for non-cigarette smoking, various studies found a two-fold increased risk of PCa in cigar smokers [25,38]. One study reported a greater PCa risk in patients who were both cigarette and non-cigarette smokers (OR 7.8; 95%CI 3.0–20.6) [38].

#### 3.1.2. Alcohol Consumption

Alcohol has long been suspected as a risk factor for PCa because of its association to chronic pancreatitis [7]. Most studies have reported a positive association between alcohol consumption and PCa risk [34,37,42,43,44,45,46,47,48], while others have found no association [31,49,50,51,52]. A consistent positive association of PCa risk has been reported particularly with heavy alcohol consumption (defined differently in each study included, but with an average estimate of 40 gr/day) [26,45,46]. Moreover, a large case–control study found a positive association depending on dose, duration and pattern of drinking among men, but not women [44]. Conversely, two meta-analyses reported a positive association between heavy drinkers and higher PCa risk in women [26,43].

Alcohol consumption in combination with other risk factors has been suggested to increase potential for PCa. A recent case–control study reported a stronger association with PCa risk, when alcohol consumption was combined with DM, smoking and hypercholesterolemia (OR 3.29 (95%CI 1.89–5.73), 3.31 (95%CI 1.50–7.32), 5.11 (95%CI 2.66–9.82), respectively). Moreover, according to a large recent cohort, baseline alcohol intake from beer and spirits/liquors were associated with a higher risk estimates than wine [48]. A recent meta-analysis looking at early onset PCa reported an increased risk of early-onset PCa (EOPC, <60 years) (OR 1.49; 95%CI 1.21–1.84) and very early onset PCa (VEOPC, <45 years) (OR 2.18; 95%CI 1.17–4.09). Another pooled analysis with data from two large cohorts reported similar results where current drinkers were diagnosed with PCa at a significantly younger age than their never-drinking counterparts [34,47].

#### 3.1.3. Diet

Several epidemiological studies have focused on dietary factors as an important risk factor for the development of PCa. A recent meta-analysis of 32 studies evaluated the association between dietary patterns and PCa risk. Healthy eating pattern was defined as high intake of vegetables, fruits, whole grains, olive oil, fish, soy, poultry and low-fat dairy and was associated with a decreased risk of PCa (OR 0.86; 95%CI 0.77–0.95) versus the least healthy eating patterns. The same study reported an increased risk of PCa with a western-type diet (OR 1.24; 95%CI 1.06–1.45), characterized as high consumption of red and/or processed meat, refined grains, sweets, high-fat dairy products, butter, potatoes and high-fat gravy, and low fruit and vegetable intake [46]. Moreover, the dietary inflammatory index (DII) is a population-based dietary index designed by researchers from the University of South Carolina to estimate the overall inflammatory potential of an individual’s diet [53]. Results from several observational studies have reported a strong positive association between an inflammatory diet (characterized by a high DII) and PCa [24,54,55,56,57,58,59,60,61,62,63,64,65,66]. There are only a few studies which reported no association between dietary factors and PCa [67,68,69].

Similarly, high sugar intake has been shown to increase PCa risk [70,71,72]. No consistent association between coffee and/or tea consumption has been found [73,74]. High consumption of antioxidants, such as vitamin C, vitamin E, and selenium and in combination has been shown to significantly decrease the risk of PCa [59,75,76]. The potential role of vitamin D in the aetiology of PCa remains unclear. Two meta-analyses based on nine case–control studies found a positive association between vitamin D intake and PCa (OR 1.13 (95%CI 1.07–1.19), per 100 IU/day); this positive association was reported in one meta-analysis [77,78]. However, another case–control study found no association between serum vitamin D levels and risk of PCa [79]. Folate intake has been found to have a protective role against development of PCa [80,81,82].

When looking at dietary factors in combination with other risk factors we found a synergistic association between inflammatory diet, smoking and DM. A large case–control study reported a higher risk of PCa in patients with self-reported inflammatory diet in association with long-standing DM and smoking (OR 6.03 (95%CI 3.41–10.85) and OR 4.79 (95%CI 3.00–7.65), respectively) [24].

#### 3.1.4. Nonsteroidal Anti-Inflammatory Drugs (NSAIDs)

Epidemiological studies have reported an inconsistent relationship between chronic use of aspirin and other NSAIDs. Various studied have reported a decreased risk of PCa with NSAID use [83,84,85,86], while a recent pooled analysis of 1112 cases of PCa found no association. However, there was a statistically significant positive association between NSAID use and PCa risk in diabetic patients [87].

#### 3.1.5. Infections

Numerous infectious diseases have been investigated as possible risk factors for PCa. A meta-analysis of 10 studies reported a link between hepatitis B (HBV) and hepatitis C (HCV) infection and PCa risk (OR:1.28 (95%CI 1.11–1.48) and OR: 1.21 (95%CI 1.02–1.44), respectively) [88]. The positive association between HBV and PCa risk was supported by a meta-analysis and a recent cohort study [89,90]. However, two other observational studies found no significant associations with PCa risk for HBV or HCV [91,92]. Two observational studies found a positive association between Helicobacter pylori infection and PCa [93,94]. However, various other studies found no association [95,96,97,98]. Additionally, an association has been reported between ABO blood group and populations endemic for cytotoxin associated gene A (CagA)-positive Helicobacter pylori (see below).

Several studies reported a positive link between periodontal disease and PCa [99,100]. A prospective cohort study in men reported a 64% higher risk of PCa in men with periodontal disease; a synergistic effect was seen among never smokers where a two-fold increase in PCa risk was observed [100]. A recent case–control study found a positive association with pathogenic periodontal bacteria (Porphyromonas gingivalis and Aggregatibacter actinomycetemcomitans), and a negative association with non-pathogenic oral bacteria (Phylum Fusobacteria and its genus Leptotrichia) [101,102]. This was supported by a case–control study reporting two-fold increase in PCa in individuals with high levels of antibodies to Porphyromonas gingivalis, while high levels of antibodies to common oral bacteria had a decreased risk of PCa [102]. This may be due to the influence of the humoral immune response (see below).

Other bacterial agents have been associated with increased PCa risk including an eight-fold increased risk of PCa in typhoid and paratyphoid carriers [103]. A strong positive association in patients with staphylococcus aureus bacteraemia has been reported in two cohort studies [104].

#### 3.1.6. Peptic Ulcer Disease

The association between peptic ulcer and risk of PCa has been examined by several epidemiological studies with inconclusive results. Two cohort studies found an increased risk of PCa; highest risk is for those with recent peptic ulcer prior to PCa diagnosis but with increased risk up to 19 years after diagnosis of peptic ulcer disease [105,106]. Proton pump inhibitors (PPI) have been reported as showing an increased risk of PCa (Hazard Ratio (HR) 1.34; 95%CI 1.04–1.72) in 1 study [107], while another found no significant association [108]. A pooled analysis of 10 case–control studies within the PCa Case–control Consortium (PanC4) did not support the hypothesis that peptic ulcer and its treatment impacts PCa risk [109].

#### 3.1.7. Occupational Exposures

Various studies have linked work-related exposure of various chemicals, such as pesticides, asbestos, benzene, and chlorinated hydrocarbons, to an increased risk of PCa [38,110,111,112,113,114,115]. It is thought that these substances may have genotoxic effects including altered methylation, oncogene activation, inactivation of tumour-suppressor proteins, and formation of DNA adducts [116,117]. A large case–control study reported an increased risk of PCa with self-reported exposure to pesticides (OR 1.21; 95%CI 1.02–1.44), asbestos (OR 1.54; 95%CI 1.23–1.92), benzene (OR 1.70; 95%CI 1.23–2.35), and chlorinated hydrocarbons (OR 1.63; 95%CI 1.32–2.02), these associations were stronger for men compared to women [118]. The most consistent association has been between pesticide exposure and PCa, with several epidemiological studies reporting an increased risk of PCa [38,111,114,119]. Excess risk of PCa has also been associated with ionizing radiation and silica dust [120]. The mechanisms by which these exposures may lead to PCa are not completely understood; it is thought that they exert genotoxic effects including oncogene activation and inactivation of tumour-suppressor genes [118]. No common pathways between occupational exposures and other risk factors have been found in the current review.

### 3.2. Internal Exposures

#### 3.2.1. Age

According to several epidemiological studies, most patients are older than 50 at the time of diagnosis [1,121]. The risk of developing PCa increases with age, with the highest peak occurring between 60 and 80 years of age [122]. It seldom occurs before the age of 40 years, with the median age at diagnosis of PCa being 71 years [7].

#### 3.2.2. Gender

PCa is more common in men than in women. The global incidence of PCa is 5.5 per 100,000 for men and 4.0 per 100,000 for women [1]. Specific external exposures, such as heavy smoking, high alcohol intake and occupational risk factors may have a role in this imbalance [1]. Gender-specific hormonal and reproductive factors thought to be associated with the lower risk in women are explained below.

#### 3.2.3. Family History

Familial PCa is defined in most studies as the presence of two or more first-degree relatives (FDR; parents, siblings or children) with PCa, without association with known hereditary genetic syndromes [1]. Several epidemiological studies have found an increased risk of PCa in patients with family history of PCa [121,122,123,124,125,126,127,128,129,130,131], including a recent population-based cohort which reported a three-fold increased risk of PCa [132]. The risk of PCa increases exponentially with the number of FDR involved [4]. A cohort study reported a 4.5-fold increased risk of PCa in patients with one FDR, a 6.4-fold increased risk in those with two FDRs, and up to a 32-fold risk in those with three or more FDRs [133]. Furthermore, a meta-analysis of eight studies found an increased risk of EOPC and VEOPC, defined as patients younger than 60 and 45 years, respectively [47,134]. As previously mentioned, family history of PCa in addition to DM and smoking status have been associated with a nearly 10-fold increased risk of PCa [27].

#### 3.2.4. Genetic Factors

Germline mutations in several genes have been identified to be involved in the incidence and progression of PCa, such as STK11, PRSS1, CDKN2A, BRCA1, BRCA2, MLH1, MSH2, MSH6, and PMS2 [135]. BRCA1 and BRCA2 genes have been constantly associated with breast and ovarian cancers [136]. The role of these mutations in PCa carcinogenesis has been investigated by various studies. BRCA2 gene mutations account for the highest proportion of known causes of familial PCa; they have been consistently associated with a moderate-high PCa risk, except for one study reporting 22-fold increased risk [136,137]. Additionally, a recent cohort found a positive association between germline BRCA1/2 carriers and early-onset PCa [138]. A total of three epidemiological studies found no association between BRCA1 and incidence of PCa [136,137,139]. Germline mutations in ATM, CHEK2, and PALB2 have also been associated with familial PCa [140,141,142,143]. Nevertheless, known germ-line mutations account for less than 20% of familial PCa cases.

Furthermore, specific syndromes (with germ-line mutations) have been associated with PCa incidence and progression. CDKN2A mutations have been linked with familial atypical multiple-mole melanoma [144,145], STK11 mutations to Peutz–Jeghers syndrome [146], PRSS1 mutation to patients with hereditary pancreatitis [147,148,149,150], and MLH1, MSH2, MSH6 and PMS2 mutations are all associated with Lynch syndrome [151].

#### 3.2.5. Diabetes Mellitus

The association between DM and PCa has long been recognized. Numerous studies have reported a positive association between DM and PCa risk [28,130,152,153,154]. However, whether DM is a risk factor or a manifestation of PCa, is still undefined. A meta-analysis of 22 prospective studies reported an almost two-fold increased risk of PCa in diabetic patients; among those without previously diagnosed DM, each 1 mmol/L higher than usual random plasma glucose (RPG) was associated with a HR of 1.12 (95%CI 1.43–1.63) [154]. These results on pre-diagnosed DM were supported by a nested case–control study that found an increased risk of PCa in patients with increased marker levels of hyperglycaemia (glycated haemoglobin, HbA1c) and peripheral insulin resistance (plasma insulin and proinsulin) [155]. Moreover, a recent cohort found a higher risk of PCa in diabetic patients with poor glycaemic control (HR 3.61; 95%CI 1.34–9.78) compared to those with controlled disease [153]. Family history of DM has also been linked to PCa risk, with the highest risk in patients with an offspring with DM [156]. Lastly, one cohort by Perrin et al. found a significantly increased risk of PCa in women with a history of gestational DM (OR 7.1; 95%CI 2.8–18.0) [157]. As previously mentioned, DM has been associated with a higher risk of PCa when combined with various other risk factors such as smoking, inflammatory diet, and alcohol intake.

##### New-Onset Diabetes Mellitus

New-onset DM, defined as DM diagnosis occurred less than 2 years before the PCa diagnosis, has been associated with a higher risk of PCa [158,159,160,161]. A case–control reported an increased risk of PCa associated with a duration of DM before PCa diagnosis of <2 years (OR 4.43; 95%CI 3.44–5.72), and >2 years (OR 2.11; 95%CI 1.51–2.94) [158]. Two studies supported this association, both reported that PCa risk decreased with shorter duration of DM before cancer diagnosis [37,161]. This time-course characteristic supports the hypothesis of reverse causation. DM is hypothesized of being a consequence of PCa, rather than playing a role in the development of PCa [162].

##### Late-Onset Diabetes Mellitus

Late-onset DM, or long-term DM, has been defined as diagnosis more than 2 years after PCa diagnosis. A meta-analysis including 44 studies found that long-term DM was associated with an almost two-fold increased risk of PCa [163]. Additionally, a large case–control study reported an increased risk of PCa in patients with a duration of DM of 1–4 years (OR 2.4; 95%CI 1.4–4.0) and 5–9 years (OR 2.0; 95%CI 1.2–3.4) [161]. One other pooled analysis including 1621 PCa cases found similar results [164]. The mechanism by which long-term DM may affect the incidence of PCa is hypothesized to be related to increased insulin-like growth factor 1 (IGF-1) levels, hyperglycaemia, insulin resistance and compensatory hyperinsulinemia [164].

##### Anti-Diabetic Drugs

Diabetes medications may also have an influence over the risk of PCa, although epidemiological data is still unclear. Insulin has been suggested to further increase risk of PCa in diabetic patients [165,166,167], whereas oral anti-diabetics, such as sulfonylureas, may decrease the risk [168]. These associations are supported by a meta-analysis of 15 case–control studies which reported a decreased risk of PCa among long duration of oral anti-diabetic use (OR 0.31; 95%CI 0.14–0.69), and an increased risk with insulin users (OR 5.60; 95%CI 3.75–8.35) [169]. Furthermore, a recent cohort study investigated reported no association between the use of incretin-based medications and PCa risk [170]. The association with dipeptidyl peptidase-4 inhibitor (DPP-4) drugs remains elusive [171,172,173].

#### 3.2.6. Inflammation

There is increasing evidence indicating that systemic inflammation may play a role in tumour development and progression [174,175]. A recent prospective cohort evaluating the association between serum markers of chronic inflammation (haptoglobin, C-reactive protein (CRP) and leukocytes) and risk of PCa found a positive association. This suggests the importance of inflammation as an underlying mechanism in the aetiology of PCa [176]. However, a case control study found no association between inflammatory biomarkers and PCa risk, except for high levels tumour necrosis factor-alfa (TNF-a) in women [177]. Moreover, several diseases and risk factors leading to chronic inflammation also enhance the risk of PCa [178]. These have been previously described in this study (i.e., DM, obesity, metabolic syndrome, pancreatitis, dietary pattern, smoking and alcohol consumption) (Figure 4A).

#### 3.2.7. Pancreatitis

Pancreatitis is an inflammatory condition of the pancreas with acinar cell destruction and pathologic fibrosis, which has been consistently associated with PCa incidence [127,179,180]. The major risk factors of pancreatitis include alcohol abuse, hereditary factors, and idiopathic. An increased risk of PCa has been associated with chronic pancreatitis, probably due to chronic inflammation of the pancreas [50,181,182,183,184]. However, a meta-analysis of 10 case–control studies reported a much stronger association with history of pancreatitis within 2 years of PCa diagnosis (OR 13.56; 95%CI 8.72–21.90), compared to a nearly three-fold increased risk between history >2 years before PCa diagnosis (OR 2.71; 95%CI 1.96–3.74) [185]. Similar results were reported by three other studies [186,187,188]. It is likely that this reflects a combination of reverse causation and antecedent misdiagnosis of PCa as pancreatitis, similar to early-onset DM. In addition, various studies found a stronger association with PCa risk in younger patients (<65 years), than older patients (>65 years) [181,182,183,185,187,189,190]. Two studies reported a significantly higher incidence of PCa in patients who continued to drink alcohol after diagnosis of pancreatitis compared to those who stopped drinking [188,190]. Patients who require surgery for pancreatitis are at a very high risk of developing PCa; however, early surgical intervention may play a protective role in the development of PCa from pancreatitis [130,190]. A much higher risk of cancer has been reported in patients with hereditary pancreatitis [184]. A meta-analysis suggested a 70-fold risk of PCa compared to the normal population [191].

#### 3.2.8. Immune System

The immune system has been thought to modulate the development and evolution of pancreatic carcinogenesis. PCa tissue contains multiple immunosuppressive cells, suggesting a deterioration of the immune response in the tumour microenvironment. Some studies have reported high levels of immunoglobulin G (IgG) in patients with PCa, indicating IgG as a potential diagnostic test for PCa. However, these levels cannot be used to distinguish PCa from autoimmune pancreatitis as both diseases are associated with high levels of IgG [192,193,194,195,196].

Moreover, a recent study by Sollie et al. reported an inverse association between pre-diagnostic serum levels of IgG and risk of PCa [197]. This was supported by a previously mentioned cohort study looking at the humoral immune response against oral bacteria, where high levels of antibodies had a 45% decreased risk of PCa. This may reflect that a stronger immune status could have beneficial impact on reducing the risk of PCa [102].

#### 3.2.9. Allergies

A number of epidemiological studies have reported an association between allergies and cancer [198]. Most studies have found a negative association between allergies and PCa [199,200,201,202]. One potential explanation for this protection against PCa may be through an increased “immune surveillance”. This concept hypothesizes that the immune system is capable of detecting and eliminating neoplastic cells before they are clinically visible [201]. A meta-analysis of 14 population-based studies found a decreased risk of PCa in those with a history of any allergies (Relative Risk (RR) 0.82; 95%CI 0.68–0.99); the risk reduction was stronger for allergies related to atopy (RR 0.71; 95%CI 0.64–0.80) [201]. These results were supported by another two case–control studies (192, 196). Additionally, another case–control study reported a reduction in PCa risk for history of hay fever (OR 0.68; 95%CI 0.52–0.89), dust/mould allergies (OR 0.49; 95%CI 0.31–0.78), and animal allergies (OR 0.68; 95%CI 0.46–0.99) [200]. Furthermore, the association between asthma and PCa risk has been inconsistent [202,203,204]. The majority of asthma is allergic; however, a sub-set of asthma is non-allergic (i.e., triggered by exercise). It is possible that previous studies did not differentiate between these two types of asthma, as it possible that if only allergic asthma is associated with a decreased PCa risk and all asthma was captured then misclassification may be responsible for these inconsistent findings [200]. Further interactions with other exposures were not observed in the review. Other associations between the immune system and PCa are described below in the internal exposure section.

#### 3.2.10. Hormonal and Reproductive Factors

Numerous studies have investigated potential associations between reproductive risk factors and PCa risk. A total of three studies reported a positive association between multiparity (>3 births) and PCa risk, compared to nulliparous women [205,206,207]. Regarding age at first birth, three studies found a link between an increased risk of PCa and age at first birth >30 years [55,205,208]. However, others showed an increased risk with lower age at first pregnancy [59,209]. One cohort study demonstrated an inverse association between risk of PCa with higher age at menarche [210], whereas two other studies found an increased risk of PCa associated with earlier menarche [211,212]. Concerning breastfeeding, a case–control study reported breastfeeding as a potential protective factor for PCa [38]. Furthermore, one recent cohort study reported that hormone replacement therapy (HRT) played a protective role against the development of PCa, with a stronger association for estrogen-only HRT [210]. Regarding the use of oral contraceptives there are a few studies showing inconsistent results [205,213]. No studies reported a common pathway between hormonal factors and other risk factors, in relation with PCa risk.

#### 3.2.11. Obesity

Obesity (BMI > 30 kg/m^2^) and overall increased BMI are risk factors for PCa. Various studies have evaluated the association between obesity and PCa risk in men and women, reporting an increased risk with high BMI (≥25 kg/m^2^) [130,214]. This includes a recent meta-analysis of eight studies which found a 28% increased risk of PCa in obese patients [47]. Additionally, one large cohort study found a slight increased risk of PCa in overweight and obese patients, this association was reported higher in patients who also had DM [215]. Similarly, a meta-analysis of 21 studies found an increased risk of PCa per 5 kg/m^2^ (RR 1.12; 95%CI 1.06–1.17), the risk seemed to be higher in men (RR 1.16; 95%CI 1.05–1.28) than in women (RR 1.10; 95%CI 1.02–1.19) [216]. Similar results were reported by two other meta-analysis [165,217]. Moreover, some well-known risk factors for obesity, such as decreased physical activity and dietary patterns, have also been hypothesized to play a role in PCa carcinogenesis. As previously mentioned, a Western-type diet with a high inflammatory index has been consistently associated with PCa. However, the role of physical activity (PA) remains unclear. A recent cohort study reported a decreased risk of PCa was associated with high PA, but only in individuals older than 60 years [218].

#### 3.2.12. Dyslipidaemia

Numerous studies have investigated potential links between dyslipidaemias with conflicting results. A recent cohort study reported decreased risk of PCa in patients with HDL-cholesterol (HDL) levels within the fourth quartile compared to the first; however, no associations were found for total cholesterol (TC), LDL-cholesterol (LDL), and triglyceride (TG) levels [219]. A case–control study reported a significantly increased risk of PCa in patients with high levels of TC [220]. However, two other observational studies found no association between TC and PCa risk [214,221]. Statins are commonly used to treat dyslipidaemias and have been associated with other beneficial effects including decrease in risk of various cancers [222]. A recent case–control study reported a decreased risk of PCa in statin-users [83]. However, other studies including a meta-analysis of 16 studies reported no significant associations [223,224,225,226,227].

High BMI, increased levels of TG, decreased levels of HDL and hyperglycaemia are all part of the metabolic syndrome (MetS), which has been linked to a chronic inflammatory state [228]. Two observational studies found a strong association between PCa and MetS [221,229].

#### 3.2.13. ABO Group

ABO blood group is an established risk factor for PCa and is determined by the ABO gene, located on chromosome 9q34.1 [230]. A recent meta-analysis found a positive association between overall non-O blood type and the risk of developing PCa, with the highest risk seen in type A individuals [54]. Additionally, another meta-analysis including 22 observational studies also found a positive association between ABO blood groups and the risk of developing PCa (ORs for PCa in subjects with types A, B and AB were 1.40 (95%CI 1.28–1.53), 1.19 (95%CI 1.05–1.35), and 1.29 (95%CI 1.10–1.51), respectively) [231]. According to a meta-analysis of 12 cohort studies, non-O blood groups were seen to potentiate the risk factors of other factors like smoking [232]. A synergistic association was also seen in patients with ABO group A and DM, these patients had a nearly four-fold increased risk of developing PCa (OR 3.50; 95%CI 1.66–7.41). Moreover, a meta-analysis by Risch et al. looking into the association between CagA-positive Helicobacter pylori and ABO blood group, found that in nonendemic populations, groups B and AB were associated with higher PCa risk; however, in endemic populations, B and AB groups were not associated. This association could involve gastric epithelial expression of A versus B antigens on colonization behaviours of Helicobacter pylori strains [231].

#### 3.2.14. Pancreatic Cysts

The incidence of PCa is considerably high in patients with pancreatic cystic lesions [233]. However, specific characteristics of pancreatic cysts related to the development of PCa have not yet been fully identified [233]. Most of these lesions are different from malignant cystic neoplasms; some of them have been designated as intraductal papillary mucinous neoplasm (IPMN) which are benign but have been widely reported to have malignant potential, whereas others are “simple” benign cysts. A prospective study by Tada et al. reported a 22-fold increased risk of PCa in patients with pancreatic cystic lesions [233]. This was supported by a case–control study investigating incidence and size of pancreatic cystic lesions and PCa risk; this study found a 10-fold increased risk of PCa in patients with cystic lesions of the pancreas, especially in cysts larger than 10 mm [234]. This suggests a diffuse pathological change predisposing to malignant transformation in the areas of the pancreas concealing cysts. Pancreatic cysts were not seen to be associated with other exposures described in the current study.

## 4. Discussion

Pancreatic cancer, although relatively uncommon, is mostly diagnosed in an advanced stage and remains the seventh leading cause of global cancer deaths in industrialized countries [235]. There is a large body of evidence suggesting several exposures as contributors to the risk and progression of PCa which highlights the multifactorial nature of the disease. However, the mechanisms by which these exposures interact together in order to increase the risk of PCa are still not fully known. The aim of this systematic review was to provide a comprehensive overview of the individual exposures and its interactions related with PCa carcinogenesis to ultimately present a PCa risk profile that could inform clinicians to favour early detection in PCa. In this systematic review, we observed that diseases and risk factors associated with chronic inflammation, such as DM, pancreatitis, obesity, MetS, dyslipidaemias, smoking, alcohol intake, and pro-inflammatory dietary pattern, may have a synergistic effect to further increase PCa risk. This suggests that potentially carcinogenesis in PCa is driven by inflammation processes. In addition, we found a close correlation between inflammation and the immune responses, where an enhanced immune response influenced by the inflammatory cascade may act as a protective factor for PCa [236].

This comprehensive characterization of the PCa exposome supports the multifactorial aetiology of the disease, where each of the observed exposures will account for different fractions of the overall PCa risk in the population. The prevalence of each of these individual factors in PCa patients may be relatively low as observed previously in well-established PCa risk factors. For instance, pancreatitis incidence and prevalence are relatively low and the overall frequency of pancreatic cancer in these patients is around 5%. Thus, the risk of PCa due to pancreatitis is low compared to other risk factors [7]. This ultimately indicates that fine exposure assessments combined with multimodal risk stratification tools are necessary to address early detection of pancreatic cancer.

The PCa exposure profile observed in this systematic review shows that risk of developing PCa increases with age. Regarding gender, men have been found to have an increased risk of PCa. Some studies have suggested that this may be due to men being more exposed to smoking, high alcohol intake and occupational exposures. However, various studies reported a positive association between smoking and high alcohol intake in women, and PCa risk. We did not find specific studies investigating PCa risk in relation to ethnicity or general external exposures (i.e., SES and education) in our review; however, there is a chance that these factors may play a role in the incidence and progression of PCa as several observational studies included them as confounders in their analyses. Moreover, a socioeconomic gradient has been observed previously in other cancers which may account for the interaction between these lifestyle factors and gender observed in PCa [237]. This needs to be further explored in cohort studies.

Chronic inflammation has been consistently linked with the development of PCa [178] and was found to be a key factor in the PCa exposure profile. Various individual diseases characterized by chronic low-grade inflammation are well-known risk factors for PCa (i.e., DM, obesity, MetS, and chronic pancreatitis, diet, smoking, alcohol consumption). These diseases constantly coexist with each other. Some individual risk factors for PCa, such as obesity and DM, have common risk factors among each other (i.e., PA, inflammatory diet) and overlap in the increasing of PCa risk. Some of these factors are modifiable or/and could be associated with modifiable lifestyle factors such as physical activity that changes overtime. This creates a complex interaction among exposures for PCa (as illustrated in Figure 4), that needs to be assessed overtime.

Moreover, many modifiable lifestyle factors including tobacco smoke, high intake of alcohol, and a highly inflammatory (or western-type) diet are also known to contribute to tumorigenesis of the pancreas by increasing blood levels of inflammatory cytokines, which can lead to oxidative stress, DNA damage, and ultimately, tumorigenesis [24]. In addition to these inflammatory effects, some individual exposures have other potential pathways through which they increase the risk of PCa. For instance, in hyperglycaemia and insulin resistance, insulin has growth promoting and mitogenic effects on cells. It also impacts carcinogenesis by increasing bioavailability of insulin-like growth factor (IGF), a peptide that regulates cell proliferation, differentiation, and apoptosis. Alcohol consumption leads to the production of acetaldehyde which increases the production of reactive oxygen species; these metabolites may affect exocrine and endocrine pancreatic functions and induce fibrosis [48]. Cigarette smoking results in a continuous exposure to several types of carcinogenic compounds [33]. Inflammatory diet is also characterized by food containing carcinogens (i.e., heterocyclic amines, polycyclic aromatic hydrocarbons and N-nitroso compounds), in addition to increasing inflammatory cytokines [238].

On the other hand, we found a protective effect for various factors associated with an anti-inflammatory effect, such as aspirin use and diets high in antioxidant substances (i.e., vitamin C, vitamin E, carotenoids, phenols, and flavonoids). This further supports the hypothesis that chronic inflammation is the main pathway where most exposures overlap to increase/decrease the risk of PCa. Furthermore, the immune response has also been linked to the inflammation process to modulate the development of PCa, as the onset of the inflammatory cascade may influence the nature of antibody responses. Recent epidemiological evidence has found an inverse association between PCa risk and markers of the immune response. Moreover, high levels of IgG have been found in patients with PCa and autoimmune pancreatitis, supporting the hypothesis that the inflammation and immune responses may act together in the carcinogenic process. In addition, allergies have been also described as protective factors for PCa. This is likely due to an enhanced immune surveillance, where the immune system is capable of detecting and eliminating neoplastic and preneoplastic cells before they are clinically diagnosed [201].

This systematic review provides a comprehensive qualitative summary of the published epidemiological evidence of the specific external and internal exposures associated with PCa risk. A large number of cohort, case–control and meta-analysis studies were included in this review, with a substantial number of total participants and PCa cases. However, several limitations should be noted. There is a lack of understanding of the detailed biological mechanisms underpinning these exposures; molecular and experimental studies are needed to understand the interactions between the associated exposures mentioned in this study (i.e., inflammatory pathway, humoral immune system pathway). Moreover, the association between general external exposures (i.e., SES, education, ethnicity) and the risk of PCa needs to be further investigated. Future studies exploring risk stratification tools based on the PCa exposome profile described in the current review will help improve early detection and screening in PCa.

## 5. Conclusions

PCa is an increasing cause of cancer-related mortality. A characterization of the exposome provides a template for a generalized approach to identify risk factors that contribute to the disease. Several factors included in the PCa exposure profile interact with each other through the inflammatory pathway to increase/decrease the risk of PCa. Even though some of these exposures are non-modifiable (i.e., age, male gender, family history of PCa, hereditary syndromes associated with PCa, non-O blood type, hormonal and reproductive factors, DM and pancreatitis), they are able to alert clinicians of high-risk individuals. While modifiable factors (i.e., smoking, increased alcohol consumption, inflammatory diet, occupational exposures, and obesity) included in this profile are able to be controlled in order to decrease PCa incidence. Future utilisation of these exposome factors in the development and implementation of risk stratification tools could help reduce incidence of PCa or aid in the early detection to improve survival.

## Figures and Tables

**Table 1:** Search Strategy followed in the Search engine Pubmed on 03/12/2020.

Search No.	Search Terms	Hits
Disease		
#1	“Pancreatic Neoplasms”[Mesh] OR “cancer of the pancreas” [Title/Abstract] OR “pancreatic cancer” [Title/Abstract] OR “pancreatic tumour” [Title/Abstract]	69,527
Risk Factors		
#2	(“Risk Factors”[Mesh] OR “Biomarkers”[Mesh] OR “Exposome”[Mesh] OR “Causality”[Mesh] AND “etiology” [Subheading])	756,580
#3	#1 AND #2	5872
Exclusions		
#4	“Animals”[Mesh] NOT “Humans”[Mesh]	3,503,479
#5	“Adult”[Mesh]	5,373,576
Total		
#6	(#3 NOT #4) AND #5	3390
Full text (#6)		3029

**Table 2:** Summary of the exposures that define the PCa exposure profile. Strength of association is defined by the number of studies reporting on the association, the range of the hazard ratio/odds ratio/relative risk/standardized incidence ratio reported in each study, and the statistical significance.

Exposure	Studies	Summary of Findings
**Specific External Exposures**		
Smoking	9	Strong increased risk of PCa in current and former smokers. Strong increased risk with higher smoking intensity. Strong increased risk of PCa in ETS.
Alcohol Consumption	10	Strong increased risk of PCa in heavy drinkers *.
Dietary Patterns	25	Strong decreased risk of PCa in highest category of healthy pattern. Strong increased risk of PCa in highest category of western-type diet. Strong increased risk of PCa in highest category of DII score. Strong increased risk of PCa with soft drink/juice consumption. Intermediate decreased risk of PCa in dietary antioxidants. Intermediate increased risk of PCa with Vitamin D.
NSAIDs	5	Weak decreased risk of PCa with NSAIDs.
Infectious Agents	18	Strong increased risk of PCa with HBV infection. No clear associations with other infectious agents. Strong increased risk of PCa in patients with pathogenic oral bacteria. Strong decreased risk of PCa in patients with non-pathogenic oral bacteria.
Peptic Ulcer Disease	3	Weak increased risk of PCa in patients with peptic ulcer disease.
Occupational Exposures	6	Strong increased risk of PCa with pesticide exposure. No clear associations for other exposures.
Allergies	6	Strong decreased risk of PCa with allergies.
**Internal exposures**		
ABO group	6	Strong increased risk of PCa in patients with non-O blood groups, with the highest risk in type A patients.
Diabetes Mellitus	18	Strong increased risk of PCa in patients with DM, with higher risk for new-onset DM. Intermediate increased risk of PCa in patients using insulin. Intermediate decreased risk of PCa in patients with long duration oral anti-diabetics.
Pancreatitis	12	Strong increased risk of PCa in patients with history of pancreatitis, with stronger risk for new-onset pancreatitis.
Hormonal and Reproductive Factors	10	Intermediate decreased risk with multiparity >3 births. Weak associations for all other reproductive factors.
Obesity	8	Strong increased risk of PCa in patients with high BMI (overweight/obesity).
Physical Activity	1	Weak decreased risk of PCa in patients with high physical activity.
Metabolic Syndrome	2	Strong increased risk of PCa with metabolic syndrome.
Dyslipidaemias	2	Weak decreased risk of PCa with high HDL-cholesterol levels. Weak increased risk of PCa with high total cholesterol levels.
Statin Use	6	No associations.
Family History of PCa	12	Strong increased risk of PCa with FH of PCa.
Inflammatory Markers	2	Strong increased risk of PCa with high inflammatory markers.
Genetic Factors	12	Strong increased risk of PCa with various genetic mutations.
Cystic Lesions	2	Strong increased risk of PCa with pancreatic cystic lesions.

* Heavy drinking definitions varied according to each study. PCa, pancreatic cancer; NSAID, non-steroidal anti-inflammatory drugs; FH, family history; HR, hazard ratio; OR, odds ratio; RR, relative risk.

## Data Availability

The data presented in this study are available on request from the corresponding author.

## Supplementary Materials

The following are available online at https://www.mdpi.com/article/10.3390/jcm10081665/s1, Table S1: contains ecological, observational and interventional studies addressing specific external exposures in PCa, Table S2: contains ecological, observational and interventional studies addressing internal exposures in PCa.

## References

[1] Rawla P., Sunkara T., Gaduputi V. (2019). Epidemiology of Pancreatic Cancer: Global Trends, Etiology and Risk Factors. World J. Oncol..

[2] Sung H., Ferlay J., Siegel R.L., Laversanne M., Soerjomataram I., Jemal A., Bray F. (2021). Global cancer statistics 2020: GLOBOCAN es-timates of incidence and mortality worldwide for 36 cancers in 185 countries. Ca Cancer J. Clin..

[3] Mizrahi J.D., Surana R., Valle J.W., Shroff R.T. (2020). Pancreatic cancer. Lancet.

[4] Yeo T.P. (2015). Demographics, Epidemiology, and Inheritance of Pancreatic Ductal Adenocarcinoma. Semin. Oncol..

[5] Kong B., Michalski C.W., Erkan M., Friess H., Kleeff J. (2011). From tissue turnover to the cell of origin for pancreatic cancer. Nat. Rev. Gastroenterol. Hepatol..

[6] PanCan Types of Pancreatic Cancer 2020. https://www.pancan.org/facing-pancreatic-cancer/about-pancreatic-cancer/types-of-pancreatic-cancer/.

[7] Midha S., Chawla S., Garg P.K. (2016). Modifiable and non-modifiable risk factors for pancreatic cancer: A review. Cancer Lett..

[8] Mario C., Marilisa F., Kryssia I.R.C., Pellegrino C., Ginevra C., Chiara M., Alberto B., Antonio N., Gioacchino L., Tiziana M. (2018). Epidemiology and risk factors of pancreatic cancer. Acta Biomed..

[9] Guertin K.A., Freedman N.D., Loftfield E., Stolzenberg-Solomon R.Z., Graubard B.I., Sinha R. (2015). A prospective study of coffee intake and pancreatic cancer: Results from the NIH-AARP Diet and Health Study. Br. J. Cancer.

[10] Neoptolemos J.P., Palmer D.H., Ghaneh P., Psarelli E.E., Valle J.W., Halloran C.M., Faluyi O., O’Reilly D.A., Cunningham D., Wadsley J. (2017). Comparison of adjuvant gemcitabine and capecitabine with gemcitabine monotherapy in patients with resected pancreatic cancer (ESPAC-4): A multicentre, open-label, randomised, phase 3 trial. Lancet.

[11] Zheng J., Guinter M.A., Merchant A.T., Wirth M.D., Zhang J., Stolzenberg-Solomon R.Z., Steck S.E. (2017). Dietary patterns and risk of pancreatic cancer: A systematic review. Nutr. Rev..

[12] Bosetti C., Bertuccio P., Negri E., La Vecchia C., Zeegers M.P., Boffetta P. (2012). Pancreatic cancer: Overview of descriptive epidemi-ology. Mol. Carcinog..

[13] Wild C.P. (2005). Complementing the genome with an “exposome”: The outstanding challenge of environmental exposure meas-urement in molecular epidemiology. Cancer Epidemiol. Biomark. Prev..

[14] Vineis P., Robinson O., Chadeau-Hyam M., Dehghan A., Mudway I., Dagnino S. (2020). What is new in the exposome?. Environ. Int..

[15] Vineis P., Chadeau-Hyam M., Gmuender H., Gulliver J., Herceg Z., Kleinjans J., Kogevinas M., Kyrtopoulos S., Nieuwenhuijsen M., Phillips D. (2017). The exposome in practice: Design of the EXPOsOMICS project. Int. J. Hyg. Environ. Health.

[16] Wild C.P. (2012). The exposome: From concept to utility. Int. J. Epidemiol..

[17] Mohammed S.H., Habtewold T.D., Birhanu M.M., Sissay T.A., Tegegne B.S., Abuzerr S., Esmaillzadeh A. (2019). Neighbourhood socioeconomic status and overweight/obesity: A systematic review and meta-analysis of epidemiological studies. Bmj Open.

[18] Hsing A.W., Sakoda L.C., Chua S.C. (2007). Obesity, metabolic syndrome, and prostate cancer. Am. J. Clin. Nutr..

[19] Robinson O., Vrijheid M. (2015). The Pregnancy Exposome. Curr. Environ. Health Rep..

[20] Daiber A., Lelieveld J., Steven S., Oelze M., Kröller-Schön S., Sørensen M., Münzel T. (2019). The “exposome” concept—how environmental risk factors influence cardiovascular health. Acta Biochim. Pol..

[21] Dréno B., Bettoli V., Araviiskaia E., Viera M.S., Bouloc A. (2018). The influence of exposome on acne. J. Eur. Acad. Dermatol. Venereol..

[22] Pham A., Forsmark C. (2018). Chronic pancreatitis: Review and update of etiology, risk factors, and management. F1000Research.

[23] Moher D., Liberati A., Tetzlaff J., Altman D.G., The PRISMA Group (2009). Preferred reporting items for systematic reviews and meta-analyses: The PRISMA statement. PLoS Med..

[24] Antwi S.O., Oberg A.L., Shivappa N., Bamlet W.R., Chaffee K.G., Steck S.E., Hebert J.R., Petersen G.M. (2016). Pancreatic cancer: Associations of inflammatory potential of diet, cigarette smoking and long-standing diabetes. Carcinogenesis.

[25] Hassan M.M., Abbruzzese J.L., Bondy M.L., Wolff R.A., Vauthey J.-N., Pisters P.W., Evans D.B., Khan R., Lenzi R., Jiao L. (2007). Passive smoking and the use of noncigarette tobacco products in association with risk for pancreatic cancer: A case-control study. Cancer.

[26] Korc M., Jeon C.Y., Edderkaoui M., Pandol S.J., Petrov M.S. (2017). Tobacco and alcohol as risk factors for pancreatic cancer. Best Pr. Res. Clin. Gastroenterol..

[27] Matsubayashi H., Maeda A., Kanemoto H., Uesaka K., Yamazaki K., Hironaka S., Miyagi Y., Ikehara H., Ono H., Klein A. (2011). Risk factors of familial pancreatic cancer in Japan: Current smoking and recent onset of diabetes. Pancreas.

[28] Molina-Montes E., Gomez-Rubio P., Márquez M., Rava M., Löhr M., Michalski C.W., Molero X., Farré A., Perea J., Greenhalf W. (2018). Risk of pancreatic cancer associated with family history of cancer and other medical conditions by accounting for smoking among relatives. Int. J. Epidemiol..

[29] Schulte A., Pandeya N., Tran B., Fawcett J., Fritschi L., Risch H.A., Webb P.M., Whiteman D.C., Neale R.E. (2014). Cigarette smoking and pancreatic cancer risk: More to the story than just pack-years. Eur. J. Cancer.

[30] Vrieling A., Bueno-de-Mesquita H.B., Boshuizen H.C., Michaud D.S., Severinsen M.T., Overvad K., Olsen A., Tjønneland A., Clavel-Chapelon F., Boutron-Ruault M.C. (2010). Cigarette smoking, en-vironmental tobacco smoke exposure and pancreatic cancer risk in the European Prospective Investigation into Cancer and Nu-trition. Int. J. Cancer.

[31] Kuzmickiene I., Everatt R., Virviciute D., Tamosiunas A., Radisauskas R., Reklaitiene R., Milinaviciene E. (2013). Smoking and other risk factors for pancreatic cancer: A cohort study in men in Lithuania. Cancer Epidemiol..

[32] Wang Y., Duan H., Yang X., Guo J. (2014). Cigarette smoking and the risk of pancreatic cancer: A case–control study. Med Oncol..

[33] Heinen M.M., Verhage B.A., Goldbohm R.A., Brandt P.A.V.D. (2010). Active and Passive Smoking and the Risk of Pancreatic Cancer in the Netherlands Cohort Study. Cancer Epidemiol. Biomark. Prev..

[34] Brand R.E., Greer J.B., Zolotarevsky E., Brand R., Du H., Simeone D., Zisman A., Gorchow A., Lee S. (Connie), Roy H.K. (2009). Pancreatic Cancer Patients Who Smoke and Drink Are Diagnosed at Younger Ages. Clin. Gastroenterol. Hepatol..

[35] Muscat J.E., Stellman S.D., Hoffmann D., Wynder E.L. (1997). Smoking and pancreatic cancer in men and women. Cancer Epidemiol. Biomark. Prev..

[36] Nilsen T.I.L., Vatten L.J. (2000). A prospective study of lifestyle factors and the risk of pancreatic cancer in Nord-Trøndelag, Norway. Cancer Causes Control..

[37] Hassan M.M., Bondy M.L., Wolff R.A., Abbruzzese J.L., Vauthey J.-N., Pisters P.W., Evans D.B., Khan R., Chou T.-H., Lenzi R. (2007). Risk Factors for Pancreatic Cancer: Case-Control Study. Am. J. Gastroenterol..

[38] Lo A.-C., Soliman A.S., El-Ghawalby N., Abdel-Wahab M., Fathy O., Khaled H.M., Omar S., Hamilton S.R., Greenson J.K., Abbruzzese J.L. (2007). Lifestyle, Occupational, and Reproductive Factors in Relation to Pancreatic Cancer Risk. Pancreas.

[39] Chuang S.-C., Gallo V., Michaud M., Overvad K., Tjønneland A., Clavel-Chapelon F., Romieu I., Straif K., Palli D., Pala V. (2011). Exposure to environmental tobacco smoke in childhood and incidence of cancer in adulthood in never smokers in the European prospective investigation into cancer and nutrition. Cancer Causes Control..

[40] Gallicchio L., Kouzis A., Genkinger J.M., Burke A.E., Hoffman S.C., Diener-West M., Helzlsouer K.J., Comstock G.W., Alberg A.J. (2006). Active cigarette smoking, household passive smoke exposure, and the risk of developing pancreatic cancer. Prev. Med..

[41] Villeneuve P.J., Johnson K.C., Mao Y., Hanley A.J., Paulse B., Dewar R., Dryer D., Kreiger N., Kliewer E., Robson D. (2004). Environmental tobacco smoke and the risk of pancreatic cancer: Findings from a Canadian population-based case-control study. Can. J. Public Health.

[42] Capurso G., Falconi M., Panzuto F., Rinzivillo M., Boninsegna L., Bettini R., Corleto V., Borgia P., Pederzoli P., Scarpa A. (2009). Risk factors for sporadic pancreatic endocrine tumors: A case-control study of prospectively evaluated patients. Am. J. Gastroenterol..

[43] Genkinger J.M., Spiegelman D., Anderson K.E., Bergkvist L., Bernstein L., Brandt P.A.V.D., English D.R., Freudenheim J.L., Fuchs C.S., Giles G.G. (2009). Alcohol Intake and Pancreatic Cancer Risk: A Pooled Analysis of Fourteen Cohort Studies. Cancer Epidemiol. Biomark. Prev..

[44] Gupta S., Wang F., Holly E.A., Bracci P.M. (2010). Risk of pancreatic cancer by alcohol dose, duration, and pattern of consumption, including binge drinking: A population-based study. Cancer Causes Control..

[45] Klein A.P., Lindstroem S., Mendelsohn J.B., Steplowski E., Arslan A.A., Bueno-De-Mesquita H.B., Fuchs C.S., Gallinger S., Gross M., Helzlsouer K. (2013). An Absolute Risk Model to Identify Individuals at Elevated Risk for Pancreatic Cancer in the General Population. PLoS ONE.

[46] Lu X.H., Wang L., Li H., Qian J.M., Deng R.X., Zhou L. (2006). Establishment of risk model for pancreatic cancer in Chinese Han popu-lation. World J. Gastroenterol..

[47] McWilliams R.R., Maisonneuve P., Bamlet W.R., Petersen G.M., Li D., Risch H., Yu H., Fontham E.T., Luckett B., Bosetti C. (2016). Risk Factors for Early-Onset and Very-Early-Onset Pancreatic Adenocarcinoma: A Pancreatic Cancer Case-Control Consortium (PanC4) Analysis. Pancreas.

[48] Naudin S., Li K., Jaouen T., Assi N., Kyrø C., Tjønneland A., Overvad K., Boutron-Ruault M.-C., Rebours V., Védie A.-L. (2018). Lifetime and baseline alcohol intakes and risk of pancreatic cancer in the European Prospective Investigation into Cancer and Nutrition study. Int. J. Cancer.

[49] Miyasaka K., Kawanami T., Shimokata H., Ohta S., Funakoshi A. (2005). Inactive Aldehyde Dehydrogenase-2 Increased the Risk of Pancreatic Cancer Among Smokers in a Japanese Male Population. Pancreas.

[50] Talamini G., Bassi C., Falconi M., Sartori N., Salvia R., Rigo L., Castagnini A., Di Francesco V., Frulloni L., Bovo P. (1999). Alcohol and smoking as risk factors in chronic pancreatitis and pancreatic cancer. Dig. Dis. Sci..

[51] Bouchardy C., Clavel F., La Vecchia C., Raymond L., Boyle P. (1990). Alcohol, beer and cancer of the pancreas. Int. J. Cancer.

[52] Falk R.T., Pickle L.W., Fontham E.T., Correa P., Fraumeni J.F. (1988). Life-style risk factors for pancreatic cancer in louisiana: A case-control study. Am. J. Epidemiol..

[53] Shivappa N., Steck S.E., Hurley T.G., Hussey J.R., Hebert J.R. (2014). Designing and developing a literature-derived, population-based dietary inflammatory index. Public Health Nutr..

[54] Antwi S.O., Bamlet W.R., Pedersen K.S., Chaffee K.G., Risch H.A., Shivappa N., Steck S.E., Anderson K.E., Bracci P.M., Polesel J. (2018). Pancreatic cancer risk is modulated by in-flammatory potential of diet and ABO genotype: A consortia-based evaluation and replication study. Carcinogenesis.

[55] Larsson S.C., Wolk A. (2012). Red and processed meat consumption and risk of pancreatic cancer: Meta-analysis of prospective studies. Br. J. Cancer.

[56] Shivappa N., Bosetti C., Zucchetto A., Serraino D., La Vecchia C., Hebert J.R. (2014). Dietary inflammatory index and risk of pancreatic cancer in an Italian case-control study. Br. J. Nutr..

[57] Bao Y., Nimptsch K., Wolpin B.M., Michaud D.S., Brand-Miller J.C., Willett W.C., Giovannucci E., Fuchs C.S. (2011). Dietary insulin load, dietary insulin index, and risk of pancreatic cancer. Am. J. Clin. Nutr..

[58] Lin Y., Tamakoshi A., Hayakawa T., Naruse S., Kitagawa M., Ohno Y. (2005). Nutritional factors and risk of pancreatic cancer: A pop-ulation-based case-control study based on direct interview in Japan. J. Gastroenterol..

[59] Ji B.T., Chow W.H., Gridley G., McLaughlin J.K., Dai Q., Wacholder S., Hatch M.C., Gao Y.T., Fraumeni J.F. (1995). Dietary factors and the risk of pancreatic cancer: A case-control study in Shanghai China. Cancer Epidemiol. Biomark. Prev..

[60] Wang J., Zhang W., Sun L., Yu H., Ni Q.-X., Risch H.A., Gao Y.-T. (2013). Dietary energy density is positively associated with risk of pancreatic cancer in urban Shanghai Chinese. J. Nutr..

[61] Farrow D.C., Davis S. (1990). Diet and the risk of pancreatic cancer in men. Am. J. Epidemiol..

[62] Soler M., Chatenoud L., La Vecchia C., Franceschi S., Negri E. (1998). Diet, alcohol, coffee and pancreatic cancer: Final results from an Italian study. Eur. J. Cancer Prev..

[63] Anderson K.E., Mongin S.J., Sinha R., Stolzenberg-Solomon R., Gross M.D., Ziegler R.G., Mabie J.E., Risch A., Kazin S.S., Church T.R. (2012). Pancreatic cancer risk: Associations with meat-derived carcinogen intake in the Prostate, Lung, Colorectal, and Ovarian Cancer Screening Trial (PLCO) cohort. Mol. Carcinog..

[64] Jayedi A., Emadi A., Shab-Bidar S. (2018). Dietary Inflammatory Index and Site-Specific Cancer Risk: A Systematic Review and Dose-Response Meta-Analysis. Adv. Nutr..

[65] Lucenteforte E., Talamini R., Bosetti C., Polesel J., Franceschi S., Serraino D., Negri E., La Vecchia C. (2010). Macronutrients, fatty acids, cholesterol and pancreatic cancer. Eur. J. Cancer.

[66] Ruan Y., Poirier A.E., Hebert L.A., Grevers X., Walter S.D., Villeneuve P.J., Brenner D.R., Friedenreich C.M. (2019). Estimates of the current and future burden of cancer attributable to red and processed meat consumption in Canada. Prev. Med..

[67] Zheng J., Merchant A.T., Wirth M.D., Zhang J., Antwi S.O., Shoaibi A., Shivappa N., Stolzenberg-Solomon R.Z., Hebert J.R., Steck S.E. (2018). Inflammatory potential of diet and risk of pancreatic cancer in the Prostate, Lung, Colorectal and Ovarian (PLCO) Cancer Screening Trial. Int. J. Cancer.

[68] Zheng J., Wirth M.D., Merchant A.T., Zhang J., Shivappa N., Stolzenberg-Solomon R.Z., Hebert J.R., Steck S.E. (2019). Inflammatory Potential of Diet, Inflammation-Related Lifestyle Factors, and Risk of Pancreatic Cancer: Results from the NIH-AARP Diet and Health Study. Cancer Epidemiol. Biomark. Prev..

[69] Åsli L.A., Braaten T., Olsen A., Tjønneland A., Overvad K., Nilsson L.M., Renström F., Lund E., Skeie G. (2018). Potato consumption and risk of pancreatic cancer in the HELGA cohort. Br. J. Nutr..

[70] Mueller N.T., Odegaard A., Anderson K., Yuan J.-M., Gross M., Koh W.-P., Pereira M.A. (2010). Soft Drink and Juice Consumption and Risk of Pancreatic Cancer: The Singapore Chinese Health Study. Cancer Epidemiol. Biomark. Prev..

[71] Schernhammer E.S., Hu F.B., Giovannucci E., Michaud D.S., Colditz G.A., Stampfer M.J., Fuchs C.S. (2005). Sugar-sweetened soft drink con-sumption and risk of pancreatic cancer in two prospective cohorts. Cancer Epidemiol. Biomark. Prev..

[72] Nöthlings U., Murphy S.P., Wilkens L.R., Henderson B.E., Kolonel L.N. (2007). Dietary glycemic load, added sugars, and carbohydrates as risk factors for pancreatic cancer: The Multiethnic Cohort Study. Am. J. Clin. Nutr..

[73] Luo J., Inoue M., Iwasaki M., Sasazuki S., Otani T., Ye W., Tsugane S. (2007). Green tea and coffee intake and risk of pancreatic cancer in a large-scale, population-based cohort study in Japan (JPHC study). Eur. J. Cancer Prev..

[74] Turati F., Galeone C., Talamini R., Franceschi S., Manzari M., Gallino G., Polesel J., La Vecchia C., Tavani A. (2011). Coffee, decaffeinated coffee, tea, and pancreatic cancer risk: A pooled-analysis of two Italian case-control studies. Eur. J. Cancer Prev..

[75] Banim P.J.R., Luben R., McTaggart A., Welch A., Wareham N., Khaw K.-T., Hart A.R. (2012). Dietary antioxidants and the aetiology of pancreatic cancer: A cohort study using data from food diaries and biomarkers. Gut.

[76] Lucas A.L., Bosetti C., Boffetta P., Negri E., Tavani A., Serafini M., Polesel J., Serraino D., La Vecchia C., Rossi M. (2016). Dietary total antioxidant capacity and pancreatic cancer risk: An Italian case–control study. Br. J. Cancer.

[77] Waterhouse M., Risch H.A., Bosetti C., Anderson K.E., Petersen G.M., Bamlet W.R., Cotterchio M., Cleary S.P., Ibiebele T.I., La Vecchia C. (2015). Vitamin D and pancreatic cancer: A pooled analysis from the Pancreatic Cancer Case–Control Consortium. Ann. Oncol..

[78] Stolzenberg-Solomon R.Z., Jacobs E.J., Arslan A.A., Qi D., Patel A.V., Helzlsouer K.J., Weinstein S.J., McCullough M.L., Purdue M.P., Shu X.-O. (2010). Circulating 25-Hydroxyvitamin D and Risk of Pancreatic Cancer: Cohort Consortium Vitamin D Pooling Project of Rarer Cancers. Am. J. Epidemiol..

[79] Stolzenberg-Solomon R.Z., Hayes R.B., Horst R.L., Anderson K.E., Hollis B.W., Silverman D.T. (2009). Serum vitamin D and risk of pan-creatic cancer in the prostate, lung, colorectal, and ovarian screening trial. Cancer Res..

[80] Chuang S.-C., Stolzenberg-Solomon R., Ueland P.M., Vollset S.E., Midttun Ø., Olsen A., Tjønneland A., Overvad K., Boutron-Ruault M.-C., Morois S. (2011). A U-shaped relationship between plasma folate and pancreatic cancer risk in the European Prospective Investigation into Cancer and Nutrition. Eur. J. Cancer.

[81] Gong Z., Holly E.A., Bracci P.M. (2009). Intake of folate, vitamins B6, B12 and methionine and risk of pancreatic cancer in a large population-based case–control study. Cancer Causes Control..

[82] Stolzenberg-Solomon R.Z., Albanes D., Nieto F.J., Hartman T.J., Tangrea J.A., Rautalahti M., Sehlub J., Viramo J., Taylor P.R. (1999). Pancreatic cancer risk and nu-trition-related methyl-group availability indicators in male smokers. J. Natl. Cancer Inst..

[83] Archibugi L., Piciucchi M., Stigliano S., Valente R., Zerboni G., Barucca V., Milella M., Maisonneuve P., Fave G.D., Capurso G. (2017). Exclusive and Combined Use of Statins and Aspirin and the Risk of Pancreatic Cancer: A Case-Control Study. Sci. Rep..

[84] Bradley M.C., Hughes C.M., Cantwell M.M., Napolitano G., Murray L.J. (2010). Non-steroidal anti-inflammatory drugs and pancreatic cancer risk: A nested case–control study. Br. J. Cancer.

[85] Coogan P.F., Rosenberg L., Palmer J.R., Strom B.L., Zauber A.G., Stolley P.D., Shapiro S. (2000). Nonsteroidal anti-inflammatory drugs and risk of digestive cancers at sites other than the large bowel. Cancer Epidemiol. Biomark. Prev..

[86] Schernhammer E.S., Kang J.-H., Chan A.T., Michaud D.S., Skinner H.G., Giovannucci E., Colditz G.A., Fuchs C.S. (2004). A Prospective Study of Aspirin Use and the Risk of Pancreatic Cancer in Women. J. Natl. Cancer Inst..

[87] Khalaf N., Yuan C., Hamada T., Cao Y., Babic A., Morales-Oyarvide V., Kraft P., Ng K., Giovannucci E., Ogino S. (2018). Regular Use of Aspirin or Non-Aspirin Nonsteroidal Anti-Inflammatory Drugs Is Not Associated With Risk of Incident Pancreatic Cancer in Two Large Cohort Studies. Gastroenterology.

[88] Xing S., Li Z.-W., Tian Y.-F., Zhang L.-M., Li M.-Q., Zhou P. (2013). Chronic hepatitis virus infection increases the risk of pancreatic cancer: A meta-analysis. Hepatobiliary Pancreat. Dis. Int..

[89] Wang Y., Yang S., Song F., Cao S., Yin X., Xie J., Tu X., Xu J., Xu X., Dong X. (2013). Hepatitis B virus status and the risk of pancreatic cancer: A meta-analysis. Eur. J. Cancer Prev..

[90] Song C., Lv J., Liu Y., Chen J.G., Ge Z., Zhu J., Dai J., Du L.-B., Yu C., Guo Y. (2019). Associations Between Hepatitis B Virus Infection and Risk of All Cancer Types. Jama Netw. Open.

[91] Chang M.-C., Chen C.-H., Liang J.-D., Tien Y.-W., Hsu C., Wong J.-M., Chang Y.-T. (2014). Hepatitis B and C viruses are not risks for pancreatic adenocarcinoma. World J. Gastroenterol..

[92] Tang J., Sharma R., Lamerato L., Sheehan M., Krajenta R., Gordon S.C. (2014). Is previous exposure to hepatitis B a risk factor for pan-creatic cancer or hepatocellular carcinoma?. J. Clin. Gastroenterol..

[93] Risch H.A., Lu L., Kidd M.S., Wang J., Zhang W., Ni Q., Gao Y.-T., Yu H. (2014). Helicobacter pylori Seropositivities and Risk of Pancreatic Carcinoma. Cancer Epidemiol. Biomark. Prev..

[94] Ai F., Hua X., Liu Y., Lin J., Feng Z. (2015). Preliminary Study of Pancreatic Cancer Associated with Helicobacter pylori Infection. Cell Biophys..

[95] De Martel C., Llosa A.E., Friedmana G.D., Vogelman J.H., Orentreich N., Stolzenberg-Solomon R.Z., Parsonnet J. (2008). Helicobacter pylori infection and development of pancreatic cancer. Cancer Epidemiol. Biomark. Prev..

[96] Hirabayashi M., Inoue M., Sawada N., Saito E., Abe S.K., Hidaka A., Iwasaki M., Yamaji T., Shimazu T., Tsugane S. (2019). Helicobacter pylori infection, atrophic gastritis, and risk of pancreatic cancer: A population-based cohort study in a large Japanese population: The JPHC Study. Sci. Rep..

[97] Lindkvist B., Johansen D., Borgstrom A., Manjer J. (2008). A prospective study of Helicobacter pylori in relation to the risk for pan-creatic cancer. BMC Cancer.

[98] Chen X.-Z., Schöttker B., Castro F.A., Chen H., Zhang Y., Holleczek B., Brenner H. (2016). Association of helicobacter pylori infection and chronic atrophic gastritis with risk of colonic, pancreatic and gastric cancer: A ten-year follow-up of the ESTHER cohort study. Oncotarget.

[99] Gerlovin H., Michaud D.S., Cozier Y.C., Palmer J.R. (2019). Oral Health in Relation to Pancreatic Cancer Risk in African American Women. Cancer Epidemiol. Biomark. Prev..

[100] Michaud D.S., Joshipura K., Giovannucci E., Fuchs C.S. (2007). A Prospective Study of Periodontal Disease and Pancreatic Cancer in US Male Health Professionals. J. Natl. Cancer Inst..

[101] Fan X., Alekseyenko A.V., Wu J., Peters B.A., Jacobs E.J., Gapstur S.M., Purdue M.P., Abnet C.C., Stolzenberg-Solomon R., Miller G. (2018). Human oral microbiome and prospective risk for pancreatic cancer: A population-based nested case-control study. Gut.

[102] Michaud D.S., Izard J., Wilhelm-Benartzi C.S., You D.-H., Grote V.A., Tjønneland A., Dahm C.C., Overvad K., Jenab M., Fedirko V. (2013). Plasma antibodies to oral bacteria and risk of pancreatic cancer in a large European prospective cohort study. Gut.

[103] Caygill C., Hill M., Braddick M., Sharp J. (1994). Cancer mortality in chronic typhoid and paratyphoid carriers. Lancet.

[104] Gotland N., Uhre M.L., Sandholdt H., Mejer N., Lundbo L.F., Petersen A., Larsen A.R., Benfield T. (2020). Increased risk of incident primary cancer after Staphylococcus aureus bacteremia: A matched cohort study. Medicine.

[105] Bao Y., Spiegelman D., Li R., Giovannucci E., Fuchs C.S., Michaud D.S. (2010). History of Peptic Ulcer Disease and Pancreatic Cancer Risk in Men. Gastroenterology.

[106] Luo J., Nordenvall C., Nyrén O., Adami H.-O., Permert J., Ye W. (2006). The risk of pancreatic cancer in patients with gastric or duodenal ulcer disease. Int. J. Cancer.

[107] Hwang I.C., Chang J., Park S.M. (2018). Association between proton pump inhibitor use and the risk of pancreatic cancer: A Korean nationwide cohort study. PLoS ONE.

[108] Hicks B., Friis S., Pottegård A. (2018). Use of proton pump inhibitors and risk of pancreatic cancer. Pharmacoepidemiol. Drug Saf..

[109] Bosetti C., Lucenteforte E., Bracci P.M., Negri E., Neale R.E., Risch H.A., Olson S.H., Gallinger S., Miller A.B., Bueno-De-Mesquita H.B. (2013). Ulcer, gastric surgery and pancreatic cancer risk: An analysis from the International Pancreatic Cancer Case–Control Consortium (PanC4). Ann. Oncol..

[110] Alguacil J., Kauppinen T., Porta M., Partanen T., Malats N., Kogevinas M., Benavides F.G., Obiols J., Bernal F., Rifà J. (2000). Risk of pancreatic cancer and occupational exposures in Spain. Ann. Occup. Hyg..

[111] Andreotti G., Freeman L.B., Hou L., Coble J., Rusiecki J., Hoppin J., Silverman D., Alavanja M. (2008). Abstract A120: Agricultural pesticide use and pancreatic cancer risk in the agricultural health study cohort. Int J Cancer..

[112] Andreotti G., Silverman D.T. (2011). Occupational risk factors and pancreatic cancer: A review of recent findings. Mol. Carcinog..

[113] Ojajärvi A., Partanen T., Ahlbom A., Hakulinen T., Kauppinen T., Weiderpass E., Wesseling C. (2007). Estimating the relative risk of pancreatic cancer associated with exposure agents in job title data in a hierarchical Bayesian meta-analysis. Scand. J. Work. Environ. Health.

[114] Hoppin J.A., Tolbert P.E., Holly E.A., Brock J.W., Korrick S.A., Altshul L.M., Zhang R.H., Bracci P.M., Burse V.W., Needham L.L. (2000). Pancreatic cancer and serum organochlorine levels. Cancer Epidemiol. Biomark. Prev..

[115] Ji B.-T., ScD D.T.S., Stewart P.A., Blair A., Swanson G.M., Baris D., Greenberg R.S., Hayes R.B., Brown L.M., Lillemoe K.D. (2001). Occupational exposure to pesticides and pancreatic cancer. Am. J. Ind. Med..

[116] Lowenfels A.B., Maisonneuve P. (2006). Epidemiology and risk factors for pancreatic cancer. Best Pr. Res. Clin. Gastroenterol..

[117] Wogan G.N., Hecht S.S., Felton J.S., Conney A.H., Loeb L.A. (2004). Environmental and chemical carcinogenesis. Semin. Cancer Biol..

[118] Antwi S.O., Eckert E.C., Sabaque C.V., Leof E.R., Hawthorne K.M., Bamlet W.R., Chaffee K.G., Oberg A.L., Petersen G.M. (2015). Exposure to environmental chemicals and heavy metals, and risk of pancreatic cancer. Cancer Causes Control..

[119] Kachuri L., Harris M.A., MacLeod J.S., Tjepkema M., Peters P.A., Demers P.A. (2017). Cancer risks in a population-based study of 70,570 agricultural workers: Results from the Canadian census health and Environment cohort (CanCHEC). BMC Cancer.

[120] Kauppinen T., Partanen T., Degerth R., Ojajdrvi A. (1995). Pancreatic Cancer and Occupational Exposures. Epidemiol..

[121] Review SCS SEER Cancer Statistics Review 1975-2017 National Cancer Institue, Bethesda, MD2018. https://seer.cancer.gov/csr/1975_2017/browse_csr.php.

[122] Ilic M., Ilic I. (2016). Epidemiology of pancreatic cancer. World J. Gastroenterol..

[123] Fernandez E., La Vecchia C., D’Avanzo B., Negri E., Franceschi S. (1994). Family history and the risk of liver, gallbladder, and pancreatic cancer. Cancer Epidemiol. Biomark. Prev..

[124] Catts Z.A.-K., Baig M.K., Milewski B., Keywan C., Guarino M., Petrelli N. (2016). Statewide Retrospective Review of Familial Pancreatic Cancer in Delaware, and Frequency of Genetic Mutations in Pancreatic Cancer Kindreds. Ann. Surg. Oncol..

[125] Zhan H.-X., Cong L., Zhao Y.-P., Zhang T.-P., Chen G. (2013). Risk factors for the occurrence of insulinoma: A case-control study. Hepatobiliary Pancreat. Dis. Int..

[126] Schenk M., Schwartz A.G., O’Neal E., Kinnard M., Greenson J.K., Fryzek J.P., Ying G.S., Garabrant D.H. (2001). Familial Risk of Pancreatic Cancer. J. Natl. Cancer Inst..

[127] Fernandez E., La Vecchia C., DeCarli A. (1996). Attributable risks for pancreatic cancer in northern Italy. Cancer Epidemiol. Biomark. Prev..

[128] Paiella S., Capurso G., Cavestro G.M., Butturini G., Pezzilli R., Salvia R., Signoretti M., Crippa S., Carrara S., Frigerio I. (2019). Results of First-Round of Surveillance in Individuals at High-Risk of Pancreatic Cancer from the AISP (Italian Association for the Study of the Pancreas) Registry. Am. J. Gastroenterol..

[129] Mocci E., Guillen-Ponce C., Earl J., Marquez M., Solera J., Salazar-López M.T., Calcedo-Arnáiz C., Vázquez-Sequeiros E., Montans J., Muñoz-Beltrán M. (2015). PanGen-Fam: Spanish registry of hereditary pancreatic cancer. Eur. J. Cancer.

[130] Zheng Z., Zheng R., He Y., Sun X., Wang N., Chen T., Chen W. (2016). Risk Factors for Pancreatic Cancer in China: A Multicenter Case-Control Study. J. Epidemiol..

[131] Schulte A., Pandeya N., Fawcett J., Fritschi L., Klein K., Risch H.A., Webb P.M., Whiteman D.C., Neale R.E. (2016). Association between family cancer history and risk of pancreatic cancer. Cancer Epidemiol..

[132] Hamada T., Yuan C., Yurgelun M.B., Perez K., Khalaf N., Morales-Oyarvide V., Babic A., Nowak J.A., Rubinson D.A., Giannakis M. (2019). Family history of cancer, Ashkenazi Jewish ancestry, and pancreatic cancer risk. Br. J. Cancer.

[133] Klein A.P., Brune K.A., Petersen G.M., Goggins M., Tersmette A.C., Offerhaus G.J.A., Griffin C., Cameron J.L., Yeo C.J., Kern S. (2004). Prospective Risk of Pancreatic Cancer in Familial Pancreatic Cancer Kindreds. Cancer Res..

[134] Piciucchi M., Capurso G., Valente R., Larghi A., Archibugi L., Signoretti M., Stigliano S., Zerboni G., Barucca V., La Torre M. (2015). Early onset pancreatic cancer: Risk factors, presentation and outcome. Pancreatol..

[135] Matsubayashi H., Takaori K., Morizane C., Maguchi H., Mizuma M., Takahashi H., Wada K., Hosoi H., Yachida S., Suzuki M. (2017). Familial pancreatic cancer: Concept, management and issues. World J. Gastroenterol..

[136] Moran A., O’Hara C., Khan S., Shack L., Woodward E., Maher E.R., Lalloo F., Evans D.G.R. (2012). Risk of cancer other than breast or ovarian in individuals with BRCA1 and BRCA2 mutations. Fam. Cancer.

[137] Mersch J., Jackson M.A., Park M., Nebgen D., Peterson S.K., Singletary C., Arun B.K., Litton J.K. (2015). Cancers associated withBRCA1andBRCA2mutations other than breast and ovarian. Cancer.

[138] Bannon S.A., Montiel M.F., Goldstein J.B., Dong W., Mork M.E., Borras E., Hasanov M., Varadhachary G.R., Maitra A., Katz M.H.G. (2018). High Prevalence of Hereditary Cancer Syndromes and Outcomes in Adults with Early-Onset Pancreatic Cancer. Cancer Prev. Res..

[139] Axilbund J.E., Argani P., Kamiyama M., Palmisano E., Raben M., Borges M., Brune K.A., Goggins M., Hruban R.H., Klein A.P. (2009). Absence of germline BRCA1 mutations in familial pancreatic cancer patients. Cancer Biol. Ther..

[140] Roberts N.J., Jiao Y., Yu J., Kopelovich L., Petersen G.M., Bondy M.L., Gallinger S., Schwartz A.G., Syngal S., Cote M.L. (2011). ATM Mutations in Patients with Hereditary Pancreatic Cancer. Cancer Discov..

[141] Kamisawa T., Wood L.D., Itoi T., Takaori K. (2016). Pancreatic cancer. Lancet.

[142] Slater E.P., Langer P., Niemczyk E., Strauch K., Butler J., Habbe N., Neoptolemos J.P., Greenhalf W., Bartsch D.K. (2010). PALB2 mutations in European familial pancreatic cancer families. Clin. Genet..

[143] Lynch H.T., Deters C.A., Snyder C.L., Lynch J.F., Villeneuve P., Silberstein J., Martin H., Narod S.A., Brand R.E. (2005). BRCA1 and pancreatic cancer: Pedigree findings and their causal relationships. Cancer Genet. Cytogenet..

[144] Vasen H.F., Gruis N.A., Frants R.R., Van Der Velden P.A., Hille E.T., Bergman W. (2000). Risk of developing pancreatic cancer in families with familial atypical multiple mole melanoma associated with a specific 19 deletion of p16 (p16-Leiden). Int. J. Cancer.

[145] Lynch H.T., Fusaro R.M., Lynch J.F., Brand R. (2007). Pancreatic cancer and the FAMMM syndrome. Fam. Cancer.

[146] Giardiello F.M., Brensinger J.D., Tersmette A.C., Goodman S.N., Petersen G.M., Booker S.V., Cruz–Correa M., Offerhaus J.A. (2000). Very high risk of cancer in familial Peutz–Jeghers syndrome. Gastroenterology.

[147] Lowenfels A.B., Maisonneuve P., DiMagno E.P., Elitsur Y., Gates L.K., Perrault J., Whitcomb D.C. (1997). International Hereditary Pancreatitis Study Group Hereditary Pancreatitis and the Risk of Pancreatic Cancer. J. Natl. Cancer Inst..

[148] Howes N., Lerch M.M., Greenhalf W., Stocken D.D., Ellis I., Simon P., Truninger K., Ammann R., Cavallini G., Charnley R.M. (2004). Clinical and genetic characteristics of hereditary pancreatitis in Europe. Clin. Gastroenterol. Hepatol..

[149] Rebours V., Boutron-Ruault M.-C., Schnee M., Férec C., Maire F., Hammel P., Ruszniewski P., Lévy P. (2008). Risk of Pancreatic Adenocarcinoma in Patients With Hereditary Pancreatitis: A National Exhaustive Series. Am. J. Gastroenterol..

[150] Whitcomb D.C., Applebaum S., Martin S.P. (1999). Hereditary Pancreatitis and Pancreatic Carcinoma. Ann. New York Acad. Sci..

[151] Aarnio M., Sankila R., Pukkala E., Salovaara R., Aaltonen L.A., De La Chapelle A., Mecklin J.-P. (1999). Cancer risk in mutation carriers of DNA-mismatch-repair genes. Int. J. Cancer.

[152] Ben Q., Xu M., Ning X., Liu J., Hong S., Huang W., Zhang H., Li Z. (2011). Diabetes mellitus and risk of pancreatic cancer: A meta-analysis of cohort studies. Eur. J. Cancer.

[153] Er K.C., Hsu C.Y., Lee Y.K., Huang M.Y., Su Y.C. (2016). Effect of glycemic control on the risk of pancreatic cancer: A nationwide cohort study. Medicine.

[154] Pang Y., Kartsonaki C., Guo Y., Bragg F., Yang L., Bian Z., Chen Y., Iona A., Millwood I.Y., Lv J. (2017). Diabetes, plasma glucose and incidence of pancreatic cancer: A prospective study of 0.5 million C hinese adults and a meta-analysis of 22 cohort studies. Int. J. Cancer.

[155] Wolpin B.M., Bao Y., Qian Z.R., Wu C., Kraft P., Ogino S., Stampfer M.J., Sato K., Ma J., Buring J.E. (2013). Hyperglycemia, insulin resistance, impaired pancreatic beta-cell function, and risk of pancreatic cancer. J. Natl. Cancer Inst..

[156] Austin M.A., Kuo E., Eeden S.K.V.D., Mandelson M.T., Brentnall T.A., Kamineni A., Potter J.D. (2013). Family History of Diabetes and Pancreatic Cancer as Risk Factors for Pancreatic Cancer: The PACIFIC Study. Cancer Epidemiol. Biomark. Prev..

[157] Perrin M.C., Terry M.B., Kleinhaus K., Deutsch L., Yanetz R., Tiram E., Calderon R., Friedlander Y., Paltiel O., Harlap S. (2007). Gestational diabetes as a risk factor for pancreatic cancer: A prospective cohort study. BMC Med..

[158] Ben Q., Cai Q., Li Z., Yuan Y., Ning X., Deng S., Wang K. (2011). The relationship between new-onset diabetes mellitus and pancreatic cancer risk: A case–control study. Eur. J. Cancer.

[159] Johnson J.A., Bowker S.L., Richardson K., Marra C.A. (2011). Time-varying incidence of cancer after the onset of type 2 diabetes: Evidence of potential detection bias. Diabetol..

[160] Setiawan V.W., Stram D.O., Porcel J., Chari S.T., Maskarinec G., Le Marchand L., Wilkens L.R., Haiman C.A., Pandol S.J., Monroe K.R. (2019). Pancreatic Cancer Following Incident Di-abetes in African Americans and Latinos: The Multiethnic Cohort. J. Natl. Cancer Inst..

[161] Wang F., Gupta S., Holly E.A. (2006). Diabetes Mellitus and Pancreatic Cancer in a Population-Based Case-Control Study in the San Francisco Bay Area, California. Cancer Epidemiol. Biomark. Prev..

[162] Li D. (2011). Diabetes and pancreatic cancer. Mol. Carcinog..

[163] Song S., Wang B., Zhang X., Hao L., Hu X., Li Z., Sun S. (2015). Long-Term Diabetes Mellitus Is Associated with an Increased Risk of Pancreatic Cancer: A Meta-Analysis. PLoS ONE.

[164] Elena J.W., Steplowski E., Yu K., Hartge P., Tobias G.S., Brotzman M.J., Chanock S.J., Stolzenberg-Solomon R.Z., Arslan A.A., Bueno-De-Mesquita H.B. (2013). Diabetes and risk of pancreatic cancer: A pooled analysis from the pancreatic cancer cohort consortium. Cancer Causes Control..

[165] Michaud D.S., Vrieling A., Jiao L., Mendelsohn J.B., Steplowski E., Lynch S.M., Wactawski-Wende J., Arslan A.A., Bueno-De-Mesquita H.B., Fuchs C.S. (2010). Alcohol intake and pancreatic cancer: A pooled analysis from the pancreatic cancer cohort consortium (PanScan). Cancer Causes Control..

[166] Janghorbani M., Dehghani M., Salehi-Marzijarani M. (2012). Systematic Review and Meta-analysis of Insulin Therapy and Risk of Cancer. Horm. Cancer.

[167] Currie C.J., Poole C.D., Gale E.A.M. (2009). The influence of glucose-lowering therapies on cancer risk in type 2 diabetes. Diabetol..

[168] Soranna D., Scotti L., Zambon A., Bosetti C., Grassi G., Catapano A., La Vecchia C., Mancia G., Corrao G. (2012). Cancer Risk Associated with Use of Metformin and Sulfonylurea in Type 2 Diabetes: A Meta-Analysis. Oncologist.

[169] Bosetti C., Rosato V., Li D., Silverman D.T., Petersen G.M., Bracci P.M., Neale R.E., Muscat J.E., Anderson K.E., Gallinger S. (2014). Diabetes, antidiabetic medications, and pancreatic cancer risk: An analysis from the International Pancreatic Cancer Case-Control Consortium. Ann. Oncol..

[170] Karp I., Sivaswamy A., Booth C. (2019). Does the use of incretin-based medications increase the risk of cancer in patients with type-2 diabetes mellitus?. Pharmacoepidemiol. Drug Saf..

[171] Buse J.B., Bethel M.A., Green J.B., Stevens S.R., Lokhnygina Y., Aschner P., Grado C.R., Tankova T., Wainstein J., Josse R. (2017). Pancreatic Safety of Sitagliptin in the TECOS Study. Diabetes Care.

[172] Lee M., Sun J., Han M., Cho Y., Lee J.-Y., Nam C.M., Kang E.S. (2019). Nationwide Trends in Pancreatitis and Pancreatic Cancer Risk Among Patients with Newly Diagnosed Type 2 Diabetes Receiving Dipeptidyl Peptidase 4 Inhibitors. Diabetes Care.

[173] Raz I., Bhatt D.L., Hirshberg B., Mosenzon O., Scirica B.M., Umez-Eronini A., Im K., Stahre C., Buskila A., Iqbal N. (2014). Incidence of Pancreatitis and Pancreatic Cancer in a Randomized Controlled Multicenter Trial (SAVOR-TIMI 53) of the Dipeptidyl Peptidase-4 Inhibitor Saxagliptin. Diabetes Care.

[174] Van Hemelrijck M., Holmberg L., Garmo H., Hammar N., Walldius G., Binda E., Lambe M., Jungner I. (2011). Association between levels of C-reactive protein and leukocytes and cancer: Three repeated measurements in the Swedish AMORIS study. Cancer Epidemiol. Biomark. Prev..

[175] Ghuman S., Van Hemelrijck M., Garmo H., Holmberg L., Malmström H., Lambe M., Hammar N., Walldius G., Jungner I., Wulaningsih W. (2017). Serum inflammatory markers and colorectal cancer risk and survival. Br. J. Cancer.

[176] Sollie S., Michaud D.S., Sarker D., Karagiannis S.N., Josephs D.H., Hammar N., Santaolalla A., Walldius G., Garmo H., Holmberg L. (2019). Chronic inflammation markers are associated with risk of pancreatic cancer in the Swedish AMORIS cohort study. BMC Cancer.

[177] Grote V.A., Kaaks R., Nieters A., Tjønneland A., Halkjær J., Overvad K., Nielsen M.R.S., Boutron-Ruault M.C., Clavel-Chapelon F., Racine A. (2012). Inflammation marker and risk of pancreatic cancer: A nested case–control study within the EPIC cohort. Br. J. Cancer.

[178] Padoan A., Plebani M., Basso D. (2019). Inflammation and Pancreatic Cancer: Focus on Metabolism, Cytokines, and Immunity. Int. J. Mol. Sci..

[179] Ekbom A., McLaughlin J.K., Karlsson B.M., Nyrén O., Gridley G., Adami H.O., Fraumeni J.F. (1994). Pancreatitis and pancreatic cancer: A popu-lation-based study. J. Natl. Cancer Inst..

[180] Goldacre M.J., Wotton C.J., Yeates D., Seagroatt V., Collier J. (2008). Liver cirrhosis, other liver diseases, pancreatitis and subsequent cancer: Record linkage study. Eur. J. Gastroenterol. Hepatol..

[181] Fernandez E., La Vecchia C., Porta M., Negri E., D’avanzo B., Boyle P. (1995). Pancreatitis and the Risk of Pancreatic Cancer. Pancreas.

[182] Lowenfels A.B., Maisonneuve P., Cavallini G., Ammann R.W., Lankisch P.G., Andersen J.R., DiMagno E.P., Andren-Sandberg A., Domellof L., International Pancreatitis Study Group (1993). Pancreatitis and the Risk of Pancreatic Cancer. N. Engl. J. Med..

[183] Malka D., Hammel P., Maire F., Rufat P., Madeira I., Pessione F., Lévy P., Ruszniewski P. (2002). Risk of pancreatic adenocarcinoma in chronic pancreatitis. Gut.

[184] Midha S., Sreenivas V., Kabra M., Chattopadhyay T.K., Joshi Y.K., Garg P.K. (2016). Genetically Determined Chronic Pancreatitis but not Alcoholic Pancreatitis Is a Strong Risk Factor for Pancreatic Cancer. Pancreas.

[185] Duell E.J., Lucenteforte E., Olson S.H., Bracci P.M., Li D., Risch H.A., Silverman D.T., Ji B.T., Gallinger S., Holly E.A. (2012). Pancreatitis and pancreatic cancer risk: A pooled analysis in the International Pancreatic Cancer Case-Control Consortium (PanC4). Ann. Oncol..

[186] Bansal P., Sonnenberg A. (1995). Pancreatitis is a risk factor for pancreatic cancer. Gastroenterology.

[187] Bracci P.M., Wang F., Hassan M.M., Gupta S., Li D., Holly E.A. (2009). Pancreatitis and pancreatic cancer in two large pooled case–control studies. Cancer Causes Control..

[188] Karlson B.M., Ekbom A., Josefsson S., McLaughlin J.K., Fraumeni J.F., Nyren O. (1997). The risk of pancreatic cancer following pan-creatitis: An association due to confounding?. Gastroenterology.

[189] Talamini G., Falconi M., Bassi C., Sartori N., Salvia R., Caldiron E., Frulloni L., Di Francesco V., Vaona B., Bovo P. (1999). Incidence of cancer in the course of chronic pancreatitis. Am. J. Gastroenterol..

[190] Ueda J., Tanaka M., Ohtsuka T., Tokunaga S., Shimosegawa T. (2013). Surgery for chronic pancreatitis decreases the risk for pancreatic cancer: A multicenter retrospective analysis. Surgery.

[191] Raimondi S., Lowenfels A.B., Morselli-Labate A.M., Maisonneuve P., Pezzilli R. (2010). Pancreatic cancer in chronic pancreatitis; aeti-ology, incidence, and early detection. Best Pract. Res. Clin. Gastroenterol..

[192] Pak L.M., Schattner M.A., Balachandran V., D’Angelica M.I., DeMatteo R.P., Kingham T.P., Jarnagin W.R., Allen P.J. (2018). The clinical utility of immuno-globulin G4 in the evaluation of autoimmune pancreatitis and pancreatic adenocarcinoma. HPB.

[193] Raina A., Krasinskas A.M., Greer J.B., Lamb J., Fink M.E., Moser A.J., Iii H.J.Z., Slivka A., Whitcomb D.C. (2008). Serum Immunoglobulin G Fraction 4 Levels in Pancreatic Cancer: Elevations Not Associated With Autoimmune Pancreatitis. Arch. Pathol. Lab. Med..

[194] Ngwa T., Law R., Hart P., Smyrk T.C., Chari S.T. (2015). Serum IgG4 elevation in pancreatic cancer: Diagnostic and prognostic signifi-cance and association with autoimmune pancreatitis. Pancreas.

[195] Chang M.-C., Liang P.-C., Jan S., Yang C.-Y., Tien Y.-W., Wei S.-C., Wong J.-M., Chang Y.-T. (2014). Increase diagnostic accuracy in differentiating focal type autoimmune pancreatitis from pancreatic cancer with combined serum IgG4 and CA19-9 levels. Pancreatol..

[196] Talar-Wojnarowska R., Gasiorowska A., Olakowski M., Dranka-Bojarowska D., Lampe P., Śmigielski J., Kujawiak M., Grzegorczyk J., Małecka-Panas E. (2014). Utility of serum IgG, IgG4 and carbonic anhydrase II antibodies in distinguishing autoimmune pancreatitis from pancreatic cancer and chronic pancreatitis. Adv. Med Sci..

[197] Sollie S., Santaolalla A., Michaud D.S., Sarker D., Karagiannis S.N., Josephs D.H., Hammar N., Walldius G., Garmo H., Holmberg L. (2020). Serum Immunoglobulin G Is Associated with Decreased Risk of Pancreatic Cancer in the Swedish AMORIS Study. Front. Oncol..

[198] Turner M.C. (2011). Epidemiology: Allergy history, IgE, and cancer. Cancer Immunol. Immunother..

[199] Anderson L.N., Cotterchio M., Gallinger S. (2009). Lifestyle, dietary, and medical history factors associated with pancreatic cancer risk in Ontario, Canada. Cancer Causes Control..

[200] Cotterchio M., Lowcock E., Hudson T.J., Greenwood C., Gallinger S. (2014). Association between Allergies and Risk of Pancreatic Cancer. Cancer Epidemiol. Biomark. Prev..

[201] Gandini S., Lowenfels A.B., Jaffee E.M., Armstrong T.D., Maisonneuve P. (2005). Allergies and the risk of pancreatic cancer: A me-ta-analysis with review of epidemiology and biological mechanisms. Cancer Epidemiol. Biomark. Prev..

[202] Gomez-Rubio P., Zock J.-P., Rava M., Marquez M., Sharp L., Hidalgo M., Carrato A., Ilzarbe L., Michalski C., Molero X. (2015). Reduced risk of pancreatic cancer associated with asthma and nasal allergies. Gut.

[203] Eppel A., Cotterchio M., Gallinger S. (2007). Allergies are associated with reduced pancreas cancer risk: A population-based case–control study in Ontario, Canada. Int. J. Cancer.

[204] Olson S.H. (2011). Selected medical conditions and risk of pancreatic cancer. Mol. Carcinog..

[205] Kreiger N., Lacroix J., Sloan M. (2001). Hormonal Factors and Pancreatic Cancer in Women. Ann. Epidemiol..

[206] Lucenteforte E., Zucchetto A., Bosetti C., Talamini R., Negri E., Serraino D., Franceschi S., Lipworth L., La Vecchia C. (2011). Reproductive and hormonal factors and pan-creatic cancer risk in women. Pancreas.

[207] Skinner H.G., Michaud D.S., Colditz G.A., Giovannucci E.L., Stampfer M.J., Willett W.C., Fuchs C.S. (2003). Parity, reproductive factors, and the risk of pancreatic cancer in women. Cancer Epidemiol. Biomark. Prev..

[208] Zhang Y., Coogan P.F., Palmer J.R., Strom B.L., Rosenberg L. (2010). A case-control study of reproductive factors, female hormone use, and risk of pancreatic cancer. Cancer Causes Control..

[209] Karlson B.M., Wuu J., Hsieh C.C., Lambe M., Ekbom A. (1998). Parity and the risk of pancreatic cancer: A nested case-control study. Int. J. Cancer.

[210] Andersson G., Borgquist S., Jirström K. (2018). Hormonal factors and pancreatic cancer risk in women: The Malmö Diet and Cancer Study. Int. J. Cancer.

[211] Bueno de Mesquita H.B., Maisonneuve P., Moerman C.J., Walker A.M. (1992). Anthropometric and reproductive variables and exocrine carcinoma of the pancreas: A population-based case-control study in The Netherlands. Int. J. Cancer.

[212] Fernández E., La Vecchia C., D’Avanzo B., Negri E. (1995). Menstrual and reproductive factors and pancreatic cancer risk in women. Int. J. Cancer.

[213] Duell E.J., Holly E.A. (2005). Reproductive and menstrual risk factors for pancreatic cancer: A population-based study of San Francisco Bay Area women. Am. J. Epidemiol..

[214] Berrington de Gonzalez A., Sweetland S., Spencer E. (2003). A meta-analysis of obesity and the risk of pancreatic cancer. Br. J. Cancer.

[215] Stolzenberg-Solomon R.Z., Schairer C., Moore S., Hollenbeck A., Silverman D.T. (2013). Lifetime adiposity and risk of pancreatic cancer in the NIH-AARP Diet and Health Study cohort. Am. J. Clin. Nutr..

[216] Larsson S.C., Orsini N., Wolk A. (2007). Body mass index and pancreatic cancer risk: A meta-analysis of prospective studies. Int. J. Cancer.

[217] Jiao L., De Gonzalez A.B., Hartge P., Pfeiffer R.M., Park Y., Freedman D.M., Gail M.H., Alavanja M.C.R., Albanes D., Freeman L.E.B. (2010). Body mass index, effect modifiers, and risk of pancreatic cancer: A pooled study of seven prospective cohorts. Cancer Causes Control..

[218] Noor N.M., Banim P.J., Luben R.N., Khaw K.T., Hart A.R. (2016). Investigating Physical Activity in the Etiology of Pancreatic Cancer: The Age at Which This Is Measured Is Important and Is Independent of Body Mass Index. Pancreas.

[219] Kabat G.C., Kim M.Y., Chlebowski R.T., Vitolins M.Z., Wassertheil-Smoller S., Rohan T.E. (2017). Serum lipids and risk of obesity-related cancers in postmenopausal women. Cancer Causes Control..

[220] La Torre G., Sferrazza A., Gualano M.R., De Waure C., Clemente G., De Rose A.M., Nicolotti N., Nuzzo G., Siliquini R., Boccia A. (2014). Investigating the synergistic interaction of diabetes, tobacco smoking, alcohol consumption, and hypercholesterolemia on the risk of pancreatic cancer: A case-control study in Italy. Biomed. Res. Int..

[221] Rosato V., Tavani A., Bosetti C., Pelucchi C., Talamini R., Polesel J., Serraino D., Negri E., La Vecchia C. (2011). Metabolic syndrome and pancreatic cancer risk: A case-control study in Italy and meta-analysis. Metabolism.

[222] Zaleska M., Mozenska O., Bil J. (2018). Statins use and cancer: An update. Futur. Oncol..

[223] Bradley M.C., Hughes C.M., Cantwell M.M., Murray L.J. (2010). Statins and pancreatic cancer risk: A nested case–control study. Cancer Causes Control..

[224] Carey F.J., Little M.W., Pugh T.F.G., Ndokera R., Ing H., Clark A., Dennison A., Metcalfe M.S., Robinson R.J., Hart A.R. (2013). The Differential Effects of Statins on the Risk of Developing Pancreatic Cancer: A Case–Control Study in Two Centres in the United Kingdom. Dig. Dis. Sci..

[225] Chiu H.F., Chang C.C., Ho S.C., Wu T.N., Yang C.Y. (2011). Statin use and the risk of pancreatic cancer: A population-based case-control study. Pancreas.

[226] Cui X., Xie Y., Chen M., Li J., Liao X., Shen J., Shi M., Li W., Zheng H., Jiang B. (2012). Statin use and risk of pancreatic cancer: A meta-analysis. Cancer Causes Control..

[227] Kirkegård J., Lund J.L., Mortensen F.V., Cronin-Fenton D. (2020). Statins and pancreatic cancer risk in patients with chronic pancreatitis: A Danish nationwide population-based cohort study. Int. J. Cancer.

[228] Esposito K., Giugliano D. (2004). The metabolic syndrome and inflammation: Association or causation?. Nutr. Metab. Cardiovasc. Dis..

[229] Inoue M., Noda M., Kurahashi N., Iwasaki M., Sasazuki S., Iso H., Tsugane S. (2009). Impact of metabolic factors on subsequent cancer risk: Results from a large-scale population-based cohort study in Japan. Eur. J. Cancer Prev..

[230] Risch H.A., Yu H., Lu L., Kidd M.S. (2010). ABO Blood Group, Helicobacter pylori Seropositivity, and Risk of Pancreatic Cancer: A Case-Control Study. J. Natl. Cancer Inst..

[231] Risch H.A., Lu L., Wang J., Zhang W., Ni Q., Gao Y.-T., Yu H. (2013). ABO Blood Group and Risk of Pancreatic Cancer: A Study in Shanghai and Meta-Analysis. Am. J. Epidemiol..

[232] Wolpin B.M., Kraft P., Gross M., Helzlsouer K., Bueno-De-Mesquita H.B., Steplowski E., Stolzenberg-Solomon R.Z., Arslan A.A., Jacobs E.J., Lacroix A. (2010). Pancreatic Cancer Risk and ABO Blood Group Alleles: Results from the Pancreatic Cancer Cohort Consortium. Cancer Res..

[233] Tada M., Kawabe T., Arizumi M., Togawa O., Matsubara S., Yamamoto N., Nakai Y., Sasahira N., Hirano K., Tsujino T. (2006). Pancreatic Cancer in Patients With Pancreatic Cystic Lesions: A Prospective Study in 197 Patients. Clin. Gastroenterol. Hepatol..

[234] Matsubara S., Tada M., Akahane M., Yagioka H., Kogure H., Sasaki T., Arizumi T., Togawa O., Nakai Y., Sasahira N. (2012). Incidental Pancreatic Cysts Found by Magnetic Resonance Imaging and Their Relationship with Pancreatic Cancer. Pancreas.

[235] Bray F., Ferlay J., Soerjomataram I., Siegel R.L., Torre L.A., Jemal A. (2018). Global cancer statistics 2018: GLOBOCAN estimates of incidence and mortality worldwide for 36 cancers in 185 countries. Ca Cancer J. Clin..

[236] Zambirinis C.P., Pushalkar S., Saxena D., Miller G. (2014). Pancreatic Cancer, Inflammation, and Microbiome. Cancer J..

[237] Singh G.K., Jemal A. (2017). Socioeconomic and Racial/Ethnic Disparities in Cancer Mortality, Incidence, and Survival in the United States, 1950–2014: Over Six Decades of Changing Patterns and Widening Inequalities. J. Environ. Public Health.

[238] Lu P.-Y., Shu L., Shen S.-S., Chen X.-J., Zhang X.-Y. (2017). Dietary Patterns and Pancreatic Cancer Risk: A Meta-Analysis. Nutrients.

