# Supplementary material for: Pancreatic Cancer Exposome Profile to Aid Early Detection and Inform Prevention Strategies
Source: J Clin Med. 2021 Apr 13;10(8):1665. doi: 10.3390/jcm10081665 (PMC8069449; doi:10.3390/jcm10081665)
Supplement: Supplementary file 1 [file jcm-10-01665-s001.pdf]

Supplementary Materials

Supplementary table 1 contains ecological, observational and interventional studies addressing specific external exposures in PCa.

Author, Year	Country	Study Design	Population	Exposure	Findings	Observations
Korc, 2017 (26)	USA	Meta-analysis	10 studies	Smoking	(+) PCa[1] risk: current vs. never smokers (RR[2] 1.66; 95%CI[3] 1.38-1.98); former vs. never smokers (RR 1.40; 95%CI 1.16-1.67); current vs. former smokers (RR 1.18; 95%CI 1.00-1.38)	
Kuzmickiene, 2012 (31)	Lithuania	Cohort	PCa cases (n=77)	Smoking	(+) PCa risk: higher smoking intensity >20 cigarettes/day (HR[4] 2.60; 95%CI 1.42-3.09); >30 pack-years (HR 2.24; 95%CI 1.12-4.49); age < 18 years (HR 2.29; 95%CI 1.11-4.70)	Adjusted for age, BMI[5], education, and alcohol consumption
Wang, 2014 (32)	China	Case-control	Cases (n=307), controls (n=1,228)	Smoking	(+) PCa risk: current smokers (OR[6] 1.71; 95%CI 1.25-2.35); >20 cigarettes/day (OR 2.18; 95%CI 1.66-3.69)	
Heinen, 2010 (33)	The Netherlands	Cohort	PCa cases (n=520)	Smoking	(+) PCa risk: former smokers (HR 1.34; 95%CI 1.02-1.75), current smokers (HR 1.82; 95%CI 1.40-2.38); per increment of 10 years (HR 1.15; 95%CI 1.08-1.22); per 10 cigarettes per day (HR 1.08; 95%CI 0.98-1.19).	
Hassan, 2007 (25)	USA	Case-control	Cases (n=808), controls (n=808)	Smoking, Passive Smoking and Non-Cigarette Smoking	(+) PCa risk: Men: smoking and DM[7] (OR 3.6; 95%CI 1.8-7.2); smoking and FHPC[9] (OR 2.4; 95%CI 1.1-5.4). Women: smoking and DM (OR 9.3; 95%CI 2.0-44.1); smoking and FHPC (OR 10.1; 95%CI 2.2-45.9); (+) PCa risk: current smoking (OR 1.7; 95%CI 1.4-2.2); passive smoking (OR 1.7; 95%CI 1.03-2.6); cigar smoking (OR 2.2; 95%CI 1.0-4.7)	Adjusted for age, sex, ethnicity, history of DM, FH[8] of cancer, alcohol consumption, education level, and state of residency.
Antwi, 2016 (24)	USA	Case-control	Cases (n=817), controls (n=1,756)	Smoking	(+) PCa risk: former vs. never smokers (OR 1.29; 95%CI 1.07-1.54); current vs. never smokers (OR 3.40; 95%CI 2.28-5.07); high DII[10] score and current smokers (OR 4.79; 95%CI 3.00-7.65)	
Matsubayashi, 2011 (27)	Japan	Case-control	Cases (n=577), controls (n=577)	Smoking	(+) PCa risk: FH of Pca (OR 2.5; 95%CI 1.37-4.38); current smokers (OR 1.5; 95%CI 1.12-1.89); DM (OR 1.7; 95%CI 1.23-2.28); 3 factors combined (OR 10.1; 95%CI 1.25-81.32)	

Villeneuve, 2004 (41)	Canada	Case-control	Cases (n=583), controls (n=4,813)	Environmental Tobacco Smoke (ETS)	(+/-) PCa risk: ETS[11] exposure (OR 1.21; 95%CI 0.60-2.44)	Adjusted for age, sex, BMI, SES[12] and providence of residency
Lo, 2007 (38)	Egypt	Case-control	Cases (n=194), controls (n=194)	Passive Smoking	(+) PCa risk: passive smoking (OR 6.0; 95%CI 2.4-14.8); cigarette smoking (OR 4.5; 95%CI 1.9-10.7); cigarette and non-cigarette smokers (OR 7.8; 95%CI 3.0-20.6).	Adjusted for age, sex and area of residence
Korc, 2017 (26)	USA	Meta-Analysis	10 studies	Alcohol Consumption	(+) PCa risk: Heavy vs. never drinkers (RR 1.29; 95%CI 1.20-1.38); heavy vs. light drinkers (RR 1.36; 95%CI 1.02-1.80)	
Lu, 2006 (46)	China	Case-control	Cases (n=119), controls (n=238)	Alcohol Consumption	(+) PCa risk: Heavy drinkers (>20 cup-years) (OR 3.68; 95%CI 1.60-8.44)	
Genkinger, 2009 (43)	USA	Meta-Analysis	14 studies	Alcohol Consumption	(+) PCa risk: heavy drinkers (>30g/day) (RR 1.22; 95%CI 1.03-1.45); alcohol consumption in females (RR 1.41; 95%CI 1.07-1.85)	Adjusted forsmoking, history of DM, energy intake and age.
Klein, 2013 (45)	USA	Pooled Analysis	Cases (n=3,349), controls (n=3,654)	Alcohol Consumption	(+) PCa risk: heavy drinkers (>3 drinks/day) (OR 1.45; 95%CI 1.19-1.76)	
Brand, 2009 (34)	USA	Pooled Analysis	2 cohorts (CIRF and UMPCR)	Alcohol Consumption	(+) PCa risk: current vs. never drinkers; current drinkers were diagnosed at significantly younger age than never drinkers (65yrs vs.70 - CIRF; 60yrs vs. 65yrs- UMPCR)	
Hassan, 2007 (37)	USA	Case-Control	Cases (n=808), controls (n=808)	Alcohol Consumption	(+) PCa risk: heavy drinkers (>60 mL/day) (OR 1.6; 95%CI 1.1-2.5)	Adjusted for age, sex, ethnicity, smoking, history of DM, FH of PCa, history of pancreatitis, education level, and state of residency
La Torre, 2014 (220)	Italy	Case-Control	Cases (n=80), controls (n=392)	Alcohol Consumption	(+) PCa risk: alcohol consumption (OR 2.25; 95%CI 1.30-3.89); alcohol consumption and hypercholesterolemia (OR 5.11; 95%CI 2.66-9.82); alcohol consumption and DM (OR 3.31; 95%CI 1.50-7.32)	Adjusted for work activity, DM and ever smoking
Naudin, 2018 (48)	EPIC Study	Cohort	PCa cases (n=1,283)	Alcohol Consumption	(+) PCa risk: heavy drinkers (>60g/day)(HR 1.77; 95%CI 1.06-2.95); beer consumption (>40g/day)(HR 1.58; 95%CI 1.97-2.34); spirits consumption (>10g/day)(HR 1.41; 95%CI 1.93-2.94)	Only in men
Gupta, 2010 (44)	USA	Case-Control	Cases (n=532), controls (n=1,701)	Alcohol Consumption	(+) PCa risk: heavy drinkers (22-35 drinks/week)(OR 2.2; 95%CI 1.1-4.0); (>35 drinks/week)(OR 2.6; 95%CI 1.6-7.5)	Adjusted for age, energy intake, BMI, ethnicity, smoking, DM, and physical activity

McWilliams, 2016 (47)	USA	Meta-Analysis	8 Studies	Alcohol Consumption	(+) PCa risk: heavy drinkers (>26 g/day) EOPC[13] (OR 1.49; 95%CI 1.21-1.84); VEOPC[14] (OR 2.18; 95%CI 1.17-4.09)	Adjusted for FH of PCa, history of DM, sex, smoking, BMI, and study site
Lu, 2017 (238)	China	Meta-Analysis	32 Studies	Dietary Patterns	(-) Pca risk: highest category of healthy pattern (OR 0.86; 95%CI 0.77-0.95); (+) PCa risk: highest category of western-type diet (OR 1.24; 95%CI 1.06-1.45)	Healthy pattern: high intake of vegetables, fruits, whole grains, olive oil, fish, soy, poultry and low-fat dairy products. Western-type pattern: red and/or processed meat, refined grains, sweets, high-fat gravy and low intake of fruits and vegetables.
Antwi, 2018 (54)	USA	Meta-Analysis	11 Studies (PanScan and PanC4)	Dietary Patterns	(+) PCa risk: High DII score -Q5 vs. Q1 (OR 2.20; 95%CI 1.85-2.61)	
Antwi, 2016 (24)	USA	Case-Control	Cases (n=817), controls (n=1,756)	Dietary Patterns	(+) PCa risk: High DII score (OR 2.54; 95%CI 1.87-3.46); high DII score and DM (OR 6.03; 95%CI 3.00-7.65)); high DII score and smoking (OR 4.79; 95%CI 3.00-7.65)	
Shivappa, 2015 (56)	Italy	Case-Control	Cases (n=326), controls (n=652)	Dietary Patterns	(+) PCa risk: High DII score Q[15]5vs. Q1 (OR 2.48; 95%CI 1.50-4.10)	
Larsson, 2012 (55)	Sweden	Meta-Analysis	11 Studies	Dietary Patterns	(+) PCa risk: 120g increase per day in red meat (RR 1.13; 95%CI 0.93-1.39); 50g per day increase in processed meat consumption (RR 1.19; 1.04-1.36)	
Ghadirian, 1995 (239)	Canada	Case-Control	Cases (n=179), Controls (n=239)	Dietary Patterns	(+) Pca risk: high salt consumption (RR 4.28; 95%CI 2.20-8.36); smoked meat (RR 4.68; 95%CI 2.05-10.69); dehydrated food (RR 3.10; 95%CI 1.55-6.22); fried food (RR 3.84; 95%CI 1.74-8.48); (-) Pca risk: food with no preservatives (RR 0.08; 95%CI 0.01-0.59); raw food (RR 0.28; 95%CI 0.10-0.75)	
Abdelrehim, 2018 (240)	Egypt	Case-Control	Cases (n=75), controls (n=149)	Dietary Patterns	(+) PCa risk: High DII (OR 9.88; 95%CI 2.56-38.1); vegetable intake (OR 0.24; 95%CI 0.07-0.85)	Adjusted forage, smoking, physical activity, mental stress and total energy intake
Zheng, 2018 (67)	USA	Cohort	PCa cases (n=328)	Dietary Patterns	No significant associations	
Zheng, 2019 (68)	USA	Cohort	PCa cases (n=2,824)	Dietary Patterns	No significant associations	
Mueller, 2010 (70)	Singapore	Cohort	PCa cases (n=140)	Soft drink and juice consumption	(+) PCa risk: >2 drinks/week (HR 1.87; 95%CI 1.10-3.25)	Adjusted for age, sex, ethnicity, year of interview, education, smoking,

						PA[16], alcohol, added sugar and candy
Schernhammer, 2005 (86)	USA	Cohort	PCa cases (n=379)	Soft drink and juice consumption	(+) PCa risk: >3 drinks/week (RR 1.57; 95%CI 1.02-2.41)	
Nothlings, 2007 (72)	USA	Cohort	PCa cases (n=434)	Soft drink and juice consumption	(+) PCa risk: Q4 vs. Q1- fructose (RR 1.35; 95%CI 1.02-1.80) and other sugars; juice and fruit intake (RR 1.37; 95%CI 1.02-1.84)	Adjusted for ethnicity, age at cohort entry, smoking status, pack-years of smoking, family history of pancreatic cancer, energy intake, intakes of red meat and processed meat, and BMI
Guertin, 2015 (9)	USA	Cohort	PCa cases (n=1,541)	Coffee consumption	No significant associations	
Bidel, 2013 (241)	Finland	Cohort	PCa cases (n=235)	Coffee consumption	No significant associations	
Turati, 2011 (74)	Italy	Pooled Analysis	Cases (n=688), controls (n=2,204)	Coffee and tea consumption	(+) Pca risk: coffee drinkers (OR 1.34; 95%CI 1.01-1.77) - no trend in risk associated to dose and duration / No associations for decaffeinated coffee or tea.	Adjusted for age, sex, center, year of interview, education, BMI, smoking, alcohol drinking, and DM
Banim, 2013 (75)	UK	Cohort	PCa cases (n=49)	Dietary Antioxidants	(-) PCa risk: Q4 vs. Q1 – vitamin C (HR 0.33; 95%CI 0.13-0.84); Selenium (HR 0.49; 95%CI 0.26-0.93), Vitamin E (HR 0.57; 95%CI 0.29-1.09)	
Lucas, 2016 (76)	Italy	Case-Control	Cases (n=326), controls (n=652)	Dietary Antioxidants	(-) Pca risk: Q3 vs. Q1 antioxidants (OR 0.61; 95%CI 0.39-0.94)	
Lin, 2005 (58)	Japan	Case-Control	Cases (n=109), Controls (n=109)	Dietary Antioxidants	(-) PCa risk: Q3 vs. Q1- Vitamin C (OR0.45; 95%CI 0.22-0.94) / No associations found with Vitamin E	Adjusted for energy intake
Waterhouse, 2015 (77)	USA	Meta-Analysis	9 Studies	Vitamin D	(+) Pca risk: Dietary vitamin D - per 110 IU/day (OR 1.13; 95%CI 1.07-1.19); >230 vs <110 UI/day (OR 1.31; 95%CI 1.10-1.55)	
Stolzenberg-Solomon, 2009 (79)	Finland	Case-Control	Cases (n=184), Controls (n=368)	Vitamin D	No significant associations with serum Vitamin D / (+) PCa risk in patients with low estimated annual residential solar UBV exposure	
Stolzenberg-Solomon, 2010 (78)	USA	Meta-Analysis	8 Sudies	Vitamin D	(+) PCa risk: High serum Vitamin D (>100 nmol/L)(OR 2.12; 95%CI 1.23-3.64)	
Chuang, 2011 (80)	European Prospective Investigation	Case-Control	Cases (n=463), Controls (n=463)	Folate Intake	No significant associations	

	into Cancer and Nutrition (EPIC) study					
Stolzenberg-Solomon, 1999 (82)	Finland	Case-Control	Cases (n=126), Controls (n=247)	Folate Intake	(-) PCa risk: Q3 vs. Q1 – Folate (OR 0.45; 95%CI 0.24-0.82)	
Skinner, 2004 (207)	USA	Cohort	PCa cases (n=326)	Folate Intake	No significant associations	Adjusted for age, energy intake, smoking, BMI, DM, height.
Gong, 2009 (81)	USA	Case-Control	Cases (n=532), Controls (n=1,701)	Folate Intake	(-) Pca risk: Q5 vs Q1 - Folate intake (OR 0.67; 95%CI 0.48-0.93)	Adjusted for age, sex, total energy intake, BMI, DM, and alcohol consumption
Bradley, 2010 (223)	UK	Case-Control	Cases (n=1,141), Controls (n=7,954)	Nonsteroidal Anti-inflammatory Drugs	(-) PCa risk: Chronic NSAID[17] use (OR 0.78; 95%CI 0.62-0.97)	
Archibugi, 2017 (83)	Italy	Case-Control	Cases (n=408), Controls (n=816)	Nonsteroidal Anti-inflammatory Drugs	No significant associations	
Khalaf, 2018 (87)	USA	Pooled Analysis	PCa cases (n=1,112)	Nonsteroidal Anti-inflammatory Drugs	(-) Pca risk: regular use of aspirin in patients with DM (RR 0.71; 95%CI 0.54-0.94) / No other significant associations	
Schernhammer, 2004 (86)	USA	Cohort	PCa cases (n=161)	Nonsteroidal Anti-inflammatory Drugs	(+) PCa risk: chronic NSAID use (RR 1.58; 95%CI 1.03-2.43) / No associations related with dose	Adjusted for age, DM, smoking, physical activity and BMI– Only women in this analysis
Coogam, 2000 (85)	USA	Cohort	PCa cases (n=504)	Nonsteroidal Anti-inflammatory Drugs	No significant associations	
Wang, 2013 (89)	China	Meta-Analysis	10 Studies	Hepatitis B Virus	(+)PCa risk: Chronic or inactive HBV[18] infection (OR 1.60; 95%CI 1.26-2.06)	
Xing, 2013 (88)	China	Meta-Analysis	10 Studies	Hepatitis B Virus and Hepatitis C Virus	(+) PCa risk: HBV infection (OR 1.28; 1.11-1.48); HCV[19] infection (OR 1.21; 95%CI 1.02-1.44)	*One study included in meta-analysis above
Song, 2019 (90)	China	Cohort	PCa cases (n=504)	Hepatitis B Virus	(+) PCa risk: HBV infection (HR 1.65; 95%CI 1.03-2.65)	
Chang, 2014 (91)	China	Case-Control	Cases (n=585), Control (n=1,716)	Hepatitis B Virus and Hepatitis C Virus	No significant associations	

Tang, 2014 (92)	USA	Cohort	PCa cases (n=200)	Hepatitis B Virus	No significant associations	
Ai, 2015 (94)	China	Cohort	PCa cases (n=56)	Helicobacter Pylori	H. Pylori infection rate was 64% in the observation group vs. 47% in the control group	
Hirabayashi, 2019 (96)	Japan	Cohort	PCa cases (n=119)	Helicobacter Pylori	No significant associations	
Risch, 2014 (231)	China	Case-Control	Cases (n=761), Controls (n=794)	Helicobacter Pylori	No significant associations	
Risch, 2013 (231)	China	Meta-Analysis	24 Studies	Helicobacter Pylori	(+) PCa risk: H. Pylori infection – ABO group A, B and AB (OR 1.40 (95%CI 1.32-1.49); OR 1.38 (95%CI 1.16-1.64); OR 1.52 (95%CI 1.24-1.84), respectively)	
Lindkvist, 2008 (97)	Sweden	Cohort	PCa cases (n=87)	Helicobacter Pylori	No significant associations*	
De Martel, 2008 (95)	USA	Case-Control	Cases (n=104), Controls (n=597)	Helicobacter Pylori	No significant associations	
Chen, 2016 (98)	China	Cohort	PCa cases (n=)	Helicobacter Pylori	No significant associations	
Caygill, 1994 (103)	UK	Cohort	PCa cases (n=)	Typhoid and Paratyphoid Carriers	(+) Pca risk: chronic typhoid and paratyphoid carriers (OR 8.1)	
Gotland, 2020 (104)	Denmark	Cohort	PCa cases (n=)	Staphylococcus Aureus Bacteremia	(+) PCa risk: Staphylococcus aureus bacteremia (IRR 2.8; 95%CI 1.27-6.16)	
Fan, 2018 (101)	USA	Case-Control	Cases (n=121), Controls (n=371)	Oral Bacteria	(+) Pca risk: Porphyromonas gingivalis (OR 1.60; 95%CI 1.15-2.22) and Aggregatibacter actinomycetemcomitans (OR 2.20; 95%CI 1.16-4.18) / (-) Pca risk: Non-oral bacteria- Phylum Fusobacteria (OR 0.94; 95%CI 0.89-0.99) and its genus Leptotrichia (OR 0.87; 95%CI 0.79-0.95)	Adjusted for age, ethnicity, sex, BMI, smoking, alcohol intake, and DM
Michaud, 2013 (102)	European Prospective Investigation into Cancer and Nutrition (EPIC) study	Cohort	PCa cases (n=405)	Oral Bacteria	(+) Pca risk: porphyromonas gingivalis (OR 2.14; 95%CI 1.05-4.36) / (-) Pca risk: antibodies against non-pathogenic oral bacteria (OR 0.55; 95%CI 0.36-0.83)	
Michaud, 2007 (100)	USA	Cohort	PCa cases (n=216)	Periodontal Disease	(+) PCa risk: periodontal disease (RR 1.64; 95% CI 1.19-2.26)	Adjusted for age, smoking, profession, ethnicity, geographic location, physical activity, BMI,

						height, cholecystectomy, NSAIDs, and baseline teeth number
Gerlovin, 2019 (99)	USA	Cohort	PCa cases (n=78)	Periodontal Disease	No significant associations	
Bosetti, 2013 (109)	Italy	Meta-Analysis	10 Studies	Peptic Ulcer Disease	No significant associations	Adjusted for study, study center (for multicenter studies), age, sex, race/ethnicity, education, body mass index, tobacco smoking, alcohol drinking, history of diabetes, and history of pancreatitis.
Bao, 2010 (105)	USA	Cohort	PCa cases (n=274)	Peptic Ulcer Disease	(+) Pca risk: gastric ulcer (RR 1.83; 95%CI 1.13-2.97)	Adjusted for age, smoking, DM, BMI, PA
Luo, 2007 (106)	Sweden	Cohort	PCa cases (n=)	Peptic Ulcer Disease	(+) Pca risk: gastric ulcer (SIR[21] 1.5; 95%CI 1.1-2.0); duodenal ulcer (SIR 1.6; 95%CI 1.1-2.1)	
Ko, 2007 (242)	USA	Case-Control	Cases (n=532), Controls(n=1,701)	Peptic Ulcer Disease	(+) PCa rsk: duodenal ulcer (OR 2.3; 95%CI 1.1-4.6)	
Lo, 2007 (38)	Egypt	Case-Control	Cases (n=194), Controls (n=194)	Occupational Exposures	(+) PCa risk: Pesticide exposure (OR 2.6; 95%CI 0.97-7.2)	Adjusted for age, sex and residence
Kauppinen, 1995 (120)	Finland	Case-Control	Cases (n=595), Controls (n=1,622)	Occupational Exposures	(+) PCa risk: Ionizing radiation (OR 4.3; 95%CI 1.6-11.4); inorganic dust containing silica (OR 2.0; 95%CI 1.2-3.5)	
Hoppin, 2000 (114)	USA	Case-Control	Cases (n=108), Controls (n=82)	Occupational Exposures	(+) PCa risk: Pesticide exposure (Q3 vs Q1)(OR 4.2; 95%CI 1.8-9.4)	
Antwi, 2015 (118)	USA	Case-Control	Cases (n=2,092), Controls (n=2,353)	Occupational Exposures	(+) Pca risk: pesticides (OR 1.21; 95%CI 1.02-1.44); asbestos (OR 1.54; 95%CI 1.23-1.92); benzene (OR 1.70; 95%CI 1.23-2.35); chlorinated hydrocarbons (OR 1.63; 95%CI 1.32-2.02)	Self-reported and adjusted for age, sex, smoking, history of DM, BMI, and education.
Kachuri, 2017 (119)	Canada	Cohort	PCa cases (n=500)	Occupational Exposures	(+) PCa risk: Pesticide Exposure- women (HR 1.36; 95%CI 1.07-1.72)	Adjusted for age, province of residence, and education level
Andreotti, 2009 (111)	USA	Cohort	PCa cases (n=93)	Occupational Exposures	(+) PCa risk: Pesticide Exposure (OR 3.0; 95%CI 1.3-7.2)	
Gandini, 2005 (201)	Italy	Meta-Analysis	14 Studies	Allergies	(-) PCa risk: Allergies (RR 0.82; 95%CI 0.68-0.99); Atopy (RR 0.71; 95%CI 0.64-0.80)	
Cotterchio, 2014 (200)	Italy	Case-Control	Cases (n=345), Controls (n=1,285)	Allergies	(-) PCa risk: Hay fever (OR 0.68; 95%CI 0.52-0.89); Dust/Mold Allergy (OR 0.49; 95%CI 0.31-0.78); Animal Allergy (OR 0.68; 95%CI 0.46-0.99)	Adjusted for age
Dai, 1995 (243)	China	Case-Control	Cases (n=108), Controls (n=275)	Allergies	(-) PCa risk: Allergies (0.60; 95%CI 0.40-1.0)	Adjusted for age, sex, smoking and income

Gomez-Rubio, 2015 (202)	Spain	Case-Control / Meta-Analysis	Cases (n=1,297), Controls (n=1,024) / 10 Studies	Allergies	(-) PCa risk: Asthma (OR 0.64 (95% 0.47-0.88); metaOR 0.73 (95% 0.59-0.89); long-standing asthma (>17yrs; OR 0.39 (95%CI 0.24-0.65)) / Nasal allergy (OR 0.66 (95%CI 0.52-0.83); metaOR 0.60 (95%CI 0.50-0.72)	
Anderson, 2009 (199)	Canada	Case-Control	Cases (n=422), Controls (n=312)	Allergies	(-) PCa risk: Allergies (OR 0.40; 95%CI 0.26-0.59)	Adjusted for age
Eppel, 2007 (203)	Canada	Case-Control	Cases (n=276), Controls (n=378)	Allergies	(-) PCa risk: Allergies (OR 0.43; 95%CI 0.29-0.63)	Adjusted for age

PCa, pancreatic cancer; [2]RR, relative risk; [3]CI, confidence interval; [4]HR, hazard ratio; [5]BMI, body mass index; [6]OR, odds ratio; [7]DM, diabetes mellitus; [8]FH, family history; [9]FHPC, family history of pancreatic cancer; [10]DII, dietary inflammatory index; [11]ETS, environmental tobacco exposure; [12]SES, socioeconomic status; [13]EOPC, early-onset pancreatic cancer; [14]VEOPC, very early-onset pancreatic cancer; [15]Q, quartile; [16]PA, physical activity; [17]NSAID, non-steroidal anti-inflammatory drugs; [18]HBV, hepatitis B virus; [19]HCV, hepatitis C virus; [21]SIR, standardized incidence ratio.

Supplementary table 2 contains ecological, observational and interventional studies addressing internal exposures in PCa.

Author, Year	Country	Study Design	Population	Exposure	Findings	Observations
Risch, 2012 (231)	USA	Meta-analysis	22 studies	ABO Group	(+) PCa[1] risk: Compared to group O, ABO group A (OR[2] 1.40; 95%CI 1.28-1.53); ABO group B (OR 1.19; 95%CI 1.05-1.35); ABO group AB (OR 1.29; 95%CI[3] 1.10-1.51)	(+) Risk PCa in endemic populations for CagA-positive H. Pylori associated with ABO group A
Antwi, 2018 (54)	USA	Meta-Analysis	11 studies (PanC4 and PanScan consortiums)	ABO Group	(+) PCa risk: non-O groups in PanC4 studies (OR 1.28; 95%CI 1.13-1.44); non-O groups in PanScan studies (OR 1.36; 95%CI 1.07-1.75)	Both adjusted for age, sex, race, personal history of DM[4], family history of PCa, BMI[5], and smoking
Li, 2018 (162)	China	Case-Control	Cases (n=264), controls (n=423)	ABO Group	(+) Pca risk: ABO group A (OR 2.13; 95%CI 1.41-3.22); ABO group AB (OR 2.38; 95%CI 1.31-4.32)	Adjusted for sex, age, family history of PCa, smoking, history of DM, chronic pancreatitis, hepatitis B infection, and alcohol intake.
Risch 2010 (230)	USA	Case-Control	Cases (n=373), Controls (n=690)	ABO Group	(+) PCa risk: Non-O-blood group (OR1.37; 95%CI 1.02-1.83)	Adjusted for age, sex, and smoking
Wolpin, 2010 (232)	USA	Meta-Analysis	12 Studies	ABO Group	(+) PCa risk: Compared to OO, subjects with AO and AA (ORs of 1.33 (95% CI, 1.13-1.58) and 1.61 (95% CI, 1.22-2.18)); subjects with BO and BB (ORs of 1.45 (95% CI, 1.14-1.85) and 2.42 (1.28-4.57)); Non-O + Smoking (OR 2.68; 95%CI 2.03-3.54)	

Wolpin, 2009 (155)	USA	Cohort	PCa cases (n=316)	ABO Group	(+) Pca risk: compared with group O- group A (HR[6] 1.32; 95%CI 1.02-1.72); group AB (HR 1.51; 95%CI 1.02-2.23); group B (HR 1.72; 95%CI 1.25-2.38)	Adjusted for age, BMI, PA[5], smoking, history of DM.
Molina-Montes, 2018 (28)	Spain	Case-Control	Cases (n=1,431), Controls (n=1,090)	Diabetes Mellitus	(+) PCa risk: Diabetes Mellitus (OR 1.24; 95%CI 1.01-1.52)	
Li, 2018 (162)	China	Case-Control	Cases (n=264), Controls (n=423)	Diabetes Mellitus	(+) PCa risk: Diabetes Mellitus (OR 10.93; 95%CI 1.20-99.41)	Adjusted for sex, age, family history of pancreatic cancer, history of smoking, DM, chronic pancreatitis, chronic hepatitis B infection, alcohol drinking, and ABO blood type.
Er, 2016 (153)	China	Cohort	PCa cases (n=114)	Diabetes Mellitus	(+) PCa risk: Diabetes Mellitus (HR 2.53; 95%CI 1.96-3.26); poor glycaemic control (HR 3.61; 95%CI 1.34-9.78)	Adjusted for age, sex, SES[9], cardiovascular disease, smoking, other cancers, COPD[7], BMI[8], alcohol intake, and comorbidity index
Ben, 2011 (152)	China	Meta-Analysis	35 Studies	Diabetes Mellitus	(+) PCa risk: Diabetes Mellitus (RR 1.94; 95%CI 1.66-2.27)	
Pang, 2017 (154)	China	Meta-Analysis	22 Studies	Diabetes Mellitus	(+) PCa risk: Diabetes Mellitus (HR 1.87; 95%CI 1.48-2.37) with longer duration since diagnosis at higher risk; each 1mmol/L higher PG[10] (HR 1.12; 95%CI 1.04-1.21)	Adjusted for age, ethnicity, sex, alcohol intake, smoking, BMI, and FH[11] of PCa
Elena, 2013 (164)	USA	Meta-Analysis	12 Studies	Diabetes Mellitus	(+) PCa risk: Diabetes Mellitus (OR 1.40; 95%CI 1.07-1.84); risk was highest for duration 2-8 years (OR 1.79; 95%CI 1.25-2.55)	Adjusted for age, ethnicity, sex, alcohol intake, smoking, BMI, and FH of PCa
Song, 2015 (163)	China	Meta-Analysis	44 Studies	Diabetes Mellitus	(+) PCa risk: >2yrs Diabetes Mellitus (RR 1.64; 95%CI 1.52-1.78); >5yrs (RR[12] 1.58; 95%CI 1.42-1.75); >10yrs (RR .50; 95%CI 1.28-1.75)	
Bosetti, 2014 (169)	Italy	Meta-Analysis	15 Studies	Diabetes Mellitus	(+) PCa risk: >2 yrs Diabetes Mellitus (OR 1.90; 95%CI 1.72-2.09); >20 yrs (OR 1.30; 95%CI 1.03-1.63)	Adjusted for study, center (for multicenter studies), age, sex, race/ethnicity, education, body mass index, tobacco smoking, alcohol drinking, and history of pancreatitis.
Austin, 2013 (156)	USA	Case-Control	Cases (n=654), Controls (n=697)	Diabetes Mellitus	(+) PCa risk: first-degree FH of DM (OR 1.37; 95%CI 1.10-1.71); offspring with DM (OR 1.95; 95%CI 1.23-3.09)	Adjusted for sex, age, race, hispanic ethnicity
Ben, 2011 (152)	China	Case-Control	Cases (n=1,458), Controls (n=1,528)	Diabetes Mellitus	(+) PCa risk: >2yrs Diabetes Mellitus (OR 2.11; 95%CI 1.51-2.94); <2yrs Diabetes Mellitus (OR 4.43; 95%CI 3.44-5.72)	
Wang, 2006 (161)	USA	Case-Control	Cases (n=532), Controls (n=1,701)	Diabetes Mellitus	(+) PCa risk: history of Diabetes Mellitus (OR 1.5; 95%CI 1.1-2.1); 1-4 years (OR 2.4; 95%CI	

					1.4-4.0), 5-9 yrs (OR 2.0; 95%CI 1.2-3.4); Insulin use (OR 6.8; 95%CI 3.7-12)	
Setiawan, 2019 (160)	USA	Cohort	PCa cases (n=408)	Diabetes Mellitus	(+) PCa risk: DM (HR 2.39; 95%CI 1.91-2.98); recent-onset DM (<3yrs of Pca) in latinos (HR 4.08; 95%CI 2.76-6.03) and in african americans (HR 3.38; 95%CI 2.30-4.98)	Adjusted for BMI, smoking, alcohol intake, and red meat intake
Tseng, 2017 (244)	China	Cohort	PCa cases (n=16)	Diabetes Mellitus	No significant associations	
Karp, 2019 (170)	UK	Cohort	Incretin Users (n=18,885)	Diabetes Mellitus	No significant associations	
Currie, 2009 (167)	UK	Cohort		Diabetes Mellitus	(+) PCa risk: insulin-based therapies (HR 4.63; 95%CI 2.64-8.10)	
Raz, 2014 (173)	USA	Randomized Controlled Trial	Drug (n=5), Placebo (n=12)	Diabetes Mellitus	No significant associations	
Buse, 2017 (171)	USA	Randomized Controlled Trial	Drug (n=9), Placebo (n=14)	Diabetes Mellitus	(-) PCa risk: Sitagliptin (DPP-4[13] inhibitor) (0.66; 95%CI 0.28-1.51	
Perrin, 2017 (157)	Israel	Cohort	PCa cases (n=54)	Gestational Diabetes	(+) PCa risk: Gestatinal Diabetes (RR 7.1; 95%CI 2.8-18.0)	Adjusted for age at first birth and birth weight
Bansal, 1995 (186)	USA	Case-Control	Cases (n=2,639), Controls (n=7,774)	Pancreatitis	(+) PCa risk: Any pancreatitis (OR 3.42; 95%CI 1.98-5.91); Chronic Pancreatitis (OR 2.23; 95%CI 1.43-3.49) -- Pancreatitis >7 years before PCa diagnosis (OR 2.04; 95%CI 1.53-2.72); > 3 years before PCa diagnosis (OR 2.14; 95%CI 1.68-2.72); >1 year before PCa diagnosis (OR 3.42; 95%CI 1.87-2.86)	
Ekbom, 1994 (179)	Sweden	Cohort	PCa cases (n=46)	Pancreatitis	(+) PCa risk: Chronic pancreatitis (SIR 3.8; 95% CI 1.4-8.2); >1 episode of acute pancreatitis (SIR[14] 4.8; 95% CI 1.9-9.9)	
Malka, 2002 (183)	France	Cohort	PCa cases (n=4)	Pancreatitis	(+) PCa risk: Chronic pancreatitis (SIR 19; 95%CI 5.2-48.8)	
Duell, 2009 (213)	USA	Meta-Analysis	10 Studies	Pancreatitis	(+) PCa risk: Pancreatitis >2yrs of PCa diagnosis (OR 2.71; 95%CI 1.96-3.74); within 2yrs of PCa diagnosis (OR 13.56; 95%CI 8.72-21.90); younger patients (<65yrs)+ history pancreatitis (OR 3.91; 95%CI 2.53-6.04) than older (>65yrs))+ history pancreatitis (OR 1.68; 95%CI 1.02-2.76)	Adjusted for study, age, sex, ethnicity, education, BMI, smoking, alcohol intake and history of DM
Bracci, 2009 (187)	USA	Pooled Analysis	Cases (n=1,659), Controls (n=2,815)	Pancreatitis	(+) Pca risk: Pancreatitis (OR 7.2; 95%CI 4.0-13); patients <55 yrs (OR 9.9; 95%CI 3.5-28); < 3yrs	Adjusted for age, sex, ethnicity, smoking, alcohol intake, history of DM, and gallbladder disease

					after diagnosis of pancreatitis (OR 29; 95%CI 12-71); 3-10yrs (OR 2.6; 95%CI 1.5-5.6).	
Fernandez, 1995 (181)	Spain	Case-Control	Cases (n=362), Controls (n=1,408)	Pancreatitis	(+) Pca risk: Pancreatitis (RR 5.7; 95% CI 2.9-11.4); >5yrs after diagnosis of pancreatitis (RR 6.9; 95%CI 2.4-21.4); patients <60 yrs (RR 8.3; 95%CI 3.6-20.1)	Adjusted for age and sex
Talamini, 1999 (189)	Italy	Cohort	PCa cases (n=14)	Pancreatitis	(+) Pca risk: >4 yrs after diagnosis of pancreatitis (SIR 18.4; 95%CI 10-30); pancreatitis within 4yrs (SIR 13.3; 95%CI 6.4-24.5)	Adjusted for age and sex
Ueda, 2013 (190)	Japan	Cohort	PCa cases (n=19)	Pancreatitis	(+) PCa risk: Chronic pancreatitis (HR 11.8; 95%CI 7.1-18.4); patients who continued to drink alcohol after pancreatitis diagnosis (HR 5-07; 95%CI 1.13-22.73) / (-) PCa risk: patients who received surgery (HR 0.11; 95%CI 0.01-0.80)	Surgery: drainage operation or resection of pancreas
Karlson, 1997 (188)	Sweden	Cohort	PCa cases (n=472)	Pancreatitis	(+) PCa risk: Pancreatitis (SIR 2.8; 95%CI 2.5-3.2); pancreatitis diagnosed within 4 years (SIR 5.0; 95%CI 4.1-6.1); 5-9 yrs (SIR 2.5; 95%CI 2.0-3.1); >10 yrs (SIR 1.5; 95%CI 1.1-2.0)	Risk declined with time, except for patients with alcohol use after pancreatitis diagnosis
Lowenfels, 1993 (182)	USA	Cohort	PCa cases (n=56)	Pancreatitis	(+) PCa risk: Chronic pancreatitis (SIR 26.3; 95%CI 19.9-34.2)	Adjusted for age and sex
Fernandez, 1996 (127)	Italy	Case-Control	Cases (n=362), Controls (n=1,408)	Pancreatitis	(+) PCa risk: Pancreatitis (OR 3.4; 95%CI 1.7-6.8)	Adjusted for sex, area of residence, education, smoking, high meat intake, high fruit intake, and FH of PCa
Zheng, 2019 (245)	China	Cohort	PCa cases (n=12)	Pancreatitis	(+) PCa risk: Surgery for pancreatitis (HR 3.71; 95%CI 1.05-6.37)	
Andersson, 2018 (210)	Sweden	Cohort	PCa cases (n=110)	Hormonal and Reproductive Factors	(+) PCa risk: higher age at menarche (HR 1.17; 95%CI 1.04-1.32) / (-) PCa risk: Use of HRT[15] (HR 0.48; 95%CI 0.23-1.00); Estrogen-only (HR 0.22; 95%CI 0.05-0.90)	Adjusted for age, smoking, alcohol consumption and BMI
Lucenteforte, 2011 (206)	Italy	Pooled Analysis	Cases (n=285), Controls (n=713)	Hormonal and Reproductive Factors	(-) PCa risk: Multiparity >4 births (OR 0.46; 95%CI 0.26-0.85)	
Skinner, 2003 (207)	USA	Cohort	PCa cases (n=243)	Hormonal and Reproductive Factors	(-) PCa risk: Multiparity >5 births (HR 0.58; 95%CI 0.34-0.98)	Adjusted for smoking, BMI, history of DM, and height

Lo, 2007 (38)	Egypt	Case-Control	Cases (n=194), Controls (n=194)	Hormonal and Reproductive Factors	(-) PCa risk: Long period of lactation (OR 0.2; 95%CI 0.1-0.9)	Adjusted for age, sex and residence
Zhang, 2010 (208)	USA	Case-Control	Cases (n=284), Controls (n=1,096)	Hormonal and Reproductive Factors	(+) PCa risk: Older age at first pregnancy >30 yrs (OR 2.0; 95%CI 1.1-3.3); Oral contraceptive use >10 yrs (OR 1.0; 95%CI 1.0-4.0)	Adjusted for age, study center, year of interview, and ethnicity
Kreiger, 2001 (205)	Canada	Case-Control	Cases (n=52), Controls (n=233)	Hormonal and Reproductive Factors	(-) PCa risk: Multiparity >3 births (OR 0.22; 95%CI 0.07-0.65); Oral contraceptive use (OR 0.36; 95%CI 0.13-0.96) / (+) PCa risk: Older age at pregnancy >30 yrs (OR 3.78; 95%CI 1.02-14.06)	
Duell, 2005 (213)	USA	Case-Control	Cases (n=241), Controls (n=818)	Hormonal and Reproductive Factors	(+) PCa risk: Older age at menopause >45 (OR 1.80; 95%CI 1.2-2.8); Never use of oral contraceptives of HRT (OR 11.5; 95%CI 3.5-38.1)	Adjusted for smoking
Bueno de Mesquita, 1992 (211)	The Netherlands	Case-Control	Cases (n=176), Controls (n=487)	Hormonal and Reproductive Factors	(+) PCa risk: Early menarche (OR 3,07; 95%CI 1.35-7.0)	
Fernandez, 1995 (212)	Italy	Case-Control	Cases (n=133), Controls (n=377)	Hormonal and Reproductive Factors	(-) PCa risk: Early age at first birth (OR 0.5; 95%CI 0.3-0.9); (+) PCa risk: early menarche (OR 1,9; 95%CI 1.0-3.6)	
Karlsson, 1998 (209)	Sweden	Case-Control	Cases (n=1,015), Controls (n=5,073)	Hormonal and Reproductive Factors	(-) PCa risk: age at first birth (OR per 5 years 0.90; 95%CI 0.83-0.97);	
Zheng, 2016 (130)	China	Case-Control	Cases (n=323), Controls (n=323)	Obesity	(+) PCa risk: Obesity (BMI ≥30)(OR 1.77; 95%CI 1.22-2.57)	Adjusted for age, sex, race, residential areas, smoking, tea drinking, mental pressure, family history of pancreatic cancer, diabetes, gallstone, pickle consumption, and vegetable consumption.
Stolzenberg-Solomon, 2013 (215)	USA	Cohort	PCa cases (n=2,122)	Obesity	(+) PCa risk: Overweight/Obesity (BMI ≥25) (HR 1.06; 95%CI 1.02-1.09); overweight/obesity and DM (HR 1.18; 95%CI 1.05-1.30)	Adjusted for sex, smoking and energy-adjusted total fat
Larsson, 2007 (216)	Sweden	Meta-Analysis	21 Studies	Obesity	(+) PCa risk: per 5kg/m2 increase in BMI (RR 1.12; 95%CI 1.06-1.17); men (RR 1.16; 95%CI 1.05.-1.28); women (RR 1.10; 95%CI 1.02-1.19)	
Berrington, 2003 (214)	UK	Meta-Analysis	14 Studies	Obesity	(+) PCa risk: Obesity (BMI ≥30) (RR 1.19; 95%CI 1.10-1.29)	

Klein, 2013 (45)	USA	Case.Control	Cases (n=3,349), Controls (n=3,654)	Obesity	(+) PCa risk: Obesity (BMI ≥30) (OR 1.26; 95%CI 1.09-1.45)	
Jiao, 2010 (217)	USA	Meta-Analysis	7 Studies	Obesity	(+) PCa risk: per 5kg/m2 increase in BMI (RR 1.12; 95%CI 1.05-1.19); overweight (RR 1.13; 95%CI 1.03-1.23) and obesity (RR 1.19; 95%CI 1.05-1.35)	Adjusted for age and sex
Arslan, 2010 (165)	USA	Case-Control	Cases (n=2,170), Controls (n=2,209)	Obesity	(+) Pca risk: BMI Q4 vs Q1 (OR 1.33; 95%CI 1.12-1.58); men Q4 vs Q1 (OR 1.33; 95%CI 1.04-1.69); women Q4 vs Q1 (OR 1.34; 95%CI 1.05-1.70)	
McWilliams, 2016 (47)	USA	Meta-Analysis	8 Studies	Obesity	(+) PCa risk: Obesity (BMI ≥30) (OR 1.28; 95%CI 1.08-1.52)	Adjusted for FH of DM, sex, smoking, alcohol intake, and study site
Noor, 2016 (218)	UK	Cohort	PCa cases (n=88)	Physical Activity	No associations with overall PA / (-) PCa risk: patients <60yrs with high PA (HR 0.27; 95%CI 0.07-0.99)	
Rosato, 2011 (221)	Italy	Cohort	PCa cases (n=326)	Metabolic Syndrome	(+) PCa risk: Metabolic Syndrome (OR 2.13; 95%CI 1.01-4.49)	
Inoue, 2009 (229)	Japan	Cohort	PCa cases (n=1,858)	Metabolic Syndrome	(+) PCa risk: Metabolic Syndrome (HR 1.99; 95%CI 1.00-3.96)	Adjusted for age, smoking, study area, alcohol intake, serum cholesterol
Kabat, 2018 (219)	USA	Cohort	PCa cases (n=156)	Dyslipidaemias	(-) PCa risk: High HDL Q4 vs. Q1 (HR 0.52; 95%CI 0.32-0.85)	Adjusted for age, smoking, alcohol intake, BMI, physical activity, aspirin use, education, and ethnicity
La Torre, 2014 (220)	Italy	Case-Control	Cases (n=80), Controls (n=392)	Dyslipidaemias	(+) PCa risk: High TC (OR 5.05; 95%CI 2.94-8.66)	Adjusted for sex, age, smoking and alcohol consumption
Kirkegard, 2020 (227)	Denmark	Cohort	PCa cases (n=153)	Statin Use	No significant associations	Adjusted for age, sex, SES and comorbidity index score
Chiu, 2011 (225)	China	Case-Control	Cases (n=190), Controls (n==760)	Statin Use	No significant associations	Adjusted for DM, chronic pancreatitis, number of hospitalizations, and use of other lipid-lowering drugs
Cui, 2012 (226)	China	Meta-Analysis	16 Studies	Statin Use	No significant associations	
Bradley, 2010 (223)	UK	Case-Control	Cases (n=1,141), Controls (n=7,954)	Statin Use	No significant associations	Adjusted for smoking, BMI, alcohol, history of chronic pancreatitis, use of other drugs (steroids and HRT), DM and prior cancer
Carey, 2013 (224)	UK	Case-Control	Cases (n=252), Controls (n=504)	Statin Use	No significant associations	Adjusted for smoking and DM

Archibugi, 2017 (83)	Italy	Case-Control	Cases (n=408), Controls (n=816)	Statin Use	(-) PCa risk: Statin use (OR 0.61; 95%CI 0.43-0.88)	Adjusted for age, sex, BMI, FH of Pca, history of CP[18], DM, smoking, alcohol use
Molina-Montes, 2018 (28)	Spain	Case-Control	Cases (n=1,431), Controls (n=1,090)	Family History of PCa	(+) PCa risk: FH of PCa (OR 3.88; 95%CI 2.96-9.73)	
Hamada, 2019 (132)	USA	Cohort	PCa cases (n=452)	Family History of PCa	(+) PCa risk: FH of PCa (HR 2.79; 1.28-6.07)	Adjusted for age, ethnicity, calendar year of questionnaire cycle, smoking, history of DM, BMI, physical activity, alcohol intake, and regular multivitamin use
Schulte, 2016 (131)	Australia	Case-Control	Cases (n=591), Controls (n=646)	Family History of PCa	(+) PCa risk: FH of PCa (OR 2.20; 95%CI 1.16-4.19)	Adjusted for age, sex, education and smoking status
Zheng, 2016 (130)	China	Case-Control	Cases (n=323), Controls (n=323)	Family History of PCa	(+) PCa risk: FH of PCa (OR 1.23; 95%CI 1.11-3.70)	Adjusted for age, sex, ethnicity, residential area, smoking, tea consumption, BMI, DM, gallstones, pickle and vegetable consumption
Paiella, 2019 (128)	Italy	Cohort	PCa cases (n=187)	Family History of PCa	(+) PCa risk: FH of PCa (OR 2.7; 95%CI 1.1-6.4)	
Matsubayashi, 2011 (27)	Japan	Case-Control	Cases (n=577), Controls (n=577)	Family History of PCa	(+) PCa risk: FH of PCa (OR 2.5; 95%CI 1.37-4.38)	
Fernandez, 1996 (127)	Italy	Case-Control	Cases (n=362), Controls (n=1,408)	Family History of PCa	(+) Pca risk: FH of PCa (OR 4.7; 95%CI 1.5-15.1)	Adjusted for age, area of residence and education
Schenk, 2001 (126)	USA	Case-Control	Cases (n=247), Controls (n=420)	Family History of PCa	(+) PCa risk: FH of PCa (RR 2.49; 95%CI 1.32-4.69)	Adjusted for age, ethnicity, smoking, and history of DM
Zhan, 2013 (125)	China	Case-Control	Cases (n=196), Controls (n=233)	Family History of PCa	(+) PCa risk: FH of PCa (OR 16.75; 95%CI 2.12-132.05)	
McWilliams, 2016 (47)	USA	Meta-Analysis	8 Studies	Family History of PCa	(+) EOPC[19] risk: FH of PCa (OR 2.53; 95%CI 1.77-3.61) / (+) VEOPC[20] risk: FH of PCa (OR 2.88; 95%CI 1.04-7.99)	
Fernandez, 1994 (123)	Spain	Case-Control	Cases (n=362), Controls (n=1,408)	Family History of PCa	(+) PCa risk: FH of PCa (RR 3.0; 95%CI 1.4-6.6)	

Klein, 2003 (133)	USA	Cohort	PCa cases (n=19)	Family History of PCa	(+) PCa risk: One first-degree relative (SIR 4.5; 95%CI 0.54-16.3); Two first-degree relatives (SIR 6.4; 95%CI 1.8-16.4); three or more first-degree relatives (SIR 32.0; 95%CI 10.4-74.7)	Adjusted for age, sex, and race
Sollie, 2019 (176)	UK	Cohort	PCa cases (n=286)	Inflammatory Markers	(+) PCa risk: High haptoglobin (HR2.23; 95%CI 1.72-2.88); CRP[21] (HR 1.32; 95%CI 1.00-1.74); leukocytes (HR 2.20; 95%CI 1.52-3.18)	Adjusted for age, gender, education, CCI and serum glucose
Grote, 2012 (177)	Germany	Case-Control	Cases (n=455), Controls (n=455)	Inflammatory Markers	(+) PCa risk: TNF-a[22] Q4 vs. Q1 (OR 1.97; 95%CI 1.02-3.79) in women / No other associations	
Moran, 2012 (136)	UK	Cohort	PCa cases (n=220)	Genetic Factors	(+) PCa risk: BRCA2 (RR 4.1; 95%CI 1.9-7.8) / No significant associations with BRCA1	
Bannon, 2018 (138)	USA	Cohort	PCa cases (n=584)	Genetic Factors	(+) EOPC risk: BRCA1 and BRCA2 (OR 1.93; 95%CI 1.03-3.70)	
Mersch, 2015 (137)	USA	Cohort	PCa cases (n=23)	Genetic Factors	(+) PCa risk: BRCA2 (SIR 21.7; 95%CI 13.1-34.0) / No significant associations with BRCA1	
Roberts, 2012 (140)	USA	Case-Control	Cases (n=166), Controls (n=190)	Genetic Factors	(+) PCa risk: ATM mutation (4/166 vs. 0/190, Fisher's Exact Test P=0.046)	
Lynch, 2005 (143)	USA	Cohort	PCa cases (n=19)	Genetic Factors	(+) PCa risk: BRCA1 and BRCA2	
Giardiello, 2000 (146)	USA	Meta-Analysis	6 Studies	Genetic Factors	(+) PCa risk: Peutz-Jeghers Syndrome (STK11 mutation) (RR 132; 95%CI 44.261)	
Rebours, 2008 (149)	France	Cohort	PCa cases (n=200)	Genetic Factors	(+) PCa risk: PRSS1 mutation (OR 87; 95%CI 42-113)	Adjusted for age and sex
Howes, 2003 (148)	UK	Cohort	PCa cases (n=418)	Genetic Factors	(+) PCa risk: PRSS1 mutation (OR 44; 95%CI 8-88)	
Vasen, 2000 (144)	The Netherlands	Cohort	PCa cases (n=15)	Genetic Factors	(+) PCa risk: Familial atypical multiple mole melanoma (CDKN2A mutation) (OR 17; 95%CI 13-30)	

Kastrinos, 2009 (246)	USA	Cohort	PCa cases (n=147)	Genetic Factors	(+) PCa risk: Lynch syndrome (MLH1, MSH2, MSH6 mutations)(RR 3.7; 95%CI 1.45-5.88)
Aarnio, 1999 (151)	Finland	Cohort	PCa cases (n=360)	Genetic Factors	(+) PCa risk: Lynch Syndrome (MSH2, MLH1, PMS1, PMS2 or MSH6)(SIR 4.5; 95%CI 1.0-13)
Tada, 2006 (233)	Japan	Cohort	PCa cases (n=197)	Cystic Lesions	(+) PCa risk: Pancreatic cystic lesions (SIR 22.5; 95%CI 11.0-45.3)
Matsubara, 2012 (234)	Japan	Case-Control	Cases (n=116), Controls (n=1,226)	Cystic Lesions	(+) PCa risk: Pancreatic cystic lesion (OR 10.27; 95%CI 6.76-15.73); Size >10mm (OR 4.71; 95%CI 2.21-10.69)

[1]PCa, pancreatic cancer; [2]OR, odds ratio; [3]CI, confidence interval; [4]DM, diabetes mellitus; [5]PA, physical activity; [6]HR, hazard ratio; [7]COPD, chronic obstructive pulmonary disease; [8]BMI, body mass index; [9]SES, socioeconomic status; [10]PG, plasma Glucose; [11]FH, family history; [12]RR, relative risk; [13]Dipeptidyl peptidase-4; [14]SIR, standardized incidence ratio; [15]HRT, hormone replacement therapy; [16]HDL, HDL-cholesterol; [17]TC, total cholesterol; [18]CP, chronic pancreatitis; [19]EOPC, early-onset pancreatic cancer; [20]VEOPC, very early-onset pancreatic cancer; [21]CRP, C-reactive protein; [22]TNF-a, tumour necrosis factor alfa.

References

1. Rawla P, Sunkara T, Gaduputi V. Epidemiology of Pancreatic Cancer: Global Trends, Etiology and Risk Factors. World J Oncol. 2019;10(1):10-27.
2. Sung H, Ferlay J, Siegel RL, Laversanne M, Soerjomataram I, Jemal A, et al. Global cancer statistics 2020: GLOBOCAN estimates of incidence and mortality worldwide for 36 cancers in 185 countries. CA Cancer J Clin. 2021.
3. Mizrahi JD, Surana R, Valle JW, Shroff RT. Pancreatic cancer. Lancet. 2020;395(10242):2008-20.
4. Yeo TP. Demographics, epidemiology, and inheritance of pancreatic ductal adenocarcinoma. Semin Oncol. 2015;42(1):8-18.
5. Kong B, Michalski CW, Erkan M, Friess H, Kleeff J. From tissue turnover to the cell of origin for pancreatic cancer. Nat Rev Gastroenterol Hepatol. 2011;8(8):467-72.
6. PanCan. Types of Pancreatic Cancer 2020 [cited 2021 Fabruary]. Available from: https://www.pancan.org/facing-pancreatic-cancer/about-pancreatic-cancer/types-of-pancreatic-cancer/.
7. Midha S, Chawla S, Garg PK. Modifiable and non-modifiable risk factors for pancreatic cancer: A review. Cancer Lett. 2016;381(1):269-77.
8. Capasso M, Franceschi M, Rodriguez-Castro KI, Crafa P, Cambie G, Miraglia C, et al. Epidemiology and risk factors of pancreatic cancer. Acta Biomed. 2018;89(9-S):141-6.
9. Guertin KA, Freedman ND, Loftfield E, Stolzenberg-Solomon RZ, Graubard BI, Sinha R. A prospective study of coffee intake and pancreatic cancer: results from the NIH-AARP Diet and Health Study. Br J Cancer. 2015;113(7):1081-5.
10. Neoptolemos JP, Palmer DH, Ghaneh P, Psarelli EE, Valle JW, Halloran CM, et al. Comparison of adjuvant gemcitabine and capecitabine with gemcitabine monotherapy in patients with resected pancreatic cancer (ESPAC-4): a multicentre, open-label, randomised, phase 3 trial. Lancet. 2017;389(10073):1011-24.
11. Zheng J, Guinter MA, Merchant AT, Wirth MD, Zhang J, Stolzenberg-Solomon RZ, et al. Dietary patterns and risk of pancreatic cancer: a systematic review. Nutr Rev. 2017;75(11):883-908.
12. Bosetti C, Bertuccio P, Negri E, La Vecchia C, Zeegers MP, Boffetta P. Pancreatic cancer: overview of descriptive epidemiology. Mol Carcinog. 2012;51(1):3-13.
13. Wild CP. Complementing the genome with an "exposome": the outstanding challenge of environmental exposure measurement in molecular epidemiology. Cancer Epidemiol Biomarkers Prev. 2005;14(8):1847-50.
14. Vineis P, Robinson O, Chadeau-Hyam M, Dehghan A, Mudway I, Dagnino S. What is new in the exposome? Environ Int. 2020;143:105887.
15. Vineis P, Chadeau-Hyam M, Gmuender H, Gulliver J, Herceg Z, Kleinjans J, et al. The exposome in practice: Design of the EXPOsOMICS project. Int J Hyg Environ Health. 2017;220(2 Pt A):142-51.
16. Wild CP. The exposome: from concept to utility. Int J Epidemiol. 2012;41(1):24-32.
17. Mohammed SH, Habtewold TD, Birhanu MM, Sissay TA, Tegegne BS, Abuzerr S, et al. Neighbourhood socioeconomic status and overweight/obesity: a systematic review and meta-analysis of epidemiological studies. BMJ Open. 2019;9(11):e028238.
18. Hsing AW, Sakoda LC, Chua S, Jr. Obesity, metabolic syndrome, and prostate cancer. Am J Clin Nutr. 2007;86(3):s843-57.
19. Robinson O, Vrijheid M. The Pregnancy Exposome. Curr Environ Health Rep. 2015;2(2):204-13.
20. Daiber A, Lelieveld J, Steven S, Oelze M, Kroller-Schon S, Sorensen M, et al. The "exposome" concept - how environmental risk factors influence cardiovascular health. Acta Biochim Pol. 2019;66(3):269-83.
21. Dreno B, Bettoli V, Araviiskaia E, Sanchez Viera M, Bouloc A. The influence of exposome on acne. J Eur Acad Dermatol Venereol. 2018;32(5):812-9.
22. Pham A, Forsmark C. Chronic pancreatitis: review and update of etiology, risk factors, and management. F1000Res. 2018;7.
23. Moher D, Liberati A, Tetzlaff J, Altman DG, Group P. Preferred reporting items for systematic reviews and meta-analyses: the PRISMA statement. PLoS Med. 2009;6(7):e1000097.
24. Antwi SO, Oberg AL, Shivappa N, Bamlet WR, Chaffee KG, Steck SE, et al. Pancreatic cancer: associations of inflammatory potential of diet, cigarette smoking and long-standing diabetes. Carcinogenesis. 2016;37(5):481-90.

25. Hassan MM, Abbruzzese JL, Bondy ML, Wolff RA, Vauthey JN, Pisters PW, et al. Passive smoking and the use of noncigarette tobacco products in association with risk for pancreatic cancer: a case-control study. Cancer. 2007;109(12):2547-56.
26. Korc M, Jeon CY, Edderkaoui M, Pandol SJ, Petrov MS, Consortium for the Study of Chronic Pancreatitis D, et al. Tobacco and alcohol as risk factors for pancreatic cancer. Best Pract Res Clin Gastroenterol. 2017;31(5):529-36.
27. Matsubayashi H, Maeda A, Kanemoto H, Uesaka K, Yamazaki K, Hironaka S, et al. Risk factors of familial pancreatic cancer in Japan: current smoking and recent onset of diabetes. Pancreas. 2011;40(6):974-8.
28. Molina-Montes E, Gomez-Rubio P, Marquez M, Rava M, Lohr M, Michalski CW, et al. Risk of pancreatic cancer associated with family history of cancer and other medical conditions by accounting for smoking among relatives. Int J Epidemiol. 2018;47(2):473-83.
29. Schulte A, Pandeya N, Tran B, Fawcett J, Fritschi L, Risch HA, et al. Cigarette smoking and pancreatic cancer risk: more to the story than just pack-years. Eur J Cancer. 2014;50(5):997-1003.
30. Vrieling A, Bueno-de-Mesquita HB, Boshuizen HC, Michaud DS, Severinsen MT, Overvad K, et al. Cigarette smoking, environmental tobacco smoke exposure and pancreatic cancer risk in the European Prospective Investigation into Cancer and Nutrition. Int J Cancer. 2010;126(10):2394-403.
31. Kuzmickiene I, Everatt R, Virviciute D, Tamosiunas A, Radisauskas R, Reklaitiene R, et al. Smoking and other risk factors for pancreatic cancer: a cohort study in men in Lithuania. Cancer Epidemiol. 2013;37(2):133-9.
32. Wang Y, Duan H, Yang X, Guo J. Cigarette smoking and the risk of pancreatic cancer: a case-control study. Med Oncol. 2014;31(10):184.
33. Heinen MM, Verhage BA, Goldbohm RA, van den Brandt PA. Active and passive smoking and the risk of pancreatic cancer in the Netherlands Cohort Study. Cancer Epidemiol Biomarkers Prev. 2010;19(6):1612-22.
34. Brand RE, Greer JB, Zolotarevsky E, Brand R, Du H, Simeone D, et al. Pancreatic cancer patients who smoke and drink are diagnosed at younger ages. Clin Gastroenterol Hepatol. 2009;7(9):1007-12.
35. Muscat JE, Stellman SD, Hoffmann D, Wynder EL. Smoking and pancreatic cancer in men and women. Cancer Epidemiol Biomarkers Prev. 1997;6(1):15-9.
36. Nilsen TI, Vatten LJ. A prospective study of lifestyle factors and the risk of pancreatic cancer in Nord-Trondelag, Norway. Cancer Causes Control. 2000;11(7):645-52.
37. Hassan MM, Bondy ML, Wolff RA, Abbruzzese JL, Vauthey JN, Pisters PW, et al. Risk factors for pancreatic cancer: case-control study. Am J Gastroenterol. 2007;102(12):2696-707.
38. Lo AC, Soliman AS, El-Ghawalby N, Abdel-Wahab M, Fathy O, Khaled HM, et al. Lifestyle, occupational, and reproductive factors in relation to pancreatic cancer risk. Pancreas. 2007;35(2):120-9.
39. Chuang SC, Gallo V, Michaud D, Overvad K, Tjonneland A, Clavel-Chapelon F, et al. Exposure to environmental tobacco smoke in childhood and incidence of cancer in adulthood in never smokers in the European Prospective Investigation into Cancer and Nutrition. Cancer Causes Control. 2011;22(3):487-94.
40. Gallicchio L, Kouzis A, Genkinger JM, Burke AE, Hoffman SC, Diener-West M, et al. Active cigarette smoking, household passive smoke exposure, and the risk of developing pancreatic cancer. Prev Med. 2006;42(3):200-5.
41. Villeneuve PJ, Johnson KC, Mao Y, Hanley AJ, Canadian Cancer Registries Research G. Environmental tobacco smoke and the risk of pancreatic cancer: findings from a Canadian population-based case-control study. Can J Public Health. 2004;95(1):32-7.
42. Capurso G, Falconi M, Panzuto F, Rinzivillo M, Boninsegna L, Bettini R, et al. Risk factors for sporadic pancreatic endocrine tumors: a case-control study of prospectively evaluated patients. Am J Gastroenterol. 2009;104(12):3034-41.
43. Genkinger JM, Spiegelman D, Anderson KE, Bergkvist L, Bernstein L, van den Brandt PA, et al. Alcohol intake and pancreatic cancer risk: a pooled analysis of fourteen cohort studies. Cancer Epidemiol Biomarkers Prev. 2009;18(3):765-76.
44. Gupta S, Wang F, Holly EA, Bracci PM. Risk of pancreatic cancer by alcohol dose, duration, and pattern of consumption, including binge drinking: a population-based study. Cancer Causes Control. 2010;21(7):1047-59.

45. Klein AP, Lindstrom S, Mendelsohn JB, Steplowski E, Arslan AA, Bueno-de-Mesquita HB, et al. An absolute risk model to identify individuals at elevated risk for pancreatic cancer in the general population. PLoS One. 2013;8(9):e72311.
46. Lu XH, Wang L, Li H, Qian JM, Deng RX, Zhou L. Establishment of risk model for pancreatic cancer in Chinese Han population. World J Gastroenterol. 2006;12(14):2229-34.
47. McWilliams RR, Maisonneuve P, Bamlet WR, Petersen GM, Li D, Risch HA, et al. Risk Factors for Early-Onset and Very-Early-Onset Pancreatic Adenocarcinoma: A Pancreatic Cancer Case-Control Consortium (PanC4) Analysis. Pancreas. 2016;45(2):311-6.
48. Naudin S, Li K, Jaouen T, Assi N, Kyro C, Tjonneland A, et al. Lifetime and baseline alcohol intakes and risk of pancreatic cancer in the European Prospective Investigation into Cancer and Nutrition study. Int J Cancer. 2018;143(4):801-12.
49. Miyasaka K, Kawanami T, Shimokata H, Ohta S, Funakoshi A. Inactive aldehyde dehydrogenase-2 increased the risk of pancreatic cancer among smokers in a Japanese male population. Pancreas. 2005;30(2):95-8.
50. Talamini G, Bassi C, Falconi M, Sartori N, Salvia R, Rigo L, et al. Alcohol and smoking as risk factors in chronic pancreatitis and pancreatic cancer. Dig Dis Sci. 1999;44(7):1303-11.
51. Bouchardy C, Clavel F, La Vecchia C, Raymond L, Boyle P. Alcohol, beer and cancer of the pancreas. Int J Cancer. 1990;45(5):842-6.
52. Falk RT, Pickle LW, Fontham ET, Correa P, Fraumeni JF, Jr. Life-style risk factors for pancreatic cancer in Louisiana: a case-control study. Am J Epidemiol. 1988;128(2):324-36.
53. Shivappa N, Steck SE, Hurley TG, Hussey JR, Hebert JR. Designing and developing a literature-derived, population-based dietary inflammatory index. Public Health Nutr. 2014;17(8):1689-96.
54. Antwi SO, Bamlet WR, Pedersen KS, Chaffee KG, Risch HA, Shivappa N, et al. Pancreatic cancer risk is modulated by inflammatory potential of diet and ABO genotype: a consortia-based evaluation and replication study. Carcinogenesis. 2018;39(8):1056-67.
55. Larsson SC, Wolk A. Red and processed meat consumption and risk of pancreatic cancer: meta-analysis of prospective studies. Br J Cancer. 2012;106(3):603-7.
56. Shivappa N, Bosetti C, Zucchetto A, Serraino D, La Vecchia C, Hebert JR. Dietary inflammatory index and risk of pancreatic cancer in an Italian case-control study. Br J Nutr. 2015;113(2):292-8.
57. Bao Y, Nimptsch K, Wolpin BM, Michaud DS, Brand-Miller JC, Willett WC, et al. Dietary insulin load, dietary insulin index, and risk of pancreatic cancer. Am J Clin Nutr. 2011;94(3):862-8.
58. Lin Y, Tamakoshi A, Hayakawa T, Naruse S, Kitagawa M, Ohno Y. Nutritional factors and risk of pancreatic cancer: a population-based case-control study based on direct interview in Japan. J Gastroenterol. 2005;40(3):297-301.
59. Ji BT, Chow WH, Gridley G, McLaughlin JK, Dai Q, Wacholder S, et al. Dietary factors and the risk of pancreatic cancer: a case-control study in Shanghai China. Cancer Epidemiol Biomarkers Prev. 1995;4(8):885-93.
60. Wang J, Zhang W, Sun L, Yu H, Ni QX, Risch HA, et al. Dietary energy density is positively associated with risk of pancreatic cancer in urban Shanghai Chinese. J Nutr. 2013;143(10):1626-9.
61. Farrow DC, Davis S. Diet and the risk of pancreatic cancer in men. Am J Epidemiol. 1990;132(3):423-31.
62. Soler M, Chatenoud L, La Vecchia C, Franceschi S, Negri E. Diet, alcohol, coffee and pancreatic cancer: final results from an Italian study. Eur J Cancer Prev. 1998;7(6):455-60.
63. Anderson KE, Mongin SJ, Sinha R, Stolzenberg-Solomon R, Gross MD, Ziegler RG, et al. Pancreatic cancer risk: associations with meat-derived carcinogen intake in the Prostate, Lung, Colorectal, and Ovarian Cancer Screening Trial (PLCO) cohort. Mol Carcinog. 2012;51(1):128-37.
64. Jayedi A, Emadi A, Shab-Bidar S. Dietary Inflammatory Index and Site-Specific Cancer Risk: A Systematic Review and Dose-Response Meta-Analysis. Adv Nutr. 2018;9(4):388-403.
65. Lucenteforte E, Talamini R, Bosetti C, Polesel J, Franceschi S, Serraino D, et al. Macronutrients, fatty acids, cholesterol and pancreatic cancer. Eur J Cancer. 2010;46(3):581-7.

66. Ruan Y, Poirier AE, Hebert LA, Grevers X, Walter SD, Villeneuve PJ, et al. Estimates of the current and future burden of cancer attributable to red and processed meat consumption in Canada. Prev Med. 2019;122:31-9.

67. Zheng J, Merchant AT, Wirth MD, Zhang J, Antwi SO, Shoaibi A, et al. Inflammatory potential of diet and risk of pancreatic cancer in the Prostate, Lung, Colorectal and Ovarian (PLCO) Cancer Screening Trial. Int J Cancer. 2018;142(12):2461-70.

68. Zheng J, Wirth MD, Merchant AT, Zhang J, Shivappa N, Stolzenberg-Solomon RZ, et al. Inflammatory Potential of Diet, Inflammation-Related Lifestyle Factors, and Risk of Pancreatic Cancer: Results from the NIH-AARP Diet and Health Study. Cancer Epidemiol Biomarkers Prev. 2019;28(7):1266-70.

69. Asli LA, Braaten T, Olsen A, Tjonneland A, Overvad K, Nilsson LM, et al. Potato consumption and risk of pancreatic cancer in the HELGA cohort. Br J Nutr. 2018;119(12):1408-15.

70. Mueller NT, Odegaard A, Anderson K, Yuan JM, Gross M, Koh WP, et al. Soft drink and juice consumption and risk of pancreatic cancer: the Singapore Chinese Health Study. Cancer Epidemiol Biomarkers Prev. 2010;19(2):447-55.

71. Schernhammer ES, Hu FB, Giovannucci E, Michaud DS, Colditz GA, Stampfer MJ, et al. Sugar-sweetened soft drink consumption and risk of pancreatic cancer in two prospective cohorts. Cancer Epidemiol Biomarkers Prev. 2005;14(9):2098-105.

72. Nothlings U, Murphy SP, Wilkens LR, Henderson BE, Kolonel LN. Dietary glycemic load, added sugars, and carbohydrates as risk factors for pancreatic cancer: the Multiethnic Cohort Study. Am J Clin Nutr. 2007;86(5):1495-501.

73. Luo J, Inoue M, Iwasaki M, Sasazuki S, Otani T, Ye W, et al. Green tea and coffee intake and risk of pancreatic cancer in a large-scale, population-based cohort study in Japan (JPHC study). Eur J Cancer Prev. 2007;16(6):542-8.

74. Turati F, Galeone C, Talamini R, Franceschi S, Manzari M, Gallino G, et al. Coffee, decaffeinated coffee, tea, and pancreatic cancer risk: a pooled-analysis of two Italian case-control studies. Eur J Cancer Prev. 2011;20(4):287-92.

75. Banim PJ, Luben R, McTaggart A, Welch A, Wareham N, Khaw KT, et al. Dietary antioxidants and the aetiology of pancreatic cancer: a cohort study using data from food diaries and biomarkers. Gut. 2013;62(10):1489-96.

76. Lucas AL, Bosetti C, Boffetta P, Negri E, Tavani A, Serafini M, et al. Dietary total antioxidant capacity and pancreatic cancer risk: an Italian case-control study. Br J Cancer. 2016;115(1):102-7.

77. Waterhouse M, Risch HA, Bosetti C, Anderson KE, Petersen GM, Bamlet WR, et al. Vitamin D and pancreatic cancer: a pooled analysis from the Pancreatic Cancer Case-Control Consortium. Ann Oncol. 2015;26(8):1776-83.

78. Stolzenberg-Solomon RZ, Jacobs EJ, Arslan AA, Qi D, Patel AV, Helzlsouer KJ, et al. Circulating 25-hydroxyvitamin D and risk of pancreatic cancer: Cohort Consortium Vitamin D Pooling Project of Rarer Cancers. Am J Epidemiol. 2010;172(1):81-93.

79. Stolzenberg-Solomon RZ, Hayes RB, Horst RL, Anderson KE, Hollis BW, Silverman DT. Serum vitamin D and risk of pancreatic cancer in the prostate, lung, colorectal, and ovarian screening trial. Cancer Res. 2009;69(4):1439-47.

80. Chuang SC, Stolzenberg-Solomon R, Ueland PM, Vollset SE, Midttun O, Olsen A, et al. A U-shaped relationship between plasma folate and pancreatic cancer risk in the European Prospective Investigation into Cancer and Nutrition. Eur J Cancer. 2011;47(12):1808-16.

81. Gong Z, Holly EA, Bracci PM. Intake of folate, vitamins B6, B12 and methionine and risk of pancreatic cancer in a large population-based case-control study. Cancer Causes Control. 2009;20(8):1317-25.

82. Stolzenberg-Solomon RZ, Albanes D, Nieto FJ, Hartman TJ, Tangrea JA, Rautalahti M, et al. Pancreatic cancer risk and nutrition-related methyl-group availability indicators in male smokers. J Natl Cancer Inst. 1999;91(6):535-41.

83. Archibugi L, Piciucchi M, Stigliano S, Valente R, Zerboni G, Barucca V, et al. Exclusive and Combined Use of Statins and Aspirin and the Risk of Pancreatic Cancer: a Case-Control Study. Sci Rep. 2017;7(1):13024.

84. Bradley MC, Hughes CM, Cantwell MM, Napolitano G, Murray LJ. Non-steroidal anti-inflammatory drugs and pancreatic cancer risk: a nested case-control study. Br J Cancer. 2010;102(9):1415-21.

85. Coogan PF, Rosenberg L, Palmer JR, Strom BL, Zauber AG, Stolley PD, et al. Nonsteroidal anti-inflammatory drugs and risk of digestive cancers at sites other than the large bowel. Cancer Epidemiol Biomarkers Prev. 2000;9(1):119-23.

86. Schernhammer ES, Kang JH, Chan AT, Michaud DS, Skinner HG, Giovannucci E, et al. A prospective study of aspirin use and the risk of pancreatic cancer in women. J Natl Cancer Inst. 2004;96(1):22-8.

87. Khalaf N, Yuan C, Hamada T, Cao Y, Babic A, Morales-Oyarvide V, et al. Regular Use of Aspirin or Non-Aspirin Nonsteroidal Anti-Inflammatory Drugs Is Not Associated With Risk of Incident Pancreatic Cancer in Two Large Cohort Studies. Gastroenterology. 2018;154(5):1380-90 e5.
88. Xing S, Li ZW, Tian YF, Zhang LM, Li MQ, Zhou P. Chronic hepatitis virus infection increases the risk of pancreatic cancer: a meta-analysis. Hepatobiliary Pancreat Dis Int. 2013;12(6):575-83.
89. Wang Y, Yang S, Song F, Cao S, Yin X, Xie J, et al. Hepatitis B virus status and the risk of pancreatic cancer: a meta-analysis. Eur J Cancer Prev. 2013;22(4):328-34.
90. Song C, Lv J, Liu Y, Chen JG, Ge Z, Zhu J, et al. Associations Between Hepatitis B Virus Infection and Risk of All Cancer Types. JAMA Netw Open. 2019;2(6):e195718.
91. Chang MC, Chen CH, Liang JD, Tien YW, Hsu C, Wong JM, et al. Hepatitis B and C viruses are not risks for pancreatic adenocarcinoma. World J Gastroenterol. 2014;20(17):5060-5.
92. Tang J, Sharma R, Lamerato L, Sheehan M, Krajenta R, Gordon SC. Is previous exposure to hepatitis B a risk factor for pancreatic cancer or hepatocellular carcinoma? J Clin Gastroenterol. 2014;48(8):729-33.
93. Risch HA, Lu L, Kidd MS, Wang J, Zhang W, Ni Q, et al. Helicobacter pylori seropositivities and risk of pancreatic carcinoma. Cancer Epidemiol Biomarkers Prev. 2014;23(1):172-8.
94. Ai F, Hua X, Liu Y, Lin J, Feng Z. Preliminary study of pancreatic cancer associated with Helicobacter pylori infection. Cell Biochem Biophys. 2015;71(1):397-400.
95. de Martel C, Llosa AE, Friedman GD, Vogelman JH, Orentreich N, Stolzenberg-Solomon RZ, et al. Helicobacter pylori infection and development of pancreatic cancer. Cancer Epidemiol Biomarkers Prev. 2008;17(5):1188-94.
96. Hirabayashi M, Inoue M, Sawada N, Saito E, Abe SK, Hidaka A, et al. Helicobacter pylori infection, atrophic gastritis, and risk of pancreatic cancer: A population-based cohort study in a large Japanese population: the JPHC Study. Sci Rep. 2019;9(1):6099.
97. Lindkvist B, Johansen D, Borgstrom A, Manjer J. A prospective study of Helicobacter pylori in relation to the risk for pancreatic cancer. BMC Cancer. 2008;8:321.
98. Chen XZ, Schottker B, Castro FA, Chen H, Zhang Y, Holleczek B, et al. Association of helicobacter pylori infection and chronic atrophic gastritis with risk of colonic, pancreatic and gastric cancer: A ten-year follow-up of the ESTHER cohort study. Oncotarget. 2016;7(13):17182-93.
99. Gerlovin H, Michaud DS, Cozier YC, Palmer JR. Oral Health in Relation to Pancreatic Cancer Risk in African American Women. Cancer Epidemiol Biomarkers Prev. 2019;28(4):675-9.
100. Michaud DS, Joshipura K, Giovannucci E, Fuchs CS. A prospective study of periodontal disease and pancreatic cancer in US male health professionals. J Natl Cancer Inst. 2007;99(2):171-5.
101. Fan X, Alekseyenko AV, Wu J, Peters BA, Jacobs EJ, Gapstur SM, et al. Human oral microbiome and prospective risk for pancreatic cancer: a population-based nested case-control study. Gut. 2018;67(1):120-7.
102. Michaud DS, Izard J, Wilhelm-Benartzi CS, You DH, Grote VA, Tjonneland A, et al. Plasma antibodies to oral bacteria and risk of pancreatic cancer in a large European prospective cohort study. Gut. 2013;62(12):1764-70.
103. Caygill CP, Hill MJ, Braddick M, Sharp JC. Cancer mortality in chronic typhoid and paratyphoid carriers. Lancet. 1994;343(8889):83-4.
104. Gotland N, Uhre ML, Sandholdt H, Mejer N, Lundbo LF, Petersen A, et al. Increased risk of incident primary cancer after Staphylococcus aureus bacteremia: A matched cohort study. Medicine (Baltimore). 2020;99(17):e19984.
105. Bao Y, Spiegelman D, Li R, Giovannucci E, Fuchs CS, Michaud DS. History of peptic ulcer disease and pancreatic cancer risk in men. Gastroenterology. 2010;138(2):541-9.
106. Luo J, Nordenvall C, Nyren O, Adami HO, Permert J, Ye W. The risk of pancreatic cancer in patients with gastric or duodenal ulcer disease. Int J Cancer. 2007;120(2):368-72.
107. Hwang IC, Chang J, Park SM. Association between proton pump inhibitor use and the risk of pancreatic cancer: A Korean nationwide cohort study. PLoS One. 2018;13(9):e0203918.

108. Hicks B, Friis S, Pottegard A. Use of proton pump inhibitors and risk of pancreatic cancer. Pharmacoepidemiol Drug Saf. 2018;27(8):926-30.
109. Bosetti C, Lucenteforte E, Bracci PM, Negri E, Neale RE, Risch HA, et al. Ulcer, gastric surgery and pancreatic cancer risk: an analysis from the International Pancreatic Cancer Case-Control Consortium (PanC4). Ann Oncol. 2013;24(11):2903-10.
110. Alguacil J, Kauppinen T, Porta M, Partanen T, Malats N, Kogevinas M, et al. Risk of pancreatic cancer and occupational exposures in Spain. PANKRAS II Study Group. Ann Occup Hyg. 2000;44(5):391-403.
111. Andreotti G, Freeman LE, Hou L, Coble J, Rusiecki J, Hoppin JA, et al. Agricultural pesticide use and pancreatic cancer risk in the Agricultural Health Study Cohort. Int J Cancer. 2009;124(10):2495-500.
112. Andreotti G, Silverman DT. Occupational risk factors and pancreatic cancer: a review of recent findings. Mol Carcinog. 2012;51(1):98-108.
113. Ojajarvi A, Partanen T, Ahlbom A, Hakulinen T, Kauppinen T, Weiderpass E, et al. Estimating the relative risk of pancreatic cancer associated with exposure agents in job title data in a hierarchical Bayesian meta-analysis. Scand J Work Environ Health. 2007;33(5):325-35.
114. Hoppin JA, Tolbert PE, Holly EA, Brock JW, Korrick SA, Altshul LM, et al. Pancreatic cancer and serum organochlorine levels. Cancer Epidemiol Biomarkers Prev. 2000;9(2):199-205.
115. Ji BT, Silverman DT, Stewart PA, Blair A, Swanson GM, Baris D, et al. Occupational exposure to pesticides and pancreatic cancer. Am J Ind Med. 2001;39(1):92-9.
116. Lowenfels AB, Maisonneuve P. Epidemiology and risk factors for pancreatic cancer. Best Pract Res Clin Gastroenterol. 2006;20(2):197-209.
117. Wogan GN, Hecht SS, Felton JS, Conney AH, Loeb LA. Environmental and chemical carcinogenesis. Semin Cancer Biol. 2004;14(6):473-86.
118. Antwi SO, Eckert EC, Sabaque CV, Leof ER, Hawthorne KM, Bamlet WR, et al. Exposure to environmental chemicals and heavy metals, and risk of pancreatic cancer. Cancer Causes Control. 2015;26(11):1583-91.
119. Kachuri L, Harris MA, MacLeod JS, Tjepkema M, Peters PA, Demers PA. Cancer risks in a population-based study of 70,570 agricultural workers: results from the Canadian census health and Environment cohort (CanCHEC). BMC Cancer. 2017;17(1):343.
120. Kauppinen T, Partanen T, Degerth R, Ojajarvi A. Pancreatic cancer and occupational exposures. Epidemiology. 1995;6(5):498-502.
121. Review SCS. SEER Cancer Statistics Review 1975-2017 National Cancer Institue, Bethesda, MD2018 [Available from: https://seer.cancer.gov/csr/1975_2017/browse_csr.php.
122. Ilic M, Ilic I. Epidemiology of pancreatic cancer. World J Gastroenterol. 2016;22(44):9694-705.
123. Fernandez E, La Vecchia C, D'Avanzo B, Negri E, Franceschi S. Family history and the risk of liver, gallbladder, and pancreatic cancer. Cancer Epidemiol Biomarkers Prev. 1994;3(3):209-12.
124. Catts ZA, Baig MK, Milewski B, Keywan C, Guarino M, Petrelli N. Statewide Retrospective Review of Familial Pancreatic Cancer in Delaware, and Frequency of Genetic Mutations in Pancreatic Cancer Kindreds. Ann Surg Oncol. 2016;23(5):1729-35.
125. Zhan HX, Cong L, Zhao YP, Zhang TP, Chen G. Risk factors for the occurrence of insulinoma: a case-control study. Hepatobiliary Pancreat Dis Int. 2013;12(3):324-8.
126. Schenk M, Schwartz AG, O'Neal E, Kinnard M, Greenson JK, Fryzek JP, et al. Familial risk of pancreatic cancer. J Natl Cancer Inst. 2001;93(8):640-4.
127. Fernandez E, La Vecchia C, Decarli A. Attributable risks for pancreatic cancer in northern Italy. Cancer Epidemiol Biomarkers Prev. 1996;5(1):23-7.
128. Paiella S, Capurso G, Cavestro GM, Butturini G, Pezzilli R, Salvia R, et al. Results of First-Round of Surveillance in Individuals at High-Risk of Pancreatic Cancer from the AISP (Italian Association for the Study of the Pancreas) Registry. Am J Gastroenterol. 2019;114(4):665-70.

129. Mocci E, Guillen-Ponce C, Earl J, Marquez M, Solera J, Salazar-Lopez MT, et al. PanGen-Fam: Spanish registry of hereditary pancreatic cancer. Eur J Cancer. 2015;51(14):1911-7.
130. Zheng Z, Zheng R, He Y, Sun X, Wang N, Chen T, et al. Risk Factors for Pancreatic Cancer in China: A Multicenter Case-Control Study. J Epidemiol. 2016;26(2):64-70.
131. Schulte A, Pandeya N, Fawcett J, Fritschi L, Klein K, Risch HA, et al. Association between family cancer history and risk of pancreatic cancer. Cancer Epidemiol. 2016;45:145-50.
132. Hamada T, Yuan C, Yurgelun MB, Perez K, Khalaf N, Morales-Oyarvide V, et al. Family history of cancer, Ashkenazi Jewish ancestry, and pancreatic cancer risk. Br J Cancer. 2019;120(8):848-54.
133. Klein AP, Brune KA, Petersen GM, Goggins M, Tersmette AC, Offerhaus GJ, et al. Prospective risk of pancreatic cancer in familial pancreatic cancer kindreds. Cancer Res. 2004;64(7):2634-8.
134. Piciucchi M, Capurso G, Valente R, Larghi A, Archibugi L, Signoretti M, et al. Early onset pancreatic cancer: risk factors, presentation and outcome. Pancreatology. 2015;15(2):151-5.
135. Matsubayashi H, Takaori K, Morizane C, Maguchi H, Mizuma M, Takahashi H, et al. Familial pancreatic cancer: Concept, management and issues. World J Gastroenterol. 2017;23(6):935-48.
136. Moran A, O'Hara C, Khan S, Shack L, Woodward E, Maher ER, et al. Risk of cancer other than breast or ovarian in individuals with BRCA1 and BRCA2 mutations. Fam Cancer. 2012;11(2):235-42.
137. Mersch J, Jackson MA, Park M, Nebgen D, Peterson SK, Singletary C, et al. Cancers associated with BRCA1 and BRCA2 mutations other than breast and ovarian. Cancer. 2015;121(2):269-75.
138. Bannon SA, Montiel MF, Goldstein JB, Dong W, Mork ME, Borras E, et al. High Prevalence of Hereditary Cancer Syndromes and Outcomes in Adults with Early-Onset Pancreatic Cancer. Cancer Prev Res (Phila). 2018;11(11):679-86.
139. Axilbund JE, Argani P, Kamiyama M, Palmisano E, Raben M, Borges M, et al. Absence of germline BRCA1 mutations in familial pancreatic cancer patients. Cancer Biol Ther. 2009;8(2):131-5.
140. Roberts NJ, Jiao Y, Yu J, Kopelovich L, Petersen GM, Bondy ML, et al. ATM mutations in patients with hereditary pancreatic cancer. Cancer Discov. 2012;2(1):41-6.
141. Kamisawa T, Wood LD, Itoi T, Takaori K. Pancreatic cancer. Lancet. 2016;388(10039):73-85.
142. Slater EP, Langer P, Niemczyk E, Strauch K, Butler J, Habbe N, et al. PALB2 mutations in European familial pancreatic cancer families. Clin Genet. 2010;78(5):490-4.
143. Lynch HT, Deters CA, Snyder CL, Lynch JF, Villeneuve P, Silberstein J, et al. BRCA1 and pancreatic cancer: pedigree findings and their causal relationships. Cancer Genet Cytogenet. 2005;158(2):119-25.
144. Vasen HF, Gruis NA, Frants RR, van Der Velden PA, Hille ET, Bergman W. Risk of developing pancreatic cancer in families with familial atypical multiple mole melanoma associated with a specific 19 deletion of p16 (p16-Leiden). Int J Cancer. 2000;87(6):809-11.
145. Lynch HT, Fusaro RM, Lynch JF, Brand R. Pancreatic cancer and the FAMMM syndrome. Fam Cancer. 2008;7(1):103-12.
146. Giardiello FM, Brensinger JD, Tersmette AC, Goodman SN, Petersen GM, Booker SV, et al. Very high risk of cancer in familial Peutz-Jeghers syndrome. Gastroenterology. 2000;119(6):1447-53.
147. Lowenfels AB, Maisonneuve P, DiMagno EP, Elitsur Y, Gates LK, Jr., Perrault J, et al. Hereditary pancreatitis and the risk of pancreatic cancer. International Hereditary Pancreatitis Study Group. J Natl Cancer Inst. 1997;89(6):442-6.
148. Howes N, Lerch MM, Greenhalf W, Stocken DD, Ellis I, Simon P, et al. Clinical and genetic characteristics of hereditary pancreatitis in Europe. Clin Gastroenterol Hepatol. 2004;2(3):252-61.
149. Rebours V, Boutron-Ruault MC, Schnee M, Ferec C, Maire F, Hammel P, et al. Risk of pancreatic adenocarcinoma in patients with hereditary pancreatitis: a national exhaustive series. Am J Gastroenterol. 2008;103(1):111-9.
150. Whitcomb DC, Applebaum S, Martin SP. Hereditary pancreatitis and pancreatic carcinoma. Ann N Y Acad Sci. 1999;880:201-9.

151. Aarnio M, Sankila R, Pukkala E, Salovaara R, Aaltonen LA, de la Chapelle A, et al. Cancer risk in mutation carriers of DNA-mismatch-repair genes. Int J Cancer. 1999;81(2):214-8.
152. Ben Q, Xu M, Ning X, Liu J, Hong S, Huang W, et al. Diabetes mellitus and risk of pancreatic cancer: A meta-analysis of cohort studies. Eur J Cancer. 2011;47(13):1928-37.
153. Er KC, Hsu CY, Lee YK, Huang MY, Su YC. Effect of glycemic control on the risk of pancreatic cancer: A nationwide cohort study. Medicine (Baltimore). 2016;95(24):e3921.
154. Pang Y, Kartsonaki C, Guo Y, Bragg F, Yang L, Bian Z, et al. Diabetes, plasma glucose and incidence of pancreatic cancer: A prospective study of 0.5 million Chinese adults and a meta-analysis of 22 cohort studies. Int J Cancer. 2017;140(8):1781-8.
155. Wolpin BM, Bao Y, Qian ZR, Wu C, Kraft P, Ogino S, et al. Hyperglycemia, insulin resistance, impaired pancreatic beta-cell function, and risk of pancreatic cancer. J Natl Cancer Inst. 2013;105(14):1027-35.
156. Austin MA, Kuo E, Van Den Eeden SK, Mandelson MT, Brentnall TA, Kamineni A, et al. Family history of diabetes and pancreatic cancer as risk factors for pancreatic cancer: the PACIFIC study. Cancer Epidemiol Biomarkers Prev. 2013;22(10):1913-7.
157. Perrin MC, Terry MB, Kleinhaus K, Deutsch L, Yanetz R, Tiram E, et al. Gestational diabetes as a risk factor for pancreatic cancer: a prospective cohort study. BMC Med. 2007;5:25.
158. Ben Q, Cai Q, Li Z, Yuan Y, Ning X, Deng S, et al. The relationship between new-onset diabetes mellitus and pancreatic cancer risk: a case-control study. Eur J Cancer. 2011;47(2):248-54.
159. Johnson JA, Bowker SL, Richardson K, Marra CA. Time-varying incidence of cancer after the onset of type 2 diabetes: evidence of potential detection bias. Diabetologia. 2011;54(9):2263-71.
160. Setiawan VW, Stram DO, Porcel J, Chari ST, Maskarinec G, Le Marchand L, et al. Pancreatic Cancer Following Incident Diabetes in African Americans and Latinos: The Multiethnic Cohort. J Natl Cancer Inst. 2019;111(1):27-33.
161. Wang F, Gupta S, Holly EA. Diabetes mellitus and pancreatic cancer in a population-based case-control study in the San Francisco Bay Area, California. Cancer Epidemiol Biomarkers Prev. 2006;15(8):1458-63.
162. Li D. Diabetes and pancreatic cancer. Mol Carcinog. 2012;51(1):64-74.
163. Song S, Wang B, Zhang X, Hao L, Hu X, Li Z, et al. Long-Term Diabetes Mellitus Is Associated with an Increased Risk of Pancreatic Cancer: A Meta-Analysis. PLoS One. 2015;10(7):e0134321.
164. Elena JW, Steplowski E, Yu K, Hartge P, Tobias GS, Brotzman MJ, et al. Diabetes and risk of pancreatic cancer: a pooled analysis from the pancreatic cancer cohort consortium. Cancer Causes Control. 2013;24(1):13-25.
165. Arslan AA, Helzlsouer KJ, Kooperberg C, Shu XO, Steplowski E, Bueno-de-Mesquita HB, et al. Anthropometric measures, body mass index, and pancreatic cancer: a pooled analysis from the Pancreatic Cancer Cohort Consortium (PanScan). Arch Intern Med. 2010;170(9):791-802.
166. Janghorbani M, Dehghani M, Salehi-Marzijarani M. Systematic review and meta-analysis of insulin therapy and risk of cancer. Horm Cancer. 2012;3(4):137-46.
167. Currie CJ, Poole CD, Gale EA. The influence of glucose-lowering therapies on cancer risk in type 2 diabetes. Diabetologia. 2009;52(9):1766-77.
168. Soranna D, Scotti L, Zambon A, Bosetti C, Grassi G, Catapano A, et al. Cancer risk associated with use of metformin and sulfonylurea in type 2 diabetes: a meta-analysis. Oncologist. 2012;17(6):813-22.
169. Bosetti C, Rosato V, Li D, Silverman D, Petersen GM, Bracci PM, et al. Diabetes, antidiabetic medications, and pancreatic cancer risk: an analysis from the International Pancreatic Cancer Case-Control Consortium. Ann Oncol. 2014;25(10):2065-72.
170. Karp I, Sivaswamy A, Booth C. Does the use of incretin-based medications increase the risk of cancer in patients with type-2 diabetes mellitus? Pharmacoepidemiol Drug Saf. 2019;28(4):489-99.
171. Buse JB, Bethel MA, Green JB, Stevens SR, Lokhnygina Y, Aschner P, et al. Pancreatic Safety of Sitagliptin in the TECOS Study. Diabetes Care. 2017;40(2):164-70.
172. Lee M, Sun J, Han M, Cho Y, Lee JY, Nam CM, et al. Nationwide Trends in Pancreatitis and Pancreatic Cancer Risk Among Patients With Newly Diagnosed Type 2 Diabetes Receiving Dipeptidyl Peptidase 4 Inhibitors. Diabetes Care. 2019;42(11):2057-64.

173. Raz I, Bhatt DL, Hirshberg B, Mosenzon O, Scirica BM, Umez-Eronini A, et al. Incidence of pancreatitis and pancreatic cancer in a randomized controlled multicenter trial (SAVOR-TIMI 53) of the dipeptidyl peptidase-4 inhibitor saxagliptin. Diabetes Care. 2014;37(9):2435-41.
174. Van Hemelrijck M, Holmberg L, Garmo H, Hammar N, Walldius G, Binda E, et al. Association between levels of C-reactive protein and leukocytes and cancer: three repeated measurements in the Swedish AMORIS study. Cancer Epidemiol Biomarkers Prev. 2011;20(3):428-37.
175. Ghuman S, Van Hemelrijck M, Garmo H, Holmberg L, Malmstrom H, Lambe M, et al. Serum inflammatory markers and colorectal cancer risk and survival. Br J Cancer. 2017;116(10):1358-65.
176. Sollie S, Michaud DS, Sarker D, Karagiannis SN, Josephs DH, Hammar N, et al. Chronic inflammation markers are associated with risk of pancreatic cancer in the Swedish AMORIS cohort study. BMC Cancer. 2019;19(1):858.
177. Grote VA, Kaaks R, Nieters A, Tjonneland A, Halkjaer J, Overvad K, et al. Inflammation marker and risk of pancreatic cancer: a nested case-control study within the EPIC cohort. Br J Cancer. 2012;106(11):1866-74.
178. Padoan A, Plebani M, Basso D. Inflammation and Pancreatic Cancer: Focus on Metabolism, Cytokines, and Immunity. Int J Mol Sci. 2019;20(3).
179. Ekbom A, McLaughlin JK, Karlsson BM, Nyren O, Gridley G, Adami HO, et al. Pancreatitis and pancreatic cancer: a population-based study. J Natl Cancer Inst. 1994;86(8):625-7.
180. Goldacre MJ, Wotton CJ, Yeates D, Seagroatt V, Collier J. Liver cirrhosis, other liver diseases, pancreatitis and subsequent cancer: record linkage study. Eur J Gastroenterol Hepatol. 2008;20(5):384-92.
181. Fernandez E, La Vecchia C, Porta M, Negri E, d'Avanzo B, Boyle P. Pancreatitis and the risk of pancreatic cancer. Pancreas. 1995;11(2):185-9.
182. Lowenfels AB, Maisonneuve P, Cavallini G, Ammann RW, Lankisch PG, Andersen JR, et al. Pancreatitis and the risk of pancreatic cancer. International Pancreatitis Study Group. N Engl J Med. 1993;328(20):1433-7.
183. Malka D, Hammel P, Maire F, Rufat P, Madeira I, Pessione F, et al. Risk of pancreatic adenocarcinoma in chronic pancreatitis. Gut. 2002;51(6):849-52.
184. Midha S, Sreenivas V, Kabra M, Chattopadhyay TK, Joshi YK, Garg PK. Genetically Determined Chronic Pancreatitis but not Alcoholic Pancreatitis Is a Strong Risk Factor for Pancreatic Cancer. Pancreas. 2016;45(10):1478-84.
185. Duell EJ, Lucenteforte E, Olson SH, Bracci PM, Li D, Risch HA, et al. Pancreatitis and pancreatic cancer risk: a pooled analysis in the International Pancreatic Cancer Case-Control Consortium (PanC4). Ann Oncol. 2012;23(11):2964-70.
186. Bansal P, Sonnenberg A. Pancreatitis is a risk factor for pancreatic cancer. Gastroenterology. 1995;109(1):247-51.
187. Bracci PM, Wang F, Hassan MM, Gupta S, Li D, Holly EA. Pancreatitis and pancreatic cancer in two large pooled case-control studies. Cancer Causes Control. 2009;20(9):1723-31.
188. Karlson BM, Ekbom A, Josefsson S, McLaughlin JK, Fraumeni JF, Jr., Nyren O. The risk of pancreatic cancer following pancreatitis: an association due to confounding? Gastroenterology. 1997;113(2):587-92.
189. Talamini G, Falconi M, Bassi C, Sartori N, Salvia R, Caldiron E, et al. Incidence of cancer in the course of chronic pancreatitis. Am J Gastroenterol. 1999;94(5):1253-60.
190. Ueda J, Tanaka M, Ohtsuka T, Tokunaga S, Shimosegawa T, Research Committee of Intractable Diseases of the P. Surgery for chronic pancreatitis decreases the risk for pancreatic cancer: a multicenter retrospective analysis. Surgery. 2013;153(3):357-64.

191. Raimondi S, Lowenfels AB, Morselli-Labate AM, Maisonneuve P, Pezzilli R. Pancreatic cancer in chronic pancreatitis; aetiology, incidence, and early detection. Best Pract Res Clin Gastroenterol. 2010;24(3):349-58.
192. Pak LM, Schattner MA, Balachandran V, D'Angelica MI, DeMatteo RP, Kingham TP, et al. The clinical utility of immunoglobulin G4 in the evaluation of autoimmune pancreatitis and pancreatic adenocarcinoma. HPB (Oxford). 2018;20(2):182-7.
193. Raina A, Krasinskas AM, Greer JB, Lamb J, Fink E, Moser AJ, et al. Serum immunoglobulin G fraction 4 levels in pancreatic cancer: elevations not associated with autoimmune pancreatitis. Arch Pathol Lab Med. 2008;132(1):48-53.

194. Ngwa T, Law R, Hart P, Smyrk TC, Chari ST. Serum IgG4 elevation in pancreatic cancer: diagnostic and prognostic significance and association with autoimmune pancreatitis. Pancreas. 2015;44(4):557-60.
195. Chang MC, Liang PC, Jan S, Yang CY, Tien YW, Wei SC, et al. Increase diagnostic accuracy in differentiating focal type autoimmune pancreatitis from pancreatic cancer with combined serum IgG4 and CA19-9 levels. Pancreatology. 2014;14(5):366-72.
196. Talar-Wojnarowska R, Gasiorowska A, Olakowski M, Dranka-Bojarowska D, Lampe P, Smigielski J, et al. Utility of serum IgG, IgG4 and carbonic anhydrase II antibodies in distinguishing autoimmune pancreatitis from pancreatic cancer and chronic pancreatitis. Adv Med Sci. 2014;59(2):288-92.
197. Sollie S, Santaolalla A, Michaud DS, Sarker D, Karagiannis SN, Josephs DH, et al. Serum Immunoglobulin G Is Associated With Decreased Risk of Pancreatic Cancer in the Swedish AMORIS Study. Front Oncol. 2020;10:263.
198. Turner MC. Epidemiology: allergy history, IgE, and cancer. Cancer Immunol Immunother. 2012;61(9):1493-510.
199. Anderson LN, Cotterchio M, Gallinger S. Lifestyle, dietary, and medical history factors associated with pancreatic cancer risk in Ontario, Canada. Cancer Causes Control. 2009;20(6):825-34.
200. Cotterchio M, Lowcock E, Hudson TJ, Greenwood C, Gallinger S. Association between allergies and risk of pancreatic cancer. Cancer Epidemiol Biomarkers Prev. 2014;23(3):469-80.
201. Gandini S, Lowenfels AB, Jaffee EM, Armstrong TD, Maisonneuve P. Allergies and the risk of pancreatic cancer: a meta-analysis with review of epidemiology and biological mechanisms. Cancer Epidemiol Biomarkers Prev. 2005;14(8):1908-16.
202. Gomez-Rubio P, Zock JP, Rava M, Marquez M, Sharp L, Hidalgo M, et al. Reduced risk of pancreatic cancer associated with asthma and nasal allergies. Gut. 2017;66(2):314-22.
203. Eppel A, Cotterchio M, Gallinger S. Allergies are associated with reduced pancreas cancer risk: A population-based case-control study in Ontario, Canada. Int J Cancer. 2007;121(10):2241-5.
204. Olson SH. Selected medical conditions and risk of pancreatic cancer. Mol Carcinog. 2012;51(1):75-97.
205. Kreiger N, Lacroix J, Sloan M. Hormonal factors and pancreatic cancer in women. Ann Epidemiol. 2001;11(8):563-7.
206. Lucenteforte E, Zucchetto A, Bosetti C, Talamini R, Negri E, Serraino D, et al. Reproductive and hormonal factors and pancreatic cancer risk in women. Pancreas. 2011;40(3):460-3.
207. Skinner HG, Michaud DS, Colditz GA, Giovannucci EL, Stampfer MJ, Willett WC, et al. Parity, reproductive factors, and the risk of pancreatic cancer in women. Cancer Epidemiol Biomarkers Prev. 2003;12(5):433-8.
208. Zhang Y, Coogan PF, Palmer JR, Strom BL, Rosenberg L. A case-control study of reproductive factors, female hormone use, and risk of pancreatic cancer. Cancer Causes Control. 2010;21(3):473-8.
209. Karlson BM, Wuu J, Hsieh CC, Lambe M, Ekbom A. Parity and the risk of pancreatic cancer: a nested case-control study. Int J Cancer. 1998;77(2):224-7.
210. Andersson G, Borgquist S, Jirstrom K. Hormonal factors and pancreatic cancer risk in women: The Malmo Diet and Cancer Study. Int J Cancer. 2018;143(1):52-62.
211. Bueno de Mesquita HB, Maisonneuve P, Moerman CJ, Walker AM. Anthropometric and reproductive variables and exocrine carcinoma of the pancreas: a population-based case-control study in The Netherlands. Int J Cancer. 1992;52(1):24-9.
212. Fernandez E, La Vecchia C, D'Avanzo B, Negri E. Menstrual and reproductive factors and pancreatic cancer risk in women. Int J Cancer. 1995;62(1):11-4.
213. Duell EJ, Holly EA. Reproductive and menstrual risk factors for pancreatic cancer: a population-based study of San Francisco Bay Area women. Am J Epidemiol. 2005;161(8):741-7.
214. Berrington de Gonzalez A, Sweetland S, Spencer E. A meta-analysis of obesity and the risk of pancreatic cancer. Br J Cancer. 2003;89(3):519-23.
215. Stolzenberg-Solomon RZ, Schairer C, Moore S, Hollenbeck A, Silverman DT. Lifetime adiposity and risk of pancreatic cancer in the NIH-AARP Diet and Health Study cohort. Am J Clin Nutr. 2013;98(4):1057-65.
216. Larsson SC, Orsini N, Wolk A. Body mass index and pancreatic cancer risk: A meta-analysis of prospective studies. Int J Cancer. 2007;120(9):1993-8.

217. Jiao L, Berrington de Gonzalez A, Hartge P, Pfeiffer RM, Park Y, Freedman DM, et al. Body mass index, effect modifiers, and risk of pancreatic cancer: a pooled study of seven prospective cohorts. Cancer Causes Control. 2010;21(8):1305-14.
218. Noor NM, Banim PJ, Luben RN, Khaw KT, Hart AR. Investigating Physical Activity in the Etiology of Pancreatic Cancer: The Age at Which This Is Measured Is Important and Is Independent of Body Mass Index. Pancreas. 2016;45(3):388-93.
219. Kabat GC, Kim MY, Chlebowski RT, Vitolins MZ, Wassertheil-Smoller S, Rohan TE. Serum lipids and risk of obesity-related cancers in postmenopausal women. Cancer Causes Control. 2018;29(1):13-24.
220. La Torre G, Sferrazza A, Gualano MR, de Waure C, Clemente G, De Rose AM, et al. Investigating the synergistic interaction of diabetes, tobacco smoking, alcohol consumption, and hypercholesterolemia on the risk of pancreatic cancer: a case-control study in Italy. Biomed Res Int. 2014;2014:481019.
221. Rosato V, Tavani A, Bosetti C, Pelucchi C, Talamini R, Polesel J, et al. Metabolic syndrome and pancreatic cancer risk: a case-control study in Italy and meta-analysis. Metabolism. 2011;60(10):1372-8.
222. Zaleska M, Mozenska O, Bil J. Statins use and cancer: an update. Future Oncol. 2018;14(15):1497-509.
223. Bradley MC, Hughes CM, Cantwell MM, Murray LJ. Statins and pancreatic cancer risk: a nested case-control study. Cancer Causes Control. 2010;21(12):2093-100.
224. Carey FJ, Little MW, Pugh TF, Ndokera R, Ing H, Clark A, et al. The differential effects of statins on the risk of developing pancreatic cancer: a case-control study in two centres in the United Kingdom. Dig Dis Sci. 2013;58(11):3308-12.
225. Chiu HF, Chang CC, Ho SC, Wu TN, Yang CY. Statin use and the risk of pancreatic cancer: a population-based case-control study. Pancreas. 2011;40(5):669-72.
226. Cui X, Xie Y, Chen M, Li J, Liao X, Shen J, et al. Statin use and risk of pancreatic cancer: a meta-analysis. Cancer Causes Control. 2012;23(7):1099-111.
227. Kirkegard J, Lund JL, Mortensen FV, Cronin-Fenton D. Statins and pancreatic cancer risk in patients with chronic pancreatitis: A Danish nationwide population-based cohort study. Int J Cancer. 2020;146(3):610-6.
228. Esposito K, Giugliano D. The metabolic syndrome and inflammation: association or causation? Nutr Metab Cardiovasc Dis. 2004;14(5):228-32.
229. Inoue M, Noda M, Kurahashi N, Iwasaki M, Sasazuki S, Iso H, et al. Impact of metabolic factors on subsequent cancer risk: results from a large-scale population-based cohort study in Japan. Eur J Cancer Prev. 2009;18(3):240-7.
230. Risch HA, Yu H, Lu L, Kidd MS. ABO blood group, Helicobacter pylori seropositivity, and risk of pancreatic cancer: a case-control study. J Natl Cancer Inst. 2010;102(7):502-5.
231. Risch HA, Lu L, Wang J, Zhang W, Ni Q, Gao YT, et al. ABO blood group and risk of pancreatic cancer: a study in Shanghai and meta-analysis. Am J Epidemiol. 2013;177(12):1326-37.
232. Wolpin BM, Kraft P, Gross M, Helzlsouer K, Bueno-de-Mesquita HB, Steplowski E, et al. Pancreatic cancer risk and ABO blood group alleles: results from the pancreatic cancer cohort consortium. Cancer Res. 2010;70(3):1015-23.
233. Tada M, Kawabe T, Arizumi M, Togawa O, Matsubara S, Yamamoto N, et al. Pancreatic cancer in patients with pancreatic cystic lesions: a prospective study in 197 patients. Clin Gastroenterol Hepatol. 2006;4(10):1265-70.
234. Matsubara S, Tada M, Akahane M, Yagioka H, Kogure H, Sasaki T, et al. Incidental pancreatic cysts found by magnetic resonance imaging and their relationship with pancreatic cancer. Pancreas. 2012;41(8):1241-6.
235. Bray F, Ferlay J, Soerjomataram I, Siegel RL, Torre LA, Jemal A. Global cancer statistics 2018: GLOBOCAN estimates of incidence and mortality worldwide for 36 cancers in 185 countries. CA Cancer J Clin. 2018;68(6):394-424.
236. Zambirinis CP, Pushalkar S, Saxena D, Miller G. Pancreatic cancer, inflammation, and microbiome. Cancer J. 2014;20(3):195-202.
237. Singh GK, Jemal A. Socioeconomic and Racial/Ethnic Disparities in Cancer Mortality, Incidence, and Survival in the United States, 1950-2014: Over Six Decades of Changing Patterns and Widening Inequalities. J Environ Public Health. 2017;2017:2819372.
238. Lu PY, Shu L, Shen SS, Chen XJ, Zhang XY. Dietary Patterns and Pancreatic Cancer Risk: A Meta-Analysis. Nutrients. 2017;9(1).

239. Ghadirian P, Baillargeon J, Simard A, Perret C. Food habits and pancreatic cancer: a case-control study of the Francophone community in Montreal, Canada. Cancer Epidemiol Biomarkers Prev. 1995;4(8):895-9.
240. Abdelrehim MG, Mahfouz EM, Ewis AA, Seedhom AE, Afifi HM, Shebl FM. Dietary Factors Associated with Pancreatic Cancer Risk in Minia, Egypt: Principal Component Analysis. Asian Pac J Cancer Prev. 2018;19(2):449-55.
241. Bidel S, Hu G, Jousilahti P, Pukkala E, Hakulinen T, Tuomilehto J. Coffee consumption and risk of gastric and pancreatic cancer--a prospective cohort study. Int J Cancer. 2013;132(7):1651-9.
242. Ko AH, Wang F, Holly EA. Pancreatic cancer and medical history in a population-based case-control study in the San Francisco Bay Area, California. Cancer Causes Control. 2007;18(8):809-19.
243. Dai Q, Zheng W, Ji BT, Shu XO, Jin F, Zhu JL, et al. Prior immunity-related medical conditions and pancreatic-cancer risk in Shanghai. Int J Cancer. 1995;63(3):337-40.
244. Tseng CH. Sitagliptin and pancreatic cancer risk in patients with type 2 diabetes. Eur J Clin Invest. 2016;46(1):70-9.
245. Zheng J, Guinter MA, Merchant AT, Wirth MD, Zhang J, Stolzenberg-Solomon RZ, et al. Dietary patterns and risk of pancreatic cancer: a systematic review. Nutr Rev. 2017;75(11):883-908.
246. Kastrinos F, Mukherjee B, Tayob N, Wang F, Sparr J, Raymond VM, et al. Risk of pancreatic cancer in families with Lynch syndrome. JAMA. 2009;302(16):1790-5.
